# Discovery of Hyrtinadine A and Its Derivatives as Novel Antiviral and Anti-Phytopathogenic-Fungus Agents

**DOI:** 10.3390/molecules27238439

**Published:** 2022-12-02

**Authors:** Ji Dong, Henan Ma, Beibei Wang, Shaoxiang Yang, Ziwen Wang, Yongqiang Li, Yuxiu Liu, Qingmin Wang

**Affiliations:** 1 Tianjin Key Laboratory of Structure and Performance for Functional Molecules, College of Chemistry, Tianjin Normal University, Tianjin 300387, China; 2 Beijing Key Laboratory of Flavor Chemistry, Beijing Technology and Business University (BTBU), Beijing 100048, China; 3 State Key Laboratory of Elemento-Organic Chemistry, Research Institute of Elemento-Organic Chemistry, College of Chemistry, Collaborative Innovation Center of Chemical Science and Engineering (Tianjin), Nankai University, Tianjin 300071, China

**Keywords:** alkaloid, antiviral activity, fungicidal activity, hyrtinadine A, marine natural product, structure-activity relationship, structure optimization

## Abstract

Plant diseases caused by viruses and fungi have a serious impact on the quality and yield of crops, endangering food security. The use of new, green, and efficient pesticides is an important strategy to increase crop output and deal with the food crisis. Ideally, the best pesticide innovation strategy is to find and use active compounds from natural products. Here, we took the marine natural product hyrtinadine A as the lead compound, and designed, synthesized, and systematically investigated a series of its derivatives for their antiviral and antifungal activities. Compound **8a** was found to have excellent antiviral activity against the tobacco mosaic virus (TMV) (inactivation inhibitory effect of 55%/500 μg/mL and 19%/100 μg/mL, curative inhibitory effect of 52%/500 μg/mL and 22%/100 μg/mL, and protection inhibitory effect of 57%/500 μg/mL and 26%/100 μg/mL) and emerged as a novel antiviral candidate. These compound derivatives displayed broad-spectrum fungicidal activities against 14 kinds of phytopathogenic fungi at 50 μg/mL and the antifungal activities of compounds **5c**, **5g**, **6a**, and **6e** against *Rhizoctonia cerealis* are higher than that of the commercial fungicide chlorothalonil. Therefore, this study could lay a foundation for the application of hyrtinadine A derivatives in plant protection.

## 1. Introduction

Plant diseases caused by viruses, fungi, and bacteria have long been a great threat to agricultural production [1]. In general, crop losses caused by plant diseases account for 25% of the world’s crop output every year [2]. As the earliest and deepest studied model virus, the tobacco mosaic virus (TMV) has been found to infect more than 400 crops including tobacco, cucumber, banana, etc. [3]. Because the TMV is parasitic to the host cells, and plants have no complete immune system, it is extremely difficult to control the plant diseases caused by the TMV [4]. In addition, the reduction in resources, climate change, and population growth all contribute to the need to maximize crop production to ensure food security [5,6,7]. Therefore, plant disease prevention is becoming increasingly important and urgent. Although scientists have created some bactericides and antiviral agents in the past decades, some of them have developed serious drug resistance during long-term use [8,9]. As a widely used antiviral agent, ribavirin showed a less than 50% anti-TMV effect at 500 μg/mL. With the enhancement of people’s awareness of environmental protection, batches of problematic pesticides have been banned [10]. Thus, the discovery of new, green, and efficient pesticides is becoming increasingly important.

In the vast ocean, there are a large number of secondary metabolites with novel chemical structures, diverse biological activities, and unique mechanisms of action beyond people’s imagination. Marine natural products (MNPs) have become the main source of important drug leads. These compounds have made significant contributions to the treatment of many diseases, including cancer and infectious diseases, as well as other therapeutic areas, such as multidrug resistance (MDR), cardiovascular disease, and multiple sclerosis [11,12]. Compared with traditional synthetic molecules, MNPs have unique structural characteristics and complex skeletons, which are conducive to drug discovery. MNPs usually have a high molecular weight and a large number of carbon and oxygen atoms. In addition, marine bioactive compounds have fewer but unique nitrogen atoms and halogen atoms. To date, more than 20 MNP drugs or pesticides have been approved for use, such as Nereistoxin (insecticide for the control of rice insects), Cytarabine (anticancer drug), Ziconotide (analgesic), Trabectedin (anticancer drug), and Brentuximab (a drug for lymphoma) [13,14,15]. Hyrtinadine A is a bis-indole alkaloid isolated from an Okinawan marine sponge *Hyrtios* sp. (SS-1127; order Dictyoceratida; family Thorectidae; collected off Unten-Port, Okinawa, Japan) [16]. Its novel chemical structure aroused the interest of chemists, and the total synthesis of hyrtinadine A has been successfully completed with the Masuda borylation–Suzuki reaction and Kosugi–Migita–Stille reaction [17,18]. At present, the research on the activity of hyrtinadine A mainly focuses on its anti-tumor properties, and the research on its bioactivity spectrum and structure-activity relationship is relatively lacking. Therefore, it is of considerable significance to carry out research on the synthesis, structure optimization, and biological activity of hyrtinadine A in order to deeply understand its behavior and explore its application.

**Figure 1:**
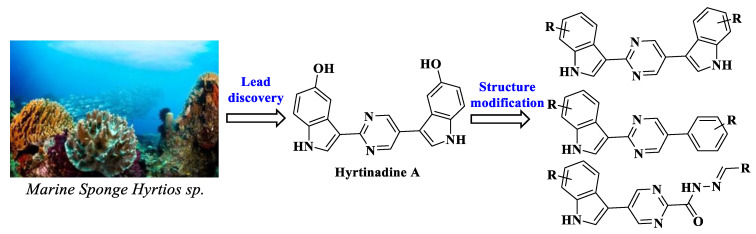
Design of hyrtinadine A derivatives.

We have been committed to the discovery of novel pesticide leads based on MNPs for a long time and have found that a number of marine natural products can be used as candidates for antiviral, fungicidal, and insecticidal agents [19,20,21]. Here, alkaloid hyrtinadine A was selected as the parent structure, and a series of its derivatives were designed (Figure 1), synthesized, and evaluated for their antiviral and antifungal activities.

## 2. Results and Discussion

### 2.1. Chemistry

**Scheme 1:**
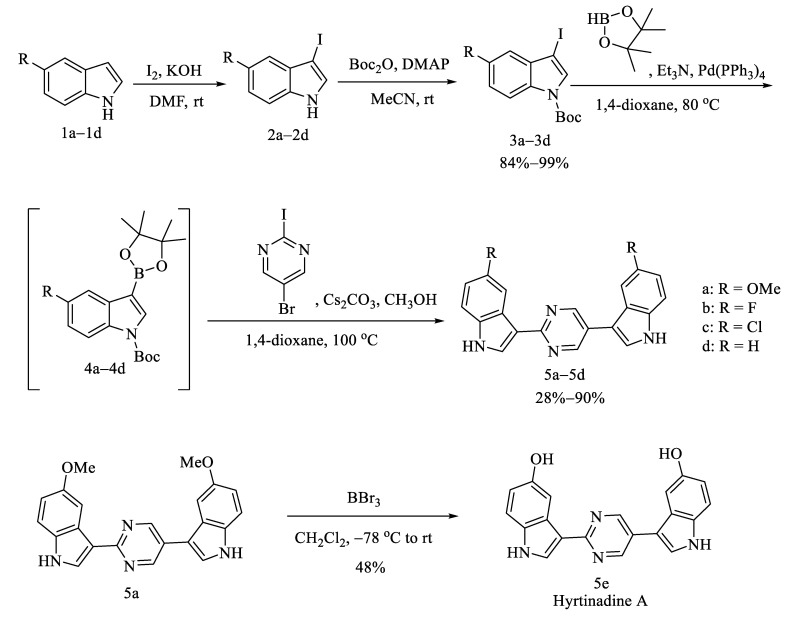
The synthesis of alkaloid hyrtinadine A and compounds **5a**–**5d**.

To date, the total synthesis of hyrtinadine A has been reported by Sarandeses [22], Müller [17], and Söderberg [18]. Müller’s route is more suitable for the large-scale preparation and derivation of hyrtinadine A and can provide intermediates with rich structures. Therefore, hyrtinadine A was prepared in five steps from 5-methoxyindole with a 42% overall yield (Scheme 1) by Müller’s method with modification [17].

**Scheme 2:**
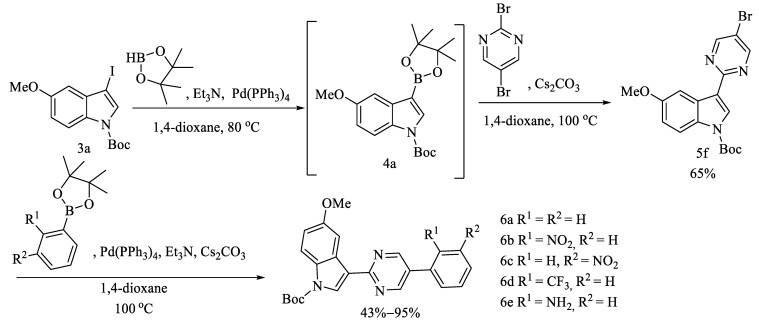
The synthesis of compounds **6a**–**6e**.

**Scheme 3:**
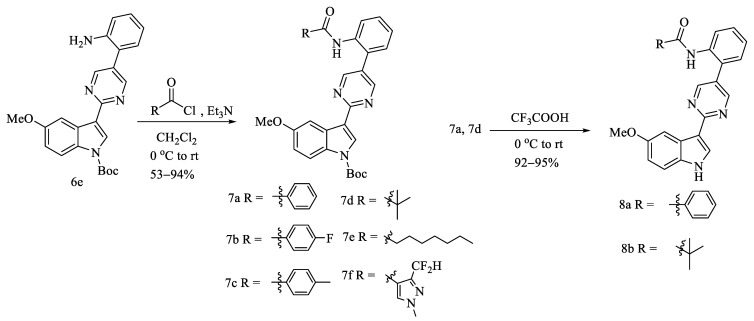
The synthesis of compounds **7a**–**7f** and **8a**–**8b**.

**Scheme 4:**
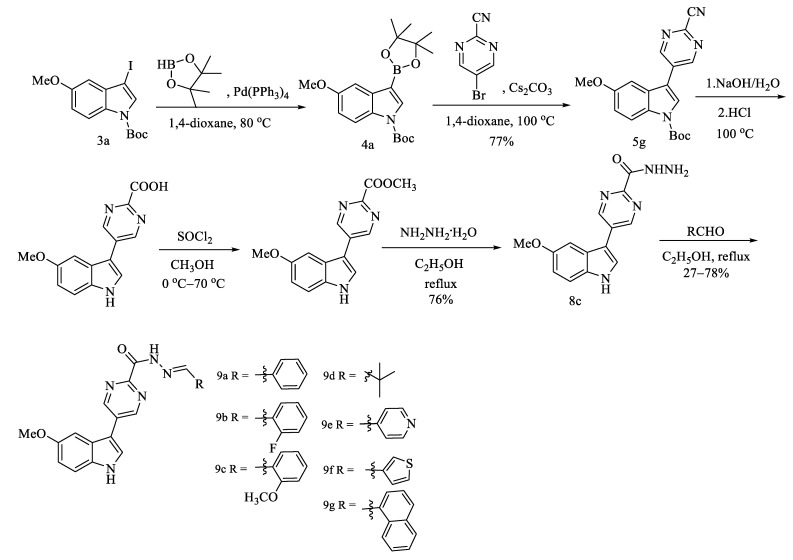
The synthesis of compounds **9a**–**9g**.

In order to investigate the structure-activity relationship (SAR), a series of hyrtinadine A derivatives (**5a**–**5d**, **6a**–**6e**, **7a**–**7f**, **8a**–**8b**, and **9a**–**9g**) were designed and synthesized. As depicted in Scheme 1, compounds **5a**–**5d** with different groups at the indole ring were prepared using the Masuda borylation–Suzuki reaction as a key step. Scaffold hopping is a common and efficient strategy for drug lead optimization [23]. In order to investigate the importance of the bis-indole ring on the compound’s activity and further simplify the molecular structure, we designed and synthesized compounds **6a**–**6e** by replacing the indole ring with a substituted benzene ring (Scheme 2). As shown in Scheme 3, the NH_2_ group in **6e** can be replaced with a corresponding acyl chloride to create mono-acylated products **7a**–**7f**. Then, the protective group of the mono-acylated products **7a** and **7d** could be removed under the condition of CF_3_COOH to create products **8a**–**8b**. Acylhydrazone is a good pharmacophore, and we found that the introduction of this group can improve the antiviral activity of the molecule [24]. As depicted in Scheme 4, compound **5g** went through hydrolysis and esterification hydrazinolysis to yield **8c**. Then, **8c** reacted with the corresponding aldehyde in ethanol under the refluxed condition to yield acylhydrazones **9a**–**9g**.

### 2.2. Phytotoxic Activity

The phytotoxic activity tests demonstrated that compounds **5a**–**5d**, **6a**–**6e**, **7a**–**7f**, **8a**–**8b**, and **9a**–**9g** were safe for testing on plants at 500 μg/mL. The detailed test procedures can be seen in the Supplementary Materials.

### 2.3. Antiviral Activity

#### 2.3.1. In Vivo Anti-TMV Activity

As shown in Table 1, the natural product hyrtinadine A (**5e**) exhibited a certain degree of anti-TMV activity (inactivation inhibitory effect of 11%/500 μg/mL). Most of the hyrtinadine derivatives displayed higher in vivo anti-TMV activities at 500 μg/mL than ribavirin and hyrtinadine A (such as compounds **5a**, **5c**, **5d**, **5g**, **6e**, **7b**, **7c**, **7d**, **7f**, **8a**, **9a**, **9b**, **9d**, **9e**, and **9f**) and some of them even revealed similar activity to ningnanmycin (such as compounds **5a**, **5c**, **8a**, **9e**, and **9f**). Compound **8a** showed the best anti-TMV activity (inactivation inhibitory effect of 55%/500 μg/mL and 19%/100 μg/mL, curative inhibitory effect of 52%/500 μg/mL and 22%/100 μg/mL, and protection inhibitory effect of 57%/500 μg/mL and 26%/100 μg/mL), emerging as a novel antiviral candidate.

#### 2.3.2. Structure-Activity Relationship (SAR)

On the whole, the structural optimization of the natural product hyrtinadine A is very successful, and the biological activities of most derivatives are better than that of hyrtinadine A. The substituent groups at the 5-position of the indole had a significant influence on biological activity, and methoxy and chlorine are the most prominent (inhibitory effect: **5a** ≈ **5c** > **5d** > **5b** > **5e**). The electron effect does not show obvious regularity. The substitution of Br or CN for an indole ring leads to a decrease in activity (inhibitory effect: **5a** > **5f** and **5g**), which indicates that an aromatic ring in this area is necessary. A substituted benzene ring instead of an indole ring on hyrtinadine A is also unfavorable to its activity (inhibitory effect: **5a** > **6a**–**6c** and **6e**). The introduction of an acyl group into the benzene ring amino group has little effect on the activity (inhibitory effect: **6e** > **7a**–**7f**). In contrast, increasing the electron cloud density on the benzene ring is beneficial to its biological activity (inhibitory effect: **6e** > **6a**–**6c** and **6e**). The activity of compound **7a** was significantly increased after further deprotection (inhibitory effect: **8a** > **7a**), but the activity of compound **7d** decreased after deprotection (inhibitory effect: **7d** > **8b**). Acylhydrazone is a good pharmacophore; most of the compounds containing acylhydrazone functional groups show good antiviral activity (inhibitory effect: **9e**, **9f** ≈ **5a** > **9a**, **9b**, **9d** ≈ **5g** > **9c**, **9g**). The successful discovery of highly active molecules **8a**, **9e**, and **9f** provides guidance for us to further simplify the molecular structure.

### 2.4. Fungicidal Activity

Hyrtinadine A compounds **5a**–**5d**, **6a**–**6e**, **7a**–**7f**, **8a**–**8b**, and **9a**–**9g** were also evaluated for their fungicidal activities and compared with commercial fungicides carbendazim and chlorothalonil as controls. As shown in Table 2, all synthesized compounds displayed broad-spectrum fungicidal activities against 14 kinds of phytopathogenic fungi at 50 μg/mL. Almost all the compounds displayed near or more than a 60% inhibition rate against *Physalospora piricola* and *Rhizoctonia cerealis*. The antifungal activities of **5c**, **5g**, **6a**, and **6e** against *Rhizoctonia cerealis* are higher than that of chlorothalonil. In addition, compounds **5b** and **5c** displayed higher fungicidal activities against *Alternaria solani* than carbendazim and chlorothalonil. The above results indicate that these compounds have the potential to be used as biological fungicides.

## 3. Materials and Methods

### 3.1. Synthetic Procedures

#### 3.1.1. Chemicals

Reagents were purchased from commercial sources and were used as received. All anhydrous solvents were dried and purified by standard techniques prior to use.

#### 3.1.2. Instruments

The melting points of the synthesized compounds were determined on an X-4 binocular microscope (Beijing Tech Instruments Co., Beijing, China) with the thermometer uncorrected. NMR spectra were obtained on a Bruker AV 400 spectrometer (Bruker Corp., Switzerland) in CDCl_3_ or DMSO-*d*_6_ solution with tetramethylsilane as the internal standard. High-resolution mass spectra were obtained with an FT-ICR MS spectrometer (Ionspec, 7.0 T, IonSpec Co., Ltd., Lake Forest, CA, USA).

#### 3.1.3. General Procedures for the Preparation of **3a**–**3d**

KOH (1.9 g, 34 mmol) was added to a solution of substituted indoles (13.6 mmol) in DMF (20 mL). Then, a solution of I_2_ (3.8 g, 15 mmol) in DMF (20 mL) was added dropwise into the previous solution and stirred at room temperature for 2 h. The reaction was then quenched with ice water (300 mL, containing 4% NH_3_·H_2_O, 1% Na_2_S_2_O_5_) and the mixture was placed in a refrigerator to ensure complete precipitation. The precipitate was filtered, washed with 200 mL ice water, and dried in vacuo to obtain **2a**–**2d**, which were used without further purification in the next step.

Boc_2_O (3.3 mL, 14.2 mmol) and DMAP (0.2 g, 1.4 mmol) were added to a solution of **2a**–**2d** (11.8 mmol) in 20 mL CH_3_CN and stirred at room temperature for 2 h. After completion of the reaction, the mixture was filtered to obtain **3a**–**3d**.

*tert*-Butyl 3-iodo-5-methoxy-1*H*-indole-1-carboxylate (**3a**). Pale solid; mp 114–116 °C; yield 98%; 1H NMR (400 MHz, CDCl_3_) δ 7.99 (s, 1H, Ar-H), 7.70 (s, 1H, Ar-H), 6.97 (dd, *J* = 9.2 Hz, 2.0 Hz, 1H, Ar-H), 6.84 (d, *J* = 2.0 Hz, 1H, Ar-H), 3.89 (s, 3H, CH_3_), and 1.66 (s, 9H, C(CH_3_)_3_). ^13^C NMR (100 MHz, CDCl_3_) δ 156.5, 148.7, 133.0, 130.6, 129.4, 116.0, 114.5, 103.6, 84.2, 65.2, 55.8, and 28.2.

*tert*-Butyl 5-fluoro-3-iodo-1*H*-indole-1-carboxylate (**3b**). White solid; mp 76–78 °C; yield 80%; 1H NMR (400 MHz, CDCl_3_) δ 8.09 (s, 1H, Ar-H), 7.75 (s, 1H, Ar-H), 7.14–7.01 (m, 2H, Ar-H), and 1.66 (s, 9H, C(CH_3_)_3_). ^13^C NMR (100 MHz, CDCl_3_) δ 160.9, 158.0, 148.5, 131.6, 116.3, 116.3, 113.3 (d, *J* = 25.1 Hz), 107.2 (d, *J* = 24.9 Hz), 84.6, 64.4 (d, *J* = 4.1 Hz), and 28.14.

*tert*-Butyl 5-chloro-3-iodo-1*H*-indole-1-carboxylate (**3c**). White solid; mp 105–107 °C; yield 84%. Other data are consistent with those in the reference [17].

*tert*-Butyl 3-iodo-1*H*-indole-1-carboxylate (**3d**). Brown oil; yield 99%; ^1^H NMR (400 MHz, CDCl_3_) δ 8.09 (s, 1H, Ar-H), 7.75 (s, 1H, Ar-H), 7.14–7.01 (m, 3H, Ar-H), and 1.66 (s, 9H, C(CH_3_)_3_).

#### 3.1.4. General Procedures for the Preparation of **5a**–**5d**

Et_3_N (4 mL, 80 mmol) and pinacolborane (1.8 mL, 12 mmol) was added to a solution of **3a**–**3d** (8 mmol) and Pd(PPh_3_)_4_ (0.28 g, 0.24 mmol) in 1,4-dioxane (50 mL), under argon, and the reaction mixture was stirred at 80 °C for 3 h. After the completion of the reaction, it was cooled to room temperature. Then, 2-iodo-5-bromopyrimidine (1.1 g, 4 mmol), Cs_2_CO_3_ (6.5 g, 20 mmol), and CH_3_OH (40 mL) were added under argon and the reaction mixture was stirred at 100 °C overnight. After the completion of the reaction, the solvents were removed under reduced pressure. The residue was purified by column chromatography on silica gel with CH_2_Cl_2/_CH_3_OH to yield compounds **5a**–**5d**.

3,3′-(Pyrimidine-2,5-diyl)bis(5-methoxy-1*H*-indole) (**5a**). Yellow solid; mp 267–269 °C; yield 90%; ^1^H NMR (400 MHz, DMSO-*d*_6_) δ 11.55 (s, 1H, NH), 11.45 (s, 1H, NH), 9.12 (s, 2H, Ar-H), 8.19 (d, *J* = 2.8 Hz, 1H, Ar-H), 8.12 (d, *J* = 2.4 Hz, 1H, Ar-H), 7.88 (d, *J* = 2.4 Hz, 1H, Ar-H), 7.40 (d, *J* = 3.6 Hz, 1H, Ar-H), 7.38 (d, *J* = 3.6 Hz, 2H, Ar-H), 6.85 (d, *J* = 8.8 Hz, 2H, Ar-H), and 3.84 (s, 6H, CH_3_). ^13^C NMR (100 MHz, DMSO-*d*_6_) δ 161.1, 154.8, 154.7, 154.2, 132.6, 132.4, 129.5, 126.6, 125.5, 125.5, 125.1, 115.1, 113.3, 113.0, 112.6, 112.3, 109.8, 104.4, 101.1, and 55.9. HRMS (ESI) calcd. For C_22_H_19_N_4_O_2_ [M+H]^+^ 371.1503, found 371.1498.

3,3′-(Pyrimidine-2,5-diyl)bis(5-fluoro-1*H*-indole) (**5b**). Yellow solid; mp 288–289 °C; yield 28%; ^1^H NMR (400 MHz, DMSO-*d*_6_) δ 11.80(s, 1H, NH), 11.73 (s, 1H, NH), 9.13 (s, 2H, Ar-H), 8.36–8.22 (m, 2H, Ar-H), 8.03 (d, *J* = 2.8 Hz, 1H, Ar-H), 7.73 (dd, *J* = 10.4, 2.4 Hz, 1H, Ar-H), 7.57–7.44 (m, 2H, Ar-H), and 7.07 (dd, *J* = 12.4, 5.6 Hz, 2H, Ar-H). ^13^C NMR (100 MHz, DMSO-*d*_6_) δ 160.8, 159.4 (d, *J* = 10.3 Hz), 157.1 (d, *J* = 10.5 Hz), 154.3, 134.0, 134.2, 130.8, 126.6, 126.4 (d, *J* = 11.0 Hz), 124.9–125.3 (m, 2C), 115.4 (d, *J* = 4.5 Hz), 113.2–113.6 (m, 2C), 110.7 (d, *J* = 2.9 Hz), 110.4 (d, *J* = 2.9 Hz), 110.2 (d, *J* = 4.7 Hz), 107.1 (d, *J* = 24.7 Hz), and 104.6 (d, *J* = 24.1 Hz). HRMS (ESI) calcd. For C_20_H_13_F_2_N_4_ [M+H]^+^ 347.1103, found 347.1103.

3,3′-(Pyrimidine-2,5-diyl)bis(5-chloro-1*H*-indole) (**5c**). Yellow solid; mp 220–221 °C; yield 79%; ^1^H NMR (400 MHz, DMSO-*d*_6_) δ 11.91 (s, 1H, NH), 11.84 (s, 1H, NH), 9.17 (s, 2H, Ar-H), 8.66 (d, *J* = 2.0 Hz, 1H, Ar-H), 8.33 (d, *J* = 2.8 Hz, 1H, Ar-H), 8.04 (d, *J* = 2.4 Hz, 1H, Ar-H), 8.00 (d, *J* = 1.6 Hz, 1H, Ar-H), 7.55 (d, *J* = 2.4 Hz, 1H, Ar-H), 7.53 (d, *J* = 2.4 Hz, 1H, Ar-H), 7.24 (s, 1H, Ar-H), and 7.22 (s, 1H, Ar-H). ^13^C NMR (100 MHz, DMSO-*d*_6_) δ 160.8, 154.6, 136.0, 135.8, 130.6, 127.1, 126.4, 126.2, 125.6, 125.3, 125.2, 122.4, 122.4, 121.5, 118.9, 115.0, 114.1, 114.1, and 109.8. HRMS (ESI) calcd. For C_20_H_13_C_l2_N_4_ [M+H]^+^ 379.0512, found 379.0510.

3,3′-(Pyrimidine-2,5-diyl)bis(1*H*-indole) (**5d**). Light yellow solid; mp > 300 °C; yield 51%; ^1^H NMR (400 MHz, DMSO-*d*_6_) δ 11.68 (s, 1H, NH), 11.61 (s, 1H, NH), 9.14 (s, 2H, Ar-H), 8.61 (d, *J* = 7.6 Hz, 1H, Ar-H), 8.25 (d, *J* = 2.0 Hz, 1H, Ar-H), 8.00–7.90 (m, 2H, Ar-H), 7.54–7.45 (m, 2H, Ar-H), and 7.25–7.11 (m, 4H, Ar-H). ^13^C NMR (100 MHz, DMSO-*d*_6_) δ 160.6, 153.9, 137.1, 136.9, 128.6, 125.5, 125.1, 124.7, 124.1, 121.9, 121.8, 120.3, 120.1, 119.0, 114.9, 112.1, 111.9, and 109.4. HRMS (ESI) calcd. For C_20_H_15_N_4_ [M+H]^+^ 311.1291, found 311.1292.

#### 3.1.5. Procedures for the Preparation of 3,3′-(Pyrimidine-2,5-diyl)bis(1*H*-indol-5-ol) (Hyrtinadine A, **5e**)

BBr_3_ (2.8 mL, 30 mmol) was added dropwise to a solution of **5a** (0.93 g, 2.5 mmol) in dry CH_2_Cl_2_ (50 mL) at −78 °C under argon and stirred at room temperature for 20 h. After the completion of the reaction, the mixture was cooled to 0 °C. Then, water (20 mL) and a saturated potassium carbonate solution (200 mL) were added, and the mixture was filtered. The residue was purified by column chromatography on silica gel with CH_2_Cl_2_/CH_3_OH to yield compound **5e** (0.4 g, 48% yield). Mp 296–297 °C; ^1^H NMR (400 MHz, DMSO-*d*_6_) δ 11.42 (s, 1H, NH), 11.33 (s, 1H, NH), 9.00 (s, 2H, Ar-H), 8.88 (s, 2H, OH), 8.13 (d, *J* = 2.4 Hz, 1H, Ar-H), 7.99 (s, 1H, Ar-H), 7.82 (d, *J* = 2.4 Hz, 1H, Ar-H), 7.31 (d, *J* = 8.4 Hz, 1H, Ar-H), 7.28 (d, *J* = 8.8 Hz, 1H, Ar-H), 7.22 (s, 1H, Ar-H), and 6.76–6.67 (m, 2H, Ar-H). ^13^C NMR (100 MHz, DMSO-*d*_6_) δ 161.1, 154.0, 152.3, 152.1, 131.9, 131.8, 129.2, 126.9, 125.9, 125.5, 124.7, 114.6, 113.1, 112.7, 112.6, 112.5, 109.0, 106.7, and 103.1. HRMS (ESI) calcd. For C_20_H_15_N_4_O_2_ [M+H]^+^ 343.1190, found 343.1190.

#### 3.1.6. Procedures for the Preparation **5f** and **5g**

Compounds **5f** and **5g** were obtained by following similar procedures for the preparation of compounds **5a**–**5d**.

*tert*-Butyl 3-(5-bromopyrimidin-2-yl)-5-methoxy-1*H*-indole-1-carboxylate(**5f**). White solid; mp 167–168 °C; yield 65%; ^1^H NMR (400 MHz, CDCl_3_) δ 8.80 (s, 2H, Ar-H), 8.48 (s, 1H, Ar-H), 8.13–8.11 (m, 2H, Ar-H), 7.00 (dd, *J* = 8.8, 2.4 Hz, 1H, Ar-H), 3.93 (s, 3H, CH_3_), and 1.69 (s, 9H, C(CH_3_)_3_). ^13^C NMR (100 MHz, CDCl_3_) δ 161.1, 157.5, 156.6, 149.2, 131.0, 129.8, 128.7, 118.6, 116.4, 115.9, 113.8, 105.1, 84.4, 77.4, 77.0, 76.7, 55.8, and 28.2. HRMS (ESI) calcd. For C_18_H_19_BrN_3_O_3_ [M+H]^+^ 404.0604, found 404.0606.

*tert*-Butyl 3-(2-cyanopyrimidin-5-yl)-5-methoxy-1*H*-indole-1-carboxylate (**5g**). Yellow solid; mp 121–122 °C; yield 77%; ^1^H NMR (400 MHz, CDCl_3_) δ 9.11 (s, 2H, Ar-H), 8.14 (d, *J* = 8.4 Hz, 1H, Ar-H), 7.90 (s, 1H, Ar-H), 7.15 (s, 1H, Ar-H), 7.06 (d, *J* = 9.2 Hz, 1H, Ar-H), 3.87 (s, 3H, O-CH_3_), and 1.71 (s, 9H, C(CH_3_)_3_). ^13^C NMR (100 MHz, CDCl_3_) δ 156.9, 155.6, 149.0, 142.6, 131.0, 130.6, 127.9, 125.9, 116.8, 115.9, 114.4, 113.2, 101.5, 85.2, 55.8, and 28.2. HRMS (ESI) calcd. For C_19_H_19_N_4_O_3_ [M+H]^+^ 351.1452, found 351.1450.

#### 3.1.7. General Procedures for the Preparation of **6a**–**6e**

Et_3_N (19 mL,134 mmol) and substituted phenylborate ester (0.74 mmol) were added to a solution of **5f** (0.2 g, 0.5 mmol), Pd(PPh_3_)_4_ (0.06 g, 0. 05 mmol), and Cs_2_CO_3_ (0.4 g, 1.25 mmol) in 1,4-dioxane (10 mL) under argon and the reaction mixture was stirred at 100 °C for 3 h. After the completion of the reaction, the solution was cooled to room temperature and the solvents were removed under reduced pressure. The residue was purified by column chromatography on silica gel with petroleum ether/ethyl acetate to yield compounds **6a**–**6e**.

*tert*-Butyl 5-methoxy-3-(5-phenylpyrimidin-2-yl)-1*H*-indole-1-carboxylate (**6a**). White solid; mp 176–177 °C; yield 95%; ^1^H NMR (400 MHz, CDCl_3_) δ 9.01 (s, 2H, Ar-H), 8.53 (s, 1H, Ar-H), 8.27 (d, *J* = 2.4 Hz, 1H, Ar-H), 8.15 (d, *J* = 9.2 Hz, 1H, Ar-H), 7.68–7.58 (m, 2H, Ar-H), 7.53 (t, *J* = 7.2 Hz, 2H, Ar-H), 7.45 (t, *J* = 7.2 Hz, 1H, Ar-H), 7.01 (dd, *J* = 9.2, 2.4 Hz, 1H, Ar-H), 3.96 (s, 3H, OCH_3_), and 1.70 (s, 9H, C(CH_3_)_3_). ^13^C NMR (100 MHz, CDCl_3_) δ 161.8, 156.6, 154.9, 149.4, 134.8, 131.1, 130.6, 129.4, 129.3, 129.0, 128.6, 126.7, 119.3, 115.8, 113.8, 105.3, 84.3, 55.8, and 28.2. HRMS (ESI) calcd. For C_24_H_24_N_3_O_3_ [M+H]^+^ 402.1812, found 402.1820.

*tert*-Butyl 5-methoxy-3-(5-(2-nitrophenyl)pyrimidin-2-yl)-1*H*-indole-1-carboxylate (**6b**). Yellow solid; mp 72–73 °C; yield 73%; ^1^H NMR (400 MHz, CDCl_3_) δ 8.75 (s, 2H, Ar-H), 8.56 (s, 1H, Ar-H), 8.24 (s, 1H, Ar-H), 8.14 (d, *J* = 8.8 Hz, 1H, Ar-H), 8.09 (d, *J* = 8.0 Hz, 1H, Ar-H), 7.74 (t, *J* = 7.6 Hz, 1H, Ar-H), 7.62 (t, *J* = 7.6 Hz, 1H, Ar-H), 7.48 (d, *J* = 7.6 Hz, 1H, Ar-H), 7.01 (d, *J* = 9.2 Hz, 1H, Ar-H), 3.95 (s, 3H, O-CH_3_), and 1.70 (s, 9H, C(CH_3_)_3_). ^13^C NMR (100 MHz, CDCl_3_) δ 162.4, 156.6, 155.5, 149.3, 148.6, 133.3, 132.2, 131.1, 130.4, 130.0, 129.7, 128.9, 128.2, 125.1, 119.1, 115.9, 113.9, 105.2, 84.4, 55.8, and 28.2. HRMS (ESI) calcd. For C_24_H_23_N_4_O_5_ [M+H]^+^ 447.1663, found 447.1668.

*tert*-Butyl 5-methoxy-3-(5-(3-nitrophenyl)pyrimidin-2-yl)-1*H*-indole-1-carboxylate (**6c**). Yellow solid; mp 182–183 °C; yield 54%; ^1^H NMR (400 MHz, CDCl_3_) δ 9.04 (s, 2H, Ar-H), 8.56 (s, 1H, Ar-H), 8.50 (t, *J* = 2 Hz, 1H, Ar-H), 8.30 (dd, *J* = 8.4, 1.2 Hz, 1H, Ar-H), 8.24 (d, *J* = 2.8 Hz, 1H, Ar-H), 8.13 (d, *J* = 9.2 Hz, 1H, Ar-H), 7.95 (d, *J* = 8 Hz, 1H, Ar-H), 7.72 (t, *J* = 8.0 Hz, 1H, Ar-H), 7.02 (dd, *J* = 8.8, 2.4 Hz, 1H, Ar-H), 3.96 (s, 3H, O-CH_3_), and 1.71 (s, 9H, C(CH_3_)_3_). ^13^C NMR (100 MHz, CDCl_3_) δ 162.8, 156.6, 154.9, 149.3, 149.0, 136.6, 132.3, 131.1, 130.5, 130.0, 128.9, 128.2, 123.2, 121.4, 119.0, 115.9, 113.9, 105.2, 84.5, 55.8, and 28.2. HRMS (ESI) calcd. For C_24_H_23_N_4_O_5_ [M+H]^+^ 447.1663, found 447.1666.

*tert*-Butyl 5-methoxy-3-(5-(2-(trifluoromethyl)phenyl)pyrimidin-2-yl)-1*H*-indole-1-carboxylate (**6d**). White solid; mp 70–72 °C; yield 43%; ^1^H NMR (400 MHz, CDCl_3_) δ 8.76 (s, 2H, Ar-H), 8.56 (s, 1H, Ar-H), 8.27 (s, 1H, Ar-H), 8.16 (d, *J* = 8.8 Hz, 1H, Ar-H), 7.84 (d, *J* = 8.0 Hz, 1H, Ar-H), 7.67 (t, *J* = 7.6 Hz, 1H, Ar-H), 7.59 (t, *J* = 7.6 Hz, 1H, Ar-H), 7.39 (d, *J* = 7.6 Hz, 1H, Ar-H), 7.02 (d, *J* = 9.2 Hz, 1H, Ar-H), 3.95 (s, 3H, O-CH_3_), and 1.70 (s, 9H, C(CH_3_)_3_). ^13^C NMR (100 MHz, CDCl_3_) δ 162.3, 156.6, 156.1, 149.3, 134.4, 132.2, 132.0, 131.2, 129.9, 129.7, 129.0, 128.8, 126.6, 119.2, 115.9, 113.9, 105.3, 84.3, 55.8, and 28.2. HRMS (ESI) calcd. For C_25_H_23_F_3_N_3_O_3_ [M+H]^+^ 470.1686, found 470.1691.

*tert*-Butyl 3-(5-(2-aminophenyl)pyrimidin-2-yl)-5-methoxy-1*H*-indole-1-carboxylate (**6e**). Yellow solid; mp 170–171 °C; yield 87%; ^1^H NMR (400 MHz, CDCl_3_) δ 8.92 (s, 1H, Ar-H), 8.53 (s, 1H, Ar-H), 8.26 (d, *J* = 2.4 Hz, 1H, Ar-H), 8.15 (d, *J* = 9.2 Hz, 1H, Ar-H), 7.24 (d, *J* = 8.0 Hz, 1H, Ar-H), 7.16 (d, *J* = 7.2 Hz, 1H, Ar-H), 7.01 (dd, *J* = 9.2, 2.6 Hz, 1H, Ar-H), 6.90 (t, *J* = 7.2 Hz, 1H, Ar-H), 6.83 (d, *J* = 8.0 Hz, 1H, Ar-H), 3.95 (s, 3H, Ome), 3.79 (s, 2H, NH_2_), and 1.70 (s, 9H, C(CH_3_)_3_). ^13^C NMR (100 MHz, CDCl_3_) δ 161.7, 156.9, 156.6, 149.3, 144.1, 131.1, 130.5, 129.9, 129.5, 129.4, 129.0, 120.6, 119.3, 116.2, 115.9, 113.8, 105.2, 84.3, 55.8, and 28.2. HRMS (ESI) calcd. For C_24_H_25_N_4_O_3_ [M+H]^+^ 417.1921, found 417.1928.

#### 3.1.8. General Procedures for the Preparation of **7a**–**7f**

Acyl chloride (0.7 mmol) and Et_3_N (0.18 mL, 1.2 mmol) were added to a solution of **6e** (0.25 g, 0.6 mmol) in dry CH_2_Cl_2_ (10 mL) and stirred at room temperature for 3 h. After completion of the reaction, a saturated sodium carbonate aqueous solution (20 mL) was added, and the mixture was extracted with CH_2_Cl_2_ (30 mL × 3). The combined organic phase was washed with brine (60 mL), dried over anhydrous Na_2_SO_4,_ and concentrated. The residue was purified by column chromatography on silica gel with petroleum ether/ethyl acetate to yield compounds **7a**–**7f**.

*tert*-Butyl 3-(5-(2-benzamidophenyl)pyrimidin-2-yl)-5-methoxy-1*H*-indole-1-carboxylate (**7a**). Yellow solid; mp 153–155 °C; yield 65%; ^1^H NMR (400 MHz, CDCl_3_) δ 8.86 (s, 2H Ar-H), 8.50 (s, 1H Ar-H), 8.20 (d, *J* = 2.4 Hz, 1H Ar-H), 8.18–8.10 (m, 2H Ar-H), 7.80 (s, 1H NH), 7.72 (d, *J* = 7.2 Hz, 2H Ar-H), 7.54–7.45 (m, 2H Ar-H), 7.40 (t, *J* = 7.6 Hz, 2H Ar-H), 7.35 (d, *J* = 3.6 Hz, 2H Ar-H), 7.00 (dd, *J* = 9.2, 2.4 Hz, 1H Ar-H) 3.91 (s, 3H OCH_3_), and 1.69 (s, 9H C(CH_3_)_3_). ^13^C NMR (100 MHz, CDCl_3_) δ 165.9, 162.3, 156.8, 156.6, 149.3, 135.0, 134.2, 132.1, 131.1, 130.4, 129.8, 128.9, 128.5, 128.0, 127.0, 126.0, 124.4, 119.1, 115.9, 113.8, 105.2, 84.4, 55.8, and 28.2. HRMS (ESI) calcd. For C_31_H_29_N_4_O_4_ [M+H]^+^ 521.2183, found521.2185.

*tert*-Butyl 3-(5-(2-(4-fluorobenzamido)phenyl)pyrimidin-2-yl)-5-methoxy-1*H*-indole-1-carboxylate (**7b**). Light yellow solid; mp 169–170 °C; yield 94%; ^1^H NMR (400 MHz, CDCl_3_) δ 8.83 (s, 2H Ar-H), 8.48 (s, 1H Ar-H), 8.18 (s, 1H Ar-H), 8.12 (d, *J* = 9.2 Hz, 1H Ar-H), 8.07 (d, *J* = 8.0 Hz, 1H Ar-H), 7.79 (s, 1H NH), 7.77–7.69 (m, 2H Ar-H), 7.53–7.45 (m, 1H Ar-H), 7.35 (d, *J* = 3.6 Hz, 2H Ar-H), 7.06 (t, *J* = 8.0 Hz, 2H Ar-H), 7.00 (d, *J* = 9.2 Hz, 1H Ar-H), 3.90 (s, 3H OCH_3_), and 1.69 (s, 9H C(CH_3_)_3_). ^13^C NMR (100 MHz, CDCl_3_) δ 166.3, 164.9, 162.3, 156.7, 156.6, 149.3, 134.8, 131.1, 130.3(d, *J* = 2 Hz), 129.8, 129.5, 129.5, 128.9, 128.5, 128.3, 126.3, 124.7, 119.0, 116.1, 115.9, 113.7, 105.3, 84.5, 55.8, and 28.2. HRMS (ESI) calcd. For C_31_H_28_FN_4_O_4_ [M+H]^+^ 539.2089, found 539.2096.

*tert*-Butyl 5-methoxy-3-(5-(2-(4-methylbenzamido)phenyl)pyrimidin-2-yl)-1*H*-indole-1-carboxylate (**7c**). White solid; mp 159–160 °C; yield 53%; ^1^H NMR (400 MHz, CDCl_3_) δ 8.87 (s, 2H Ar-H), 8.52 (s, 1H Ar-H), 8.22–8.18 (m, 2H Ar-H), 8.13 (d, *J* = 9.2 Hz, 1H Ar-H), 7.73 (s, 1H NH), 7.61 (d, *J* = 8.0 Hz, 2H Ar-H), 7.54–7.48 (m, 1H Ar-H), 7.37–7.32 (m, 2H Ar-H), 7.20 (d, *J* = 8.0 Hz, 2H Ar-H), 7.01 (dd, *J* = 9.2, 2.8 Hz, 1H Ar-H), 3.92 (s, 3H OCH_3_), 2.36 (s, 3H CH_3_), and 1.69 (s, 9H C(CH_3_)_3_). ^13^C NMR (100 MHz, CDCl_3_) δ 165.7, 162.3, 156.8, 156.6, 149.3, 142.7, 135.1, 131.3, 130.4, 129.8, 129.8, 129.6, 129.6, 128.9, 128.5, 127.7, 127.0, 125.8, 124.2, 119.1, 115.9, 113.8, 105.2, 84.4, 55.8, 29.7, and 28.2. HRMS (ESI) calcd. For C_32_H_31_N_4_O_4_ [M+H]^+^ 535.2340, found 535.2346.

*tert*-Butyl 5-methoxy-3-(5-(2-pivalamidophenyl)pyrimidin-2-yl)-1*H*-indole-1-carboxylate (**7d**). Light yellow solid; mp 101–102 °C; yield 80%; ^1^H NMR (400 MHz, CDCl_3_) δ 8.81 (s, 2H Ar-H), 8.55 (s, 1H Ar-H), 8.24 (d, *J* = 2.8 Hz, 1H Ar-H), 8.14 (d, *J* = 9.2 Hz, 1H Ar-H), 8.05 (d, *J* = 8.0 Hz, 1H Ar-H), 7.49–7.43 (m, 1H Ar-H), 7.33–7.29 (m, 2H Ar-H), 7.27 (s, 1H NH), 7.02 (dd, *J* = 9.1, 2.8 Hz, 1H Ar-H), 3.94 (s, 3H OCH_3_), 1.71 (s, 9H C(CH_3_)_3_), and 1.19 (s, 9H C(CH_3_)_3_). ^13^C NMR (100 MHz, CDCl_3_) δ 176.8, 162.2, 156.8, 156.6, 149.3, 135.1, 131.1, 130.2, 129.7, 129.7, 129.0, 128.6, 128.2, 125.8, 124.9, 119.2, 115.9, 113.7, 105.3, 84.4, 55.8, 39.7, 28.2, and 27.5. HRMS (ESI) calcd. For C_29_H_33_N_4_O_4_ [M+H]^+^ 501.2496, found 501.2502.

*tert*-Butyl 5-methoxy-3-(5-(2-octanamidophenyl)pyrimidin-2-yl)-1*H*-indole-1-carboxylate (**7e**). White solid; mp 67–68 °C; yield 69%; ^1^H NMR (400 MHz, CDCl_3_) δ 8.79 (s, 2H Ar-H), 8.53 (s, 1H Ar-H), 8.24 (d, *J* = 2.6 Hz, 1H Ar-H), 8.14 (d, *J* = 9.2 Hz, 1H Ar-H), 8.03 (d, *J* = 8.0 Hz, 1H Ar-H), 7.48–7.42 (m, 1H Ar-H), 7.30 (d, *J* = 3.8 Hz, 2H), 7.07 (s, 1H, NH), 7.01 (dd, *J* = 9.1, 2.6 Hz, 1H Ar-H), 3.94 (s, 3H OCH_3_), 2.26 (t, *J* = 7.5 Hz, 2H CH_2_), 1.70 (s, 9H C(CH_3_)_3_), 1.67–1.54 (m, 2H CH_2_), 1.33–1.11 (m, 8H (CH_2_)_4_), and 0.82 (t, *J* = 6.8 Hz, 3H CH_3_). ^13^C NMR (100 MHz, CDCl_3_) δ 172.1, 161.8, 156.6, 156.5, 149.3, 134.9, 131.0, 130.1, 129.6, 129.5, 128.9, 128.8, 128.5, 125.9, 125.1, 119.1, 115.7, 113.6, 105.4, 84.3, 55.7, 37.2, 31.6, 29.2, 29.0, 28.2, 25.6, 22.6, and 14.0. HRMS (ESI) calcd. For C_32_H_39_N_4_O_4_ [M+H]^+^ 543.2966, found 543.2974.

*tert*-Butyl 3-(5-(2-(3-(difluoromethyl)-1-methyl-1*H*-pyrazole-4-carboxamido)phenyl)pyrimidin-2-yl)-5-methoxy-1*H*-indole-1-carboxylate (**7f**). White solid; mp 178–179 °C; yield 72%; ^1^H NMR (400 MHz, CDCl_3_) δ 8.80 (s, 2H Ar-H), 8.52 (s, 1H Ar-H), 8.23 (d, *J* = 2.0 Hz, 1H Ar-H), 8.18–8.08 (m, 2H Ar-H), 7.92 (s, 2H Ar-H), 7.54–7.44 (m, 1H Ar-H), 7.33 (d, *J* = 4.0 Hz, 2H Ar-H), 7.05–6.96 (m, 1H Ar-H), 6.65 (t, *J* = 54.4 Hz, 1H Ar-H), 3.93 (s, 3H OCH_3_), 3.86 (s, 3H CH_3_), and 1.69 (s, 9H C(CH_3_)_3_). ^13^C NMR (100 MHz, CDCl_3_) δ 162.1, 159.6, 156.8, 156.6, 149.3, 136.0, 135.0, 131.1, 130.5, 129.7, 129.0, 128.6, 128.3, 125.9, 124.3, 119.3, 116.4, 115.8, 113.7, 111.6, 105.4, 84.3, 55.8, 39.5, and 28.2. HRMS (ESI) calcd. For C_30_H_29_F_2_N_6_O_4_ [M+H]^+^ 575.2213, found 575.2223.

#### 3.1.9. General Procedures for the Preparation of Compounds **8a** and **8b**

The solution of **7a** or **7d** (0.88 mmol) in CF_3_COOH (20 mL) was stirred at room temperature for 3 h. After completion of the reaction, most of the solvent was evaporated in vacuo. Then, a saturated sodium carbonate aqueous solution (40 mL) was added, and the mixture was extracted with CH_2_Cl_2_ (30 mL × 3). The combined organic phase was washed with brine (60 mL), dried over anhydrous Na_2_SO_4,_ and concentrated. The residue was purified by column chromatography on silica gel with CH_2_Cl_2_/CH_3_OH to yield compounds **8a** and **8b**.

*N*-(2-(2-(5-Methoxy-1*H*-indol-3-yl)pyrimidin-5-yl)phenyl)benzamide (**8a**). Yellow solid; mp 235–236 °C; yield 95%; ^1^H NMR (400 MHz, DMSO-*d*_6_) δ 11.61 (s, 1H NH), 10.19 (s, 1H NH), 8.80 (s, 2H Ar-H), 8.17 (d, *J* = 2.8 Hz, 1H Ar-H), 8.01 (d, *J* = 2.4 Hz, 1H Ar-H), 7.84 (d, *J* = 7.2 Hz, 2H Ar-H), 7.61–7.42 (m, 7H Ar-H), 7.35 (d, *J* = 8.8 Hz, 1H Ar-H), 6.83 (dd, *J* = 8.8, 2.4 Hz, 1H Ar-H), and 3.79 (s, 3H OCH_3_). ^13^C NMR (100 MHz, DMSO-*d*_6_) δ 165.7, 162.2, 155.8, 154.4, 135.4, 134.1, 132.5, 132.1, 131.6, 130.0, 129.8, 128.8, 128.4, 128.2, 127.9, 127.5, 127.1, 126.1, 114.2, 112.6, 111.8, 103.7, and 55.3. HRMS (ESI) calcd. For C_26_H_21_N_4_O_2_ [M+H]^+^ 421.1659, found 421.1657.

*N*-(2-(2-(5-Methoxy-1*H*-indol-3-yl)pyrimidin-5-yl)phenyl)pivalamide (**8b**). Brown solid; mp 206–207 °C; yield 92%; ^1^H NMR (400 MHz, DMSO-*d*_6_) δ 11.61 (s, 1H NH), 9.19 (s, 1H NH), 8.72 (s, 2H Ar-H), 8.21 (d, *J* = 2.1 Hz, 1H Ar-H), 8.06 (s, 1H Ar-H), 7.52 (d, *J* = 7.2 Hz, 1H Ar-H), 7.48–7.30 (m, 4H Ar-H), 6.85 (d, *J* = 8.6 Hz, 1H Ar-H), 3.81 (s, 3H OCH_3_), and 1.08 (s, 9H C(CH_3_)_3_). ^13^C NMR (100 MHz, DMSO-*d*_6_) δ 176.4, 162.1, 156.0, 154.4, 135.8, 132.9, 132.1, 129.7, 129.6, 128.6, 128.5, 127.6, 126.8, 126.1, 114.3, 112.6, 111.8, 103.7, 55.3, and 27.1. HRMS (ESI) calcd. For C_24_H_25_N_4_O_2_ [M+H]^+^ 401.1972, found 401.1980.

#### 3.1.10. Procedures for the Preparation of Compound 5-(5-Methoxy-1*H*-indol-3-yl)pyrimidine-2-carbohydrazide (**8c**)

Compound **5g** (0.5 g, 1.4 mmol) was added to a solution of NaOH (0.6 g, 150 mmol) in water (30 mL) and the mixture was stirred at 100 °C for 2 h. After completion of the reaction, the mixture was cooled to room temperature. The pH of the mixture was adjusted to 3 with dilute hydrochloric acid. Then, the mixture was filtered, and the residue was recrystallized with CH_3_OH/CH_3_OCH_3_. The filter residue was dissolved in CH_3_OH (5 mL) and thionyl chloride (1 mL, 14 mmol) was added to the solution at 0 °C and stirred at 70 °C for 3 h. After completion of the reaction, most of the solvent was evaporated in vacuo. The residue was added to ethanol (15 mL) and the solution was added dropwise to 80% hydrazine hydrate (0.2 mL, 3.2 mmol) and stirred at refluxed temperature for 5 h. After completion of the reaction, the solvent was evaporated in vacuo to yield **8c**. Yellow solid; mp 244–245 °C; yield 76%; ^1^H NMR (400 MHz, DMSO-*d*_6_) δ 11.80 (s, 1H NH), 10.03 (s, 1H NH), 9.26 (s, 2H Ar-H), 8.08 (s, 1H Ar-H), 7.43 (s, 1H Ar-H), 7.40 (d, *J* = 5.6 Hz, 1H Ar-H), 6.87 (d, *J* = 8.4 Hz, 1H Ar-H), 4.80 (s, 2H NH_2_), and 3.83 (s, 3H CH_3_).

#### 3.1.11. General Procedures for the Preparation of **9a**–**9g**

Aldehyde (0.8 mmol) was added to a solution of **8c** (0.2 g, 0.7 mmol) in ethanol (20 mL) and stirred at refluxed temperature for 3 h. After completion of the reaction, the solvent was evaporated in vacuo. The residue was recrystallized with ether to yield compounds **9a**–**9g**.

(*E*)-*N*′-Benzylidene-5-(5-methoxy-1*H*-indol-3-yl)pyrimidine-2-carbohydrazide (**9a**). Red solid; mp 253–255 °C; yield 69%; ^1^H NMR (400 MHz, DMSO-*d*_6_) δ 12.29 (s, 1H NH), 11.89 (s, 1H CH), 9.35 (s, 2H Ar-H), 8.66 (s, 1H Ar-H), 8.15 (s, 1H Ar-H), 7.75 (m, 2H Ar-H), 7.64–7.33 (m, 5H), 6.89 (d, *J* = 7.6 Hz, 1H), and 3.85 (s, 3H). ^13^C NMR (100 MHz, DMSO-*d*_6_) δ 158.9, 154.6, 153.6, 153.3, 149.0, 134.3, 132.0, 131.7, 130.2, 128.9, 127.2, 126.9, 124.8, 113.2, 112.4, 107.8, 100.7, and 55.4. HRMS (ESI) calcd. for C_21_H_18_N_5_O_2_ [M+H]^+^ 372.1455, found 372.1452.

(*E*)-*N*′-(2-Fluorobenzylidene)-5-(5-methoxy-1*H*-indol-3-yl)pyrimidine-2-carbohydrazide (**9b**). Red solid; mp 255–256 °C; yield 78%; ^1^H NMR (400 MHz, DMSO-*d*_6_) δ 12.53 (s, 1H NH), 11.93 (s, 1H CH), 9.36 (s, 2H Ar-H), 8.93 (s, 1H Ar-H), 8.16 (d, *J* = 2.4 Hz, 1H Ar-H), 8.00 (t, *J* = 7.2 Hz, 1H Ar-H), 7.55–7.50 (m, 1H NH), 7.44 (d, *J* = 9.2 Hz, 2H Ar-H), 7.37–7.28 (m, 2H Ar-H), 6.89 (dd, *J* = 8.8, 2.0 Hz, 1H Ar-H), and 3.85 (s, 3H CH_3_). ^13^C NMR (100 MHz, DMSO-*d*_6_) δ 162.1, 159.6, 154.6, 153.2, 142.4, 132.3 (d, *J* = 8.6 Hz), 132.0, 126.9, 126.6, 124. 9, 124.7, 121.6 (d, *J* = 9.7 Hz), 115.9 (d, *J* = 20.7 Hz), 113.3, 112.3, 107.7, 100.7, and 55.4. HRMS (ESI) calcd. for C_21_H_17_FN_5_O_2_ [M+H]^+^ 390.1361, found 390.1368.

(*E*)-5-(5-Methoxy-1*H*-indol-3-yl)-*N*′-(2-methoxybenzylidene)pyrimidine-2-carbohydrazide (**9c**). Brown solid; mp 274–275 °C; yield 63%; ^1^H NMR (400 MHz, DMSO-*d*_6_) δ 12.38 (s, 1H NH), 12.05 (s, 1H CH), 9.35 (s, 2H Ar-H), 8.99 (s, 1H Ar-H), 8.16 (s, 1H Ar-H), 7.91 (d, *J* = 7.8 Hz, 1H Ar-H), 7.46–7.43 (m, 3H Ar-H), 7.13 (d, *J* = 8.4 Hz, 1H Ar-H), 7.05 (t, *J* = 7.2 Hz, 1H Ar-H), 6.89 (d, *J* = 7.2 Hz, 1H Ar-H) 3.88 (s, 3H OCH_3_), and 3.85 (s, 3H OCH_3_). ^13^C NMR (100 MHz, DMSO-*d*_6_) δ 159.3, 158.0, 154.6, 153.2, 145.4, 132.0, 131.7, 126.9, 125.8, 124.7, 122.0, 120.8, 113.3, 112.3, 111.9, 107.7, 100.7, 55.8, and 55.4. HRMS (ESI) calcd. for C_22_H_20_N_5_O_3_ [M+H]^+^ 402.1561, found 402.1563.

(*E*)-*N*′-(2,2-Dimethylpropylidene)-5-(5-methoxy-1*H*-indol-3-yl)pyrimidine-2-carbohydrazide (**9d**). Yellow solid; mp 176–177 °C; yield 30%; ^1^H NMR (400 MHz, DMSO-*d*_6_) δ 11.74 (s, 1H NH), 11.70 (s, 1H CH), 9.30 (s, 2H Ar-H), 8.11 (d, *J* = 2.8 Hz, 1H Ar-H), 7.86 (s, 1H Ar-H), 7.44–7.39 (m, 2H Ar-H), 6.88 (dd, *J* = 8.8, 2.0 Hz, 1H Ar-H), 3.84 (s, 3H CH_3_), and 1.11 (s, 9H C(CH_3_)_3_). ^13^C NMR (100 MHz, DMSO-*d*_6_) δ 160.6, 158.7, 154.6, 153.8, 153.3, 132.0, 131.4, 126.7, 124.7, 113.2, 112.4, 107.9, 100.6, 55.4, 34.6, and 27.1. HRMS (ESI) calcd. for C_19_H_22_N_5_O_2_ [M+H]^+^ 352.1768, found 352.1770.

(*E*)-5-(5-Methoxy-1*H*-indol-3-yl)-*N*’-(pyridin-4-ylmethylene)pyrimidine-2-carbohydrazide (**9e**). Yellow solid; mp 203–204 °C; yield 65%; ^1^H NMR (400 MHz, DMSO-*d*_6_) δ 12.87 (s, 1H NH), 11.91 (s, 1H CH), 9.38 (s, 2H Ar-H), 8.86 (d, *J* = 4.8 Hz, 2H Ar-H), 8.75 (s, 1H Ar-H), 8.17 (d, *J* = 2.0 Hz, 1H Ar-H), 8.07 (d, *J* = 5.2 Hz, 2H Ar-H), 7.48–7.37 (m, 2H Ar-H), 6.89 (d, *J* = 8.8 Hz, 1H Ar-H), and 3.85 (s, 3H). ^13^C NMR (100 MHz, DMSO-*d*_6_) δ 159.5, 154.7, 153.3, 152.8, 146.7, 145.4, 144.7, 132.0, 127.0, 124.7, 122.8, 113.3, 112.4, 107.8, 100.7, and 55.4. HRMS (ESI) calcd. for C_20_H_17_N_6_O_2_ [M+H]^+^ 373.1408, found 373.1408.

(*E*)-5-(5-Methoxy-1*H*-indol-3-yl)-*N*′-(thiophen-3-ylmethylene)pyrimidine-2-carbohydrazide (**9f**). Brown solid; mp 283–284 °C; yield 36%; ^1^H NMR (400 MHz, DMSO-*d*_6_) δ 12.19 (s, 1H NH), 11.86 (s, 1H CH), 9.35 (s, 2H Ar-H), 8.67 (s, 1H Ar-H), 8.14 (d, *J* = 2.4 Hz, 1H Ar-H), 7.95 (d, *J* = 2.0 Hz, 1H Ar-H), 7.68–7.66 (m, 1H NH), 7.52 (d, *J* = 4.8 Hz, 1H Ar-H), 7.44 (d, *J* = 8.8 Hz, 2H), 6.89 (dd, *J* = 8.8, 2.0 Hz, 1H), and 3.85 (s, 3H). ^13^C NMR (100 MHz, DMSO-*d*_6_) δ 158.9, 154.6, 153.7, 153.3, 144.5, 137.5, 132.0, 131.6, 128.5, 127.7, 126.8, 124.8, 124.7, 113.2, 112.4, 107.9, 100.7, and 55.4. HRMS (ESI) calcd. for C_19_H_16_N_5_O_2_S [M+H]^+^ 378.1019, found 378.1020.

(*E*)-5-(5-Methoxy-1*H*-indol-3-yl)-*N*′-(naphthalen-2-ylmethylene)pyrimidine-2-carbohydrazide (**9g**). Red solid; mp 272–273 °C; yield 27%; ^1^H NMR (400 MHz, DMSO-*d*_6_) δ 12.41 (s, 1H NH), 11.85 (s, 1H CH), 9.38 (s, 2H Ar-H), 8.83 (s, 1H Ar-H), 8.16 (s, 2H Ar-H), 8.16–7.98 (m, 4H Ar-H), 7.59 (s, 2H Ar-H), 7.45 (s, 2H Ar-H), 6.90 (d, *J* = 7.4 Hz, 1H Ar-H), and 3.86 (s, 3H CH_3_). ^13^C NMR (100 MHz, DMSO-*d*_6_) δ 159.5, 155.2, 154.1, 153.8, 149.5, 134.3, 133.3, 132.5, 132.1, 129.5, 129.0, 128.9, 128.3, 127.7, 127.3, 127.3, 125.3, 123.2, 113.7, 113.0, 108.4, 101.2, and 55.9. HRMS (ESI) calcd. for C_25_H_20_N_5_O_2_ [M+H]^+^ 422.1612, found 422.1614.

### 3.2. Biological Assay

The activities of all synthesized natural products and derivatives were tested on representative test organisms. To ensure the reliability of data, each bioassay was replicated at least three times. The activity results were evaluated according to a percentage of mortalities from 0 to 100 (0 means no activity and 100 means total kill or inhibition).

#### 3.2.1. Antiviral Biological Assay

The antiviral activities against TMV were carried out using previously reported methods [20,21]. The detailed procedures can be seen in the Supplementary Materials.

#### 3.2.2. Antifungal Biological Assay

The procedures of antifungal activity tests were described in the literature [20,21]. The detailed procedures can also be seen in the Supplementary Materials.

## 4. Conclusions

In summary, the marine natural product hyrtinadine A and its derivatives were synthesized, and their antiviral and fungicidal activities were evaluated systematically for the first time. Compounds **5a**, **5c**, **8a**, **9e**, and **9f** exhibited excellent in vivo anti-TMV activities at 500 μg/mL which were similar to that of ningnanmycin. The research on the structure-activity relationship showed that the substituent group in the 5-position of the indole of hyrtinadine A influences the anti-TMV activity, and that methoxy and chlorine are the most prominent. In addition, the bis-indole skeleton is important to the activity, but it is not unchangeable. Moreover, the structurally simplified compounds **8a**, **9e**, and **9f** with high antiviral activities were successfully discovered and all the synthesized compounds were found to display broad-spectrum fungicidal activities. The current research results thus provide reliable support to develop hyrtinadine A and its analogs as novel antiviral and fungicidal agents.

## Figures and Tables

**Table 1:** In vivo antiviral activities of ningnanmycin, ribavirin, and compounds **5a**–**5d**, **6a**–**6e**, **7a**–**7f**, **8a**–**8b**, and **9a**–**9g** against TMV.

Compound	Conc. (µg/mL)	Inhibition Rate (%) *^a^*		
		Inactivation Effect	Curative Effect	Protection Effect
**5a**	**500**	**53 ± 1**	**48 ± 2**	**55 ± 1**
	**100**	**21 ± 2**	**14 ± 1**	**20 ± 2**
**5b**	500	25 ± 1	/	/
**5c**	**500**	**51 ± 2**	**54 ± 4**	**47 ± 2**
	**100**	**18 ± 1**	**22 ± 2**	**15 ± 1**
**5d**	500	43 ± 4	47 ± 4	51 ± 3
	100	10 ± 1	9 ± 2	15 ± 1
**5e**	500	11 ± 1	/	/
**5f**	500	34 ± 2	/	/
**5g**	500	43 ± 3	47 ± 4	48 ± 3
	100	14 ± 1	12 ± 1	18 ± 2
**6a**	500	18 ± 1	/	/
**6b**	500	28 ± 3	/	/
**6c**	500	16 ± 1	/	/
**6e**	500	40 ± 3	45 ± 1	36 ± 4
	100	9 ± 2	15 ± 1	8 ± 1
**7a**	500	36 ± 1	/	/
**7b**	500	46 ± 1	38 ± 2	40 ± 1
	100	8 ± 4	13 ± 1	16 ± 1
**7c**	500	44 ± 1	48 ± 4	39 ± 4
	100	12 ± 2	17 ± 2	7 ± 1
**7d**	500	43 ± 2	46 ± 4	38 ± 4
	100	13 ± 1	9 ± 1	15 ± 1
**7e**	500	33 ± 2	/	/
**7f**	500	40 ± 1	35 ± 4	47 ± 3
	100	15 ± 1	6 ± 2	12 ± 1
**8a**	**500**	**55 ± 3**	**52 ± 3**	**57 ± 2**
	**100**	**19 ± 1**	**22 ± 1**	**26 ± 1**
**8b**	500	39 ± 1	/	/
**9a**	500	42 ± 3	43 ± 2	37 ± 4
	100	16 ± 1	10 ± 2	12 ± 1
**9b**	500	45 ± 2	39 ± 3	47 ± 4
	100	18 ± 1	10 ± 2	16 ± 1
**9c**	500	3 ± 1	/	/
**9d**	500	42 ± 1	33 ± 5	37 ± 2
	100	9 ± 1	13 ± 1	10 ± 3
**9e**	**500**	**51 ± 1**	**45 ± 4**	**54 ± 3**
	**100**	**24 ± 2**	**18 ± 1**	**23 ± 1**
**9f**	**500**	**55 ± 2**	**51 ± 1**	**55 ± 3**
	**100**	**22 ± 1**	**18 ± 2**	**19 ± 1**
**9g**	500	21 ± 2	/	/
Ningnanmycin	500	58 ± 1	55 ± 2	57 ± 1
	100	26 ± 1	24 ± 1	28 ± 1
Ribavirin	500	39 ± 1	38 ± 2	40 ± 1
	100	12 ± 1	13 ± 1	14 ± 2

*^a^* Average of three replicates; all results are expressed as the mean ± SD; activity data with prominent were presented in bold.

**Table 2:** Fungicidal activities of carbendazim, chlorothalonil, and compounds **5a**–**5d**, **6a**–**6e**, **7a**–**7f**, **8a**–**8b**, and **9a**–**9g** against 14 kinds of fungi ^a^.

Compound	Fungicidal Activity %/50 μg/mL													
	F.C	C.H	P.P	R.C	B.M	W.A	F.M	A.S	F.G	P.I	P.C	S.S	R.S	B.C
**5a**	32 ± 1	38 ± 3	63 ± 2	56 ± 2	26 ± 1	24 ± 1	42 ± 2	29 ± 1	21 ± 1	6 ± 1	19 ± 2	47 ± 3	27 ± 2	33 ± 1
**5b**	24 ± 2	29 ± 2	61 ± 1	49 ± 1	29 ± 2	30 ± 3	33 ± 1	50 ± 1	57 ± 2	11 ± 1	48 ± 2	60 ± 2	18 ± 1	59 ± 2
**5c**	32 ± 3	33 ± 2	56 ± 2	70 ± 1	23 ± 1	36 ± 2	25 ± 1	50 ± 2	21 ± 1	17 ± 2	62 ± 1	47 ± 1	18 ± 2	7 ± 1
**5d**	29 ± 1	25 ± 1	49 ± 1	51 ± 2	29 ± 2	24 ± 1	42 ± 3	21 ± 1	7 ± 2	11 ± 1	48 ± 2	47 ± 3	46 ± 1	46 ± 3
**5e**	29 ± 2	29 ± 2	66 ± 2	56 ± 1	34 ± 1	30 ± 2	33 ± 1	36 ± 2	21 ± 1	11 ± 2	33 ± 1	27 ± 1	27 ± 1	20 ± 1
**5f**	32 ± 1	25 ± 1	56 ± 1	60 ± 3	26 ± 2	30 ± 1	33 ± 3	36 ± 3	21 ± 1	22 ± 1	48 ± 3	20 ± 1	27 ± 2	13 ± 1
**5g**	42 ± 3	46 ± 2	75 ± 2	71 ± 3	49 ± 1	52 ± 3	42 ± 1	36 ± 2	14 ± 2	11 ± 1	48 ± 1	20 ± 2	27 ± 1	26 ± 2
**6a**	32 ± 1	54 ± 2	69 ± 1	70 ± 1	34 ± 2	42 ± 1	54 ± 3	14 ± 1	14 ± 3	6 ± 1	10 ± 1	60 ± 1	64 ± 3	7 ± 1
**6b**	32 ± 2	33 ± 1	64 ± 2	60 ± 2	23 ± 1	36 ± 2	33 ± 1	36 ± 2	57 ± 1	11 ± 1	33 ± 2	27 ± 2	27 ± 1	13 ± 2
**6c**	24 ± 1	29 ± 2	49 ± 1	49 ± 1	20 ± 2	21 ± 1	33 ± 2	29 ± 1	64 ± 2	22 ± 1	29 ± 1	27 ± 1	18 ± 2	3 ± 1
**6d**	21 ± 2	25 ± 1	54 ± 2	41 ± 2	23 ± 1	24 ± 2	29 ± 1	14 ± 1	7 ± 1	11 ± 1	14 ± 2	47 ± 2	27 ± 1	7 ± 1
**6e**	45 ± 1	50 ± 1	79 ± 1	77 ± 1	46 ± 2	46 ± 1	46 ± 2	14 ± 1	14 ± 1	6 ± 1	19 ± 2	13 ± 1	18 ± 2	13 ± 1
**7a**	18 ± 1	29 ± 2	49 ± 2	56 ± 2	23 ± 1	18 ± 1	29 ± 1	36 ± 2	7 ± 1	11 ± 1	48 ± 2	13 ± 2	46 ± 1	13 ± 1
**7b**	37 ± 1	42 ± 1	66 ± 1	54 ± 1	34 ± 1	42 ± 2	46 ± 2	29 ± 1	14 ± 2	11 ± 1	38 ± 1	20 ± 1	18 ± 2	40 ± 2
**7c**	21 ± 2	21 ± 2	49 ± 3	37 ± 3	20 ± 2	21 ± 1	29 ± 1	21 ± 2	7 ± 1	6 ± 1	24 ± 2	13 ± 1	46 ± 1	20 ± 1
**7d**	26 ± 1	38 ± 1	49 ± 1	60 ± 1	23 ± 1	27 ± 2	33 ± 2	29 ± 1	29 ± 2	17 ± 1	33 ± 1	40 ± 2	36 ± 3	40 ± 2
**7e**	21 ± 1	33 ± 3	48 ± 2	56 ± 2	26 ± 1	18 ± 1	25 ± 1	21 ± 2	43 ± 1	6 ± 1	24 ± 2	13 ± 1	27 ± 1	13 ± 1
**7f**	24 ± 2	25 ± 1	46 ± 3	27 ± 1	20 ± 2	21 ± 2	33 ± 2	14 ± 1	7 ± 1	6 ± 1	24 ± 2	7 ± 1	27 ± 2	46 ± 3
**8a**	32 ± 1	42 ± 2	61 ± 1	66 ± 3	34 ± 1	27 ± 1	38 ± 1	21 ± 2	14 ± 2	11 ± 2	33 ± 1	13 ± 1	18 ± 1	20 ± 1
**8b**	32 ± 2	13 ± 1	64 ± 3	33 ± 1	26 ± 2	21 ± 2	21 ± 1	21 ± 2	21 ± 1	6 ± 1	38 ± 2	13 ± 1	46 ± 3	26 ± 2
**9a**	18 ± 1	29 ± 2	41 ± 1	34 ± 2	23 ± 1	27 ± 1	25 ± 2	43 ± 1	57 ± 2	33 ± 1	33 ± 1	27 ± 2	46 ± 1	53 ± 2
**9b**	37 ± 2	33 ± 1	51 ± 2	34 ± 1	11 ± 1	30 ± 1	33 ± 3	21 ± 2	7 ± 1	6 ± 1	24 ± 1	13 ± 2	9 ± 1	26 ± 1
**9c**	21 ± 1	46 ± 2	57 ± 3	49 ± 2	23 ± 3	18 ± 1	42 ± 2	29 ± 1	43 ± 2	17 ± 2	38 ± 3	27 ± 1	9 ± 2	46 ± 2
**9d**	24 ± 1	21 ± 1	53 ± 1	34 ± 1	20 ± 1	24 ± 2	33 ± 1	43 ± 3	14 ± 1	17 ± 1	24 ± 1	7 ± 1	18 ± 1	53 ± 2
**9e**	21 ± 2	54 ± 2	57 ± 3	63 ± 2	14 ± 1	21 ± 1	42 ± 2	36 ± 1	7 ± 1	22 ± 2	10 ± 1	7 ± 1	18 ± 2	46 ± 2
**9f**	32 ± 2	13 ± 1	57 ± 2	34 ± 1	40 ± 2	30 ± 2	25 ± 2	21 ± 2	7 ± 1	6 ± 1	14 ± 1	13 ± 1	18 ± 2	26 ± 1
**9g**	32 ± 1	13 ± 2	59 ± 1	39 ± 2	29 ± 1	42 ± 2	29 ± 1	7 ± 1	7 ± 1	6 ± 1	10 ± 2	7 ± 2	9 ± 1	40 ± 3
Carbendazim ^b^	97 ± 1	29 ± 2	97 ± 1	99 ± 1	97 ± 1	97 ± 1	88 ± 2	21 ± 1	100	100	29 ± 1	93 ± 2	9 ± 1	100
Chlorothalonil ^b^	76 ± 2	54 ± 1	82 ± 1	66 ± 2	69 ± 1	67 ± 3	79 ± 1	43 ± 1	57 ± 2	83 ± 2	95 ± 1	80 ± 1	73 ± 2	88 ± 1

^a^ Average of three replicates; F.C, *Fusarium oxysporum* f. sp. Cucumeris; C.H, *Cercospora arachidicola* Hori; P.P, *Physalospora piricola*; R.C, *Rhizoctonia cerealis*; B.M, *Bipolaris maydis*; W.A, *Watermelon anthracnose*; F.M, *Fusarium moniliforme*; A.S, *Alternaria solani*; F.G, *Fusarium graminearum*; P.I, *Phytophthora infestans*; P.C, *Phytophthora capsici*; S.S, *Sclerotinia sclerotiorum*; R.S, *Rhizoctonia solani*; and B.C, *Botrytis cinerea*; ^b^ The commercial agricultural fungicides chlorothalonil and carbendazim were used as controls for the comparison of antifungal activities.

## Data Availability

All data used to support the findings of this study are included within the article and Supplementary Materials.

## Supplementary Materials

The following supporting information can be downloaded at: https://www.mdpi.com/article/10.3390/molecules27238439/s1, Section S1: Detailed bio-assay procedures for the anti-TMV and fungicidal activities; Section S2: copies of ^1^H & ^13^C NMR spectra (Figures S1–S50). References [25,26,27,28,29,30,31] were cited in supplementary materials.
