# Supplementary material for: Discovery of Hyrtinadine A and Its Derivatives as Novel Antiviral and Anti-Phytopathogenic-Fungus Agents
Source: Molecules. 2022 Dec 2;27(23):8439. doi: 10.3390/molecules27238439 (PMC9738573; doi:10.3390/molecules27238439)
Supplement: Supplementary file 1 [file molecules-27-08439-s001.zip › molecules-2052695-supplementary.pdf]

Supplementary Materials

Discovery of Hyrtinadine A and Its Derivatives as Novel Antiviral and Anti-phytopathogenic-fungus Agents

Ji Dong [1,2,3], Henan Ma [3], Beibei Wang [3], Shaoxiang Yang [2], Ziwen Wang [1,*], Yongqiang Li [3,*], Yuxiu Liu [3] and Qingmin Wang [3,*]

[1]Tianjin Key Laboratory of Structure and Performance for Functional Molecules, College of Chemistry, Tianjin Normal University, Tianjin 300387, China;

[2]Beijing Key Laboratory of Flavor Chemistry , Beijing Technology and Business University (BTBU), Beijing 100048, China;

[3]State Key Laboratory of Elemento-Organic Chemistry, Research Institute of Elemento-Organic Chemistry, College of Chemistry, Collaborative Innovation Center of Chemical Science and Engineering (Tianjin), Nankai University, Tianjin 300071, China.

Ziwen Wang, E-mail: hxxywzw@tjnu.edu.cn; Phone: 0086-22-23766531; Fax: 0086-22-23766531;

Yongqiang Li, E-mail: lyq@nankai.edu.cn; Phone: 0086-22-23503792; Fax: 0086-22-23503792;

Qingmin Wang, E-mail: wangqm@nankai.edu.cn; Phone: 0086-22-23503952; Fax: 0086-22-23503952.

Contents

Section S1: The detailed bio-assay procedures S3
Section S2: Copies of NMR spectra (Figures S1–S50) S7
Figures S1–S2. ^{1}H NMR and ^{13}C NMR spectra of 5a S7
Figures S3–S4. ^{1}H NMR and ^{13}C NMR spectra of 5b S8
Figures S5–S6. ^{1}H NMR and ^{13}C NMR spectra of 5c S9
Figures S7–S8. ^{1}H NMR and ^{13}C NMR spectra of 5d S10
Figures S9–S10. ^{1}H NMR and ^{13}C NMR spectra of 5e S11
Figures S11–S12. ^{1}H NMR and ^{13}C NMR spectra of 6a S12
Figures S13–S14. ^{1}H NMR and ^{13}C NMR spectra of 6b S13
Figures S15–S16. ^{1}H NMR and ^{13}C NMR spectra of 6c S14
Figures S17–S18. ^{1}H NMR and ^{13}C NMR spectra of 6d S15
Figures S19–S20. ^{1}H NMR and ^{13}C NMR spectra of 6e S16
Figures S21–S22. ^{1}H NMR and ^{13}C NMR spectra of 7a S17
Figures S23–S24. ^{1}H NMR and ^{13}C NMR spectra of 7b S18
Figures S25–S26. ^{1}H NMR and ^{13}C NMR spectra of 7c S19
Figures S27–S28. ^{1}H NMR and ^{13}C NMR spectra of 7d S20
Figures S29–S30. ^{1}H NMR and ^{13}C NMR spectra of 7e S21
Figures S31–S32. ^{1}H NMR and ^{13}C NMR spectra of 7f S22
Figures S33–S34. ^{1}H NMR and ^{13}C NMR spectra of 8a S23
Figures S35–S36. ^{1}H NMR and ^{13}C NMR spectra of 8b S24
Figures S37–S38. ^{1}H NMR and ^{13}C NMR spectra of 9a S25
Figures S39–S40. ^{1}H NMR and ^{13}C NMR spectra of 9b S26
Figures S41–S42. ^{1}H NMR and ^{13}C NMR spectra of 9c S27
Figures S43–S44. ^{1}H NMR and ^{13}C NMR spectra of 9d S28
Figures S45–S46. ^{1}H NMR and ^{13}C NMR spectra of 9e S29
Figures S47–S48. ^{1}H NMR and ^{13}C NMR spectra of 9f S30
Figures S49–S50. ^{1}H NMR and ^{13}C NMR spectra of 9g S31

Section S1: Detailed bio-assay procedures

Phytotoxic activity. The phytotoxic activity test was carried out in a climate chamber at a temperature of 25 °C. The growing 3–5 leaf stage tobaccos (*Nicotiana tabacum var Xanthi nc*) were selected. The compound solution (100 µg/mL or 500 µg/mL) was sprayed on the leaves and then tested the plant height and weight changes after 0, 3, 7 and 10 days respectively. There are three replicates for each compound.[1]

Antiviral Biological Assay.[2]

Purification of Tobacco Mosaic Virus.

Using Gooding's method[3], the upper leaves of *Nicotiana tabacum* L. inoculated with TMV were selected and ground in phosphate buffer and then filtered through double-layer pledget. The filtrate was centrifuged at 10000 g, treated with PEG twice, and centrifuged again. The whole experiment was processed at 4 °C. Absorbance value was estimated at 260 nm by ultraviolet spectrophotometer.

$$Virus\ concn = (A_{260} \times dilution\ ratio)E\ /_{1cm}^{0.1\%,\ 260nm}$$

Protective Effect of Compounds against TMV in Vivo.

The compound solution was smeared on growing *Nicotiana. tabacum* L. leaves (at least 3 leaves) of the same age. In another pot, the leaves were smeared with the solvent as a control. After 12 h, the leaves were inoculated with TMV with the juice-leaf rubbing method and then washed with water. The total local lesion numbers appearing on the leaves 3−4 days after inoculation were recorded.[4] There are three replicates for each compound.

Inactivation Effect of Compounds against TMV in Vivo.

To test viral inhibition, equal volumes of the virus and the compound solution were mixed together for 30 min. The mixture was then inoculated into the growing *N. tabacum* L leaves of the same age, and another pot was inoculated with the mixture of solvent and the virus as the control. The local lesion numbers were recorded 3−4 days after inoculation.[5] There are three replicates for each compound.

Curative Effect of Compounds against TMV in Vivo.

TMV (concentration of 6.0×10^{-3} μg/mL) was inoculated on the growing leaves of *N. tabacum* L. of the same age. Then, the leaves were washed with water and dried. The compound solution was smeared on the inoculated leaves, while inoculated leaves in another pot were smeared with the solvent as a control. The local lesion numbers were recorded 3−4 days after inoculation.[6] There are three replicates for each compound. The in vitro and in vivo inhibition rates of the compound were then calculated according to the following formula ("av" means average, and controls were not treated with compound).

Inhibition rate (%) = [(av local lesion no. of control − av local lesion no. of drug-treated)/av local lesion no. of control] × 100%

Fungicidal activities[7]

The fungicidal activities of compounds were evaluated in mycelial growth tests conducted in artificial media against 14 plant pathogens at a rate of 50 *μ*g/mL. Each test compound was dissolved in a suitable amount of acetone and diluted with water containing 0.1% TW-80 to a concentration of 500 *μ*g/mL. To each petri dish was

added 1 mL of the test solution and 9 mL of culture medium to make a 50 μg/mL concentration of the test compound, while in another petri dish was added 1 mL distilled water containing 0.1% TW-80 and 9 mL of culture medium as a blank control. A 4 mm diameter of hyphal growth was cut using a hole puncher on a growing fungal S6 culture and the hyphae were moved to the petri dish containing the test compound. Each assay was performed three times. The dishes were stored in controlled environment cabinets (24±1°C) for 4 days, after which the diameter of mycelial growth was measured and the percentage inhibition was calculated using the following equation: Percentage inhibition (%) = (averaged diameter of mycelia in blank controls – averaged diameter of mycelia in medicated tablets) / (averaged diameter of mycelia in blank controls – 4 mm) × 100.

Reference:

(1) Hao, Y.N.; Guo, J.C.; Wang, Z.W.; Liu, Y.X.; Li, Y.Q.; Ma, D.J.; Wang, Q.M. Discovery of tryptanthrins as novel antiviral and anti-phytopathogenic-fungus agents. *J. Agric. Food Chem.* **2020**, *68*, 5586–5595.

(2) Wang, Z.W.; Wei, P.; Wang, L.Z.; Wang, Q.M. Design, synthesis, and anti-tobacco mosaic virus (TMV) activity of phenanthroindolizidines and their analogues. *J. Agric. Food Chem*. **2012**, *60*, 10212−10219.

(3) Gooding, G.V., Jr.; Hebert, T.T. A simple technique for purification of tobacco mosaic virus in large quantities. *Phytopathology* **1967**, 57, 1285−1290.

(4) Li, S.Z.; Wang, D.M.; Jiao, S.M. In *Pesticide Experiment Methods-Fungicide Sector*; Li, S. Z., Ed.; Agriculture Press of China: Beijing, China, **1991**; 93−94.

(5) Leberman, R. Isolation of plant viruses by means of simple coacervates. *Virol* **1966**, *30*, 341–347.

(6) Fraenkel Conrat, H.; Williams, R.C. Reconstitution of active tobacco mosaic virus fromits inactive protein and nucleic acid components. *PNAS* U S A 1955, *41*, 690–698.

(7) Zhao, H.P.; Liu, Y.X.; Cui, Z.P.; Beattie, D.; Gu, Y.C.; Wang, Q.M. Design, synthesis, and biological activities of arylmethylamine substituted chlorotriazine and methylthiotriazine compounds. *J. Agric. Food Chem.* **2011**, *59*, 11711−11717.

Section S2: Copies of NMR spectra (Figures S1–S50)

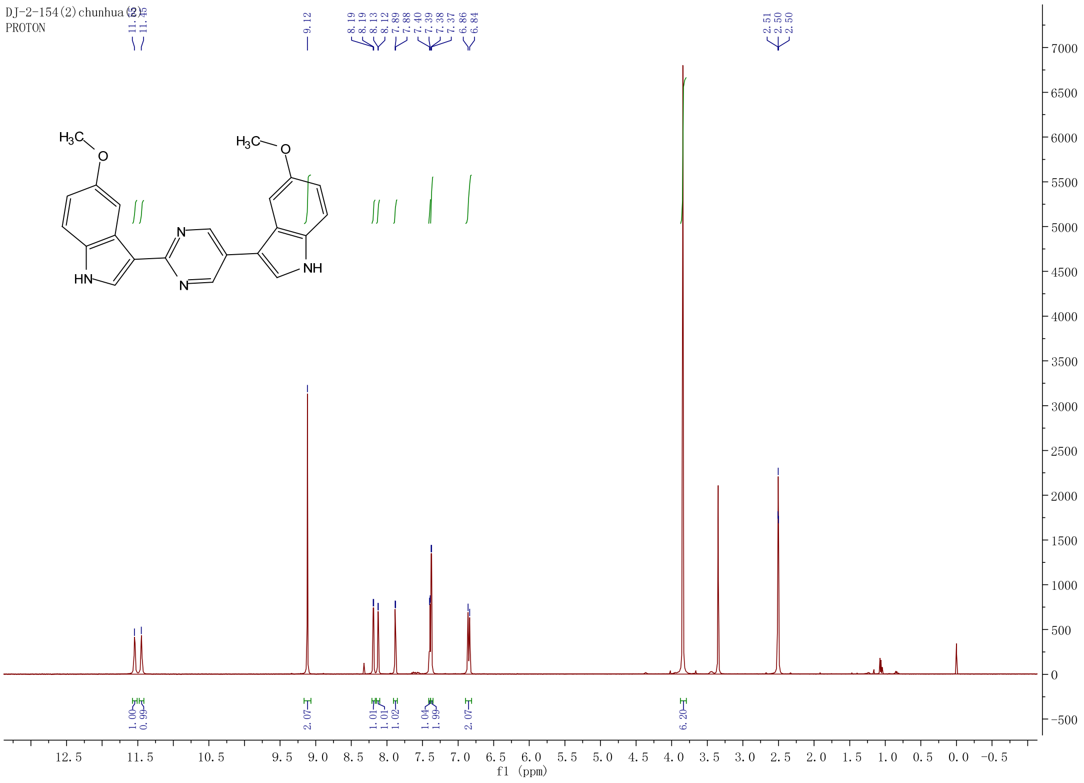

Figure S1. ^{1}H NMR spectrum of 5a

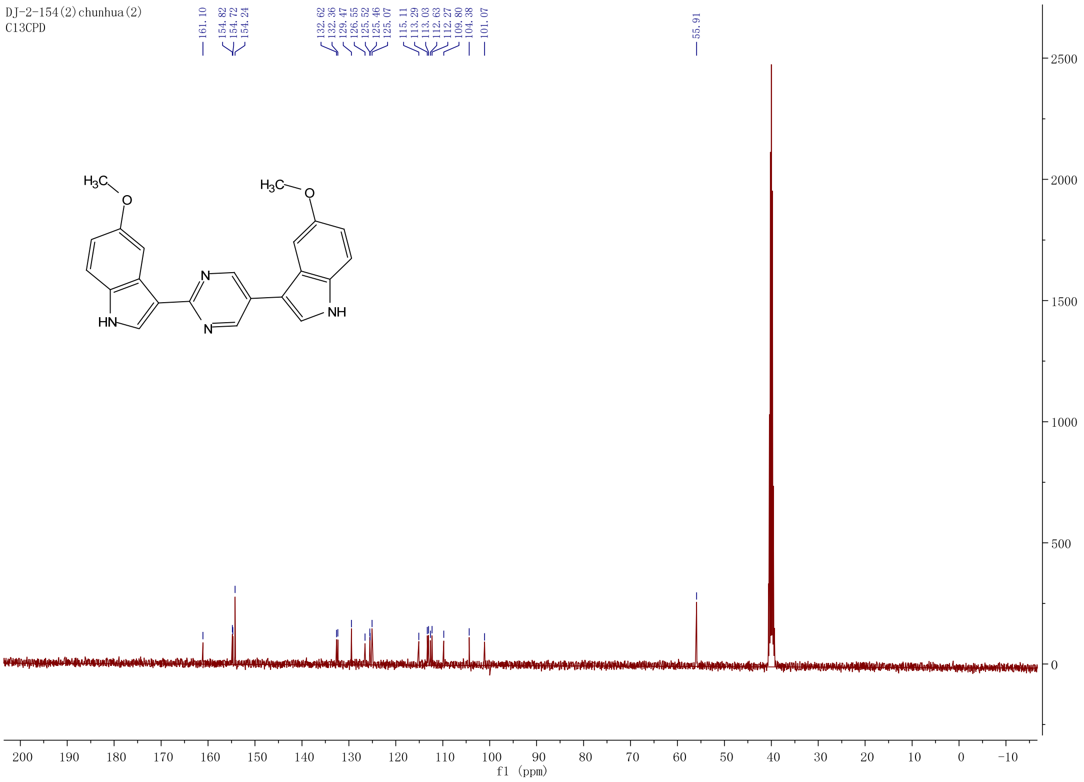

Figure S2. ^{13}C NMR spectrum of 5a

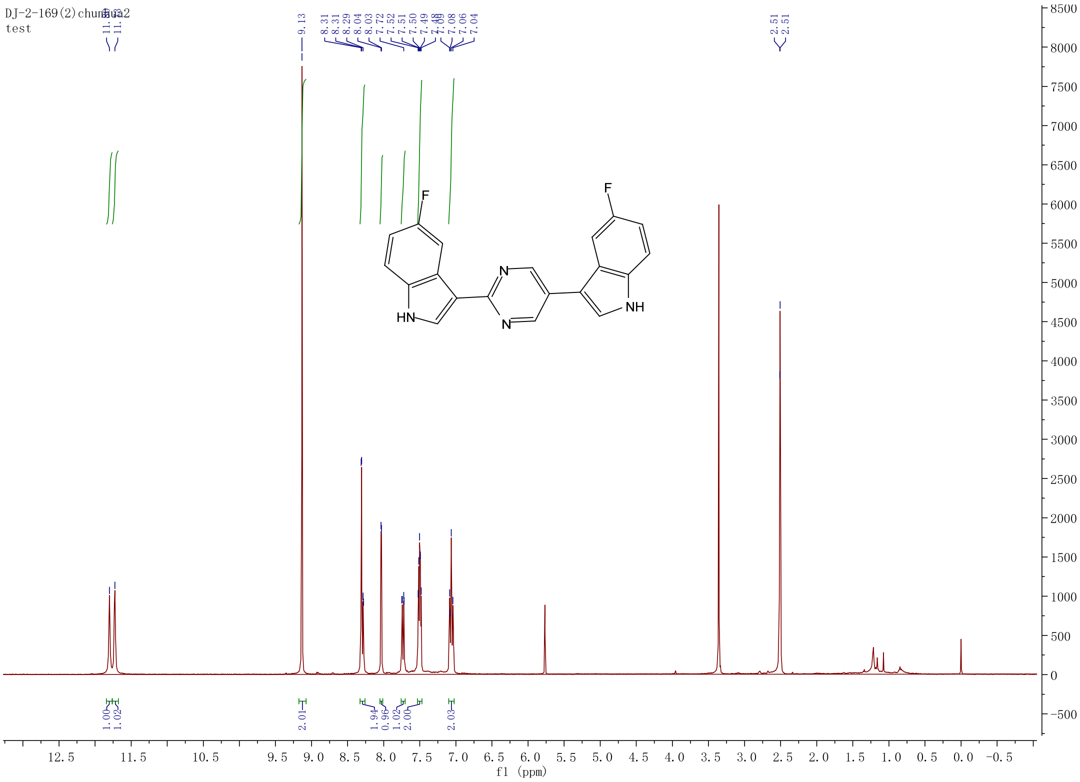

Figure S3. ^{1}H NMR spectrum of 5b

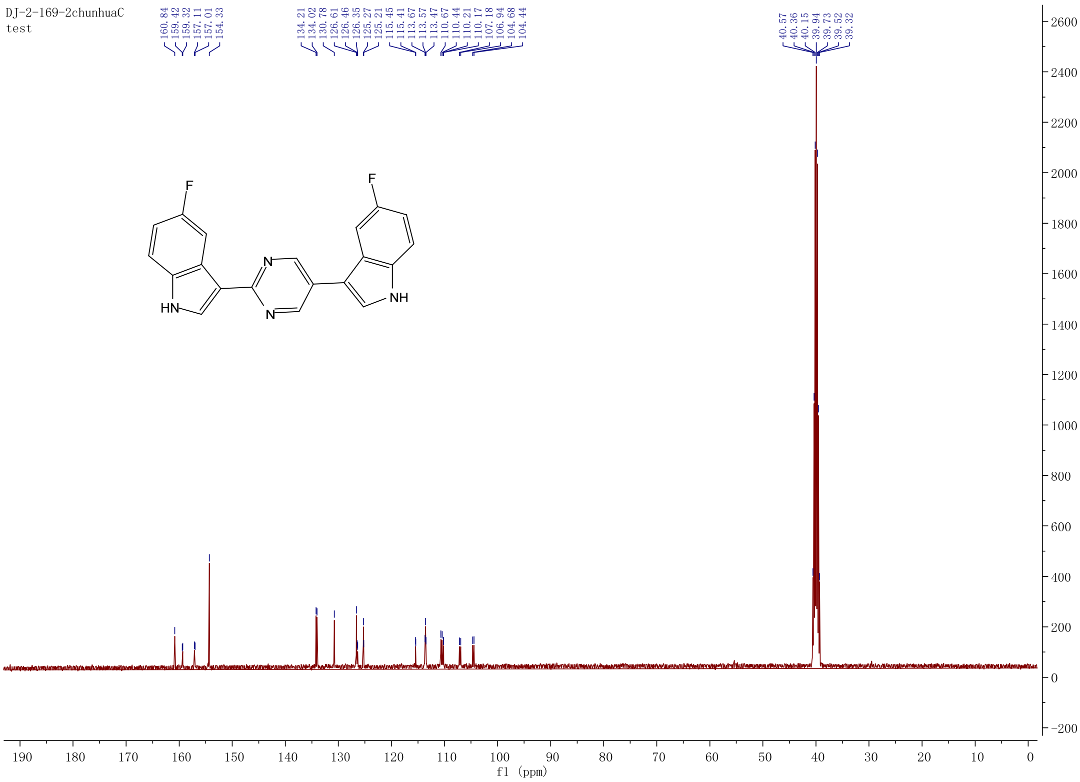

Figure S4. ^{13}C NMR spectrum of 5b

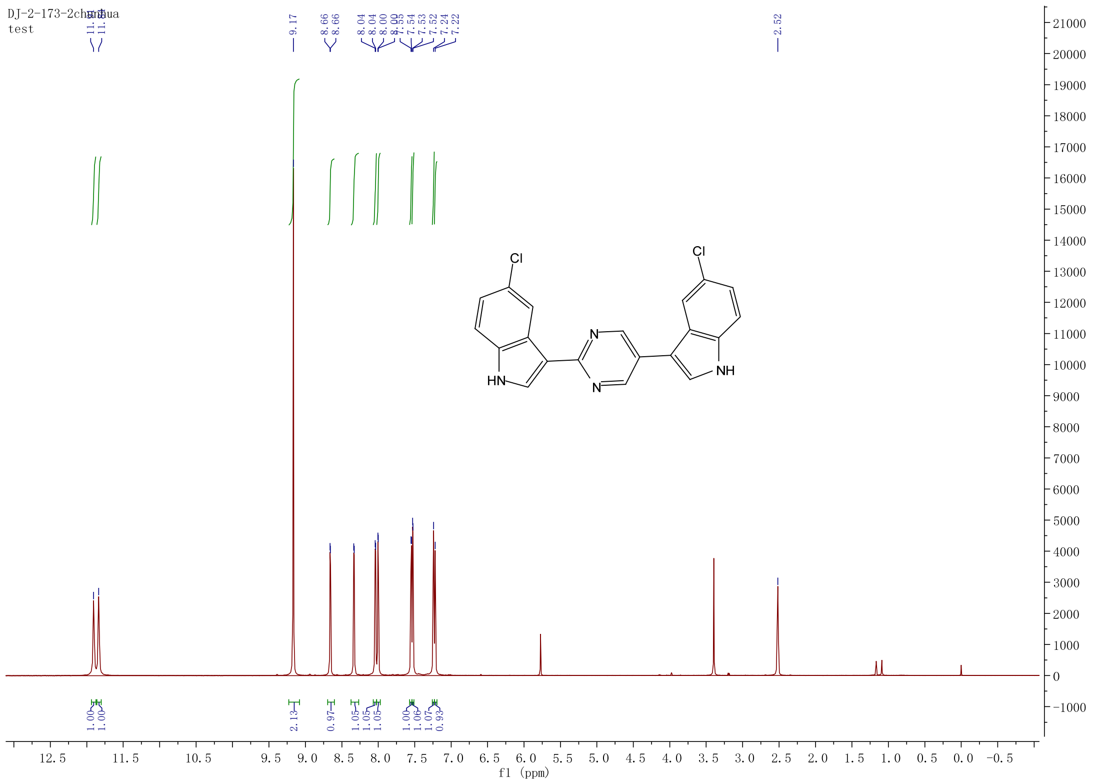

Figure S5. ^{1}H NMR spectrum of 5c

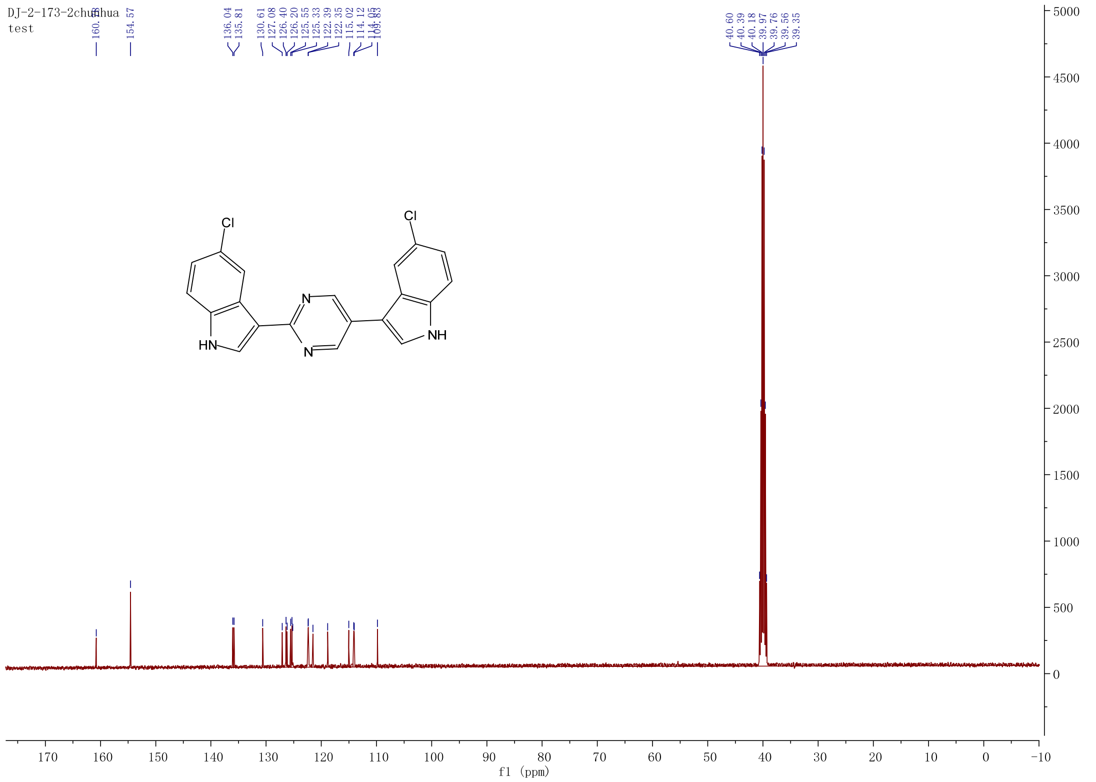

Figure S6. ^{13}C NMR spectrum of 5c

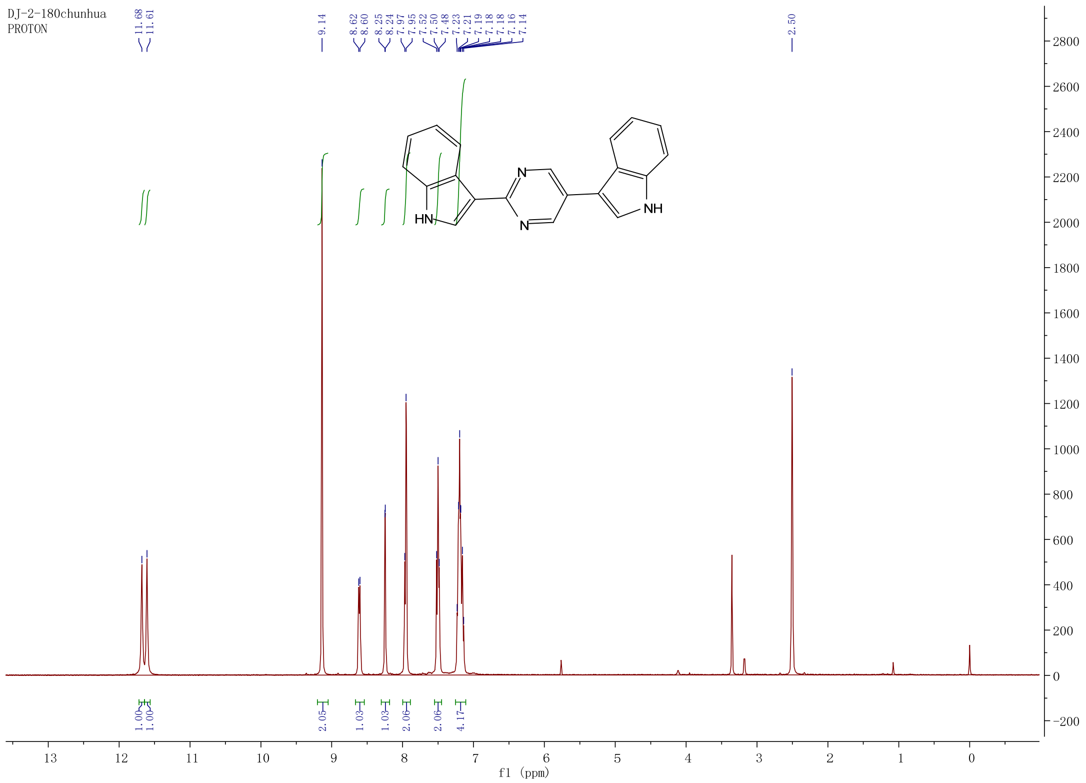

Figure S7. ^{1}H NMR spectrum of 5d

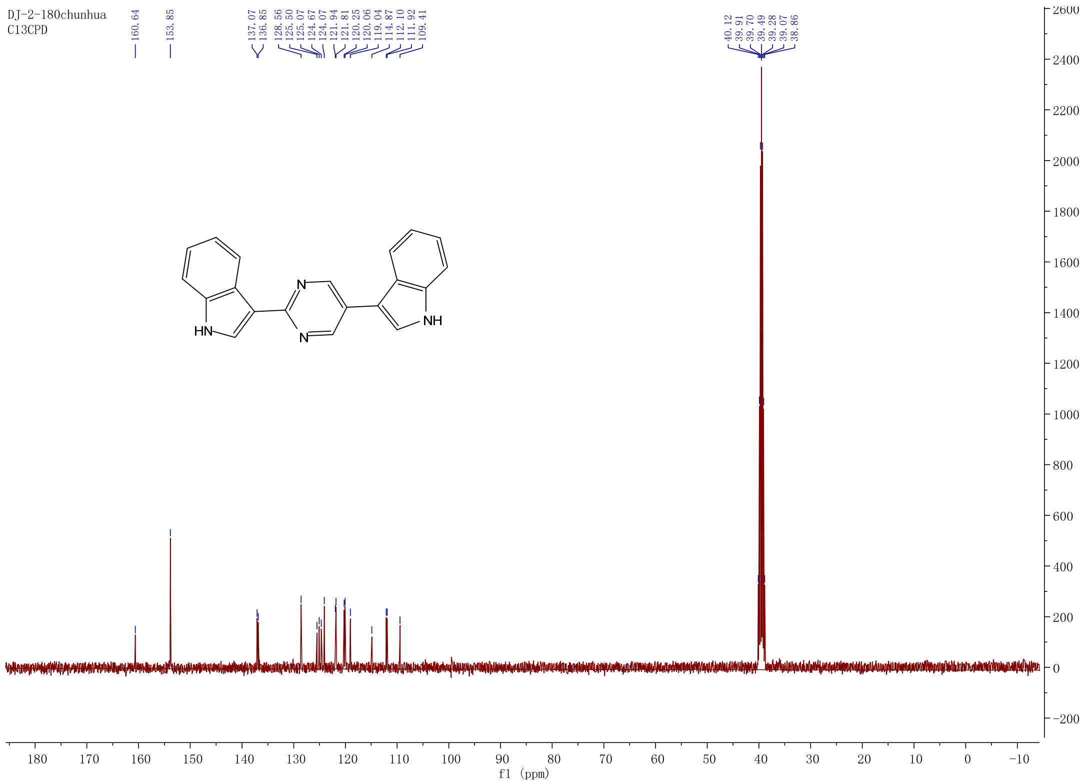

Figure S8. ^{13}C NMR spectrum of 5d

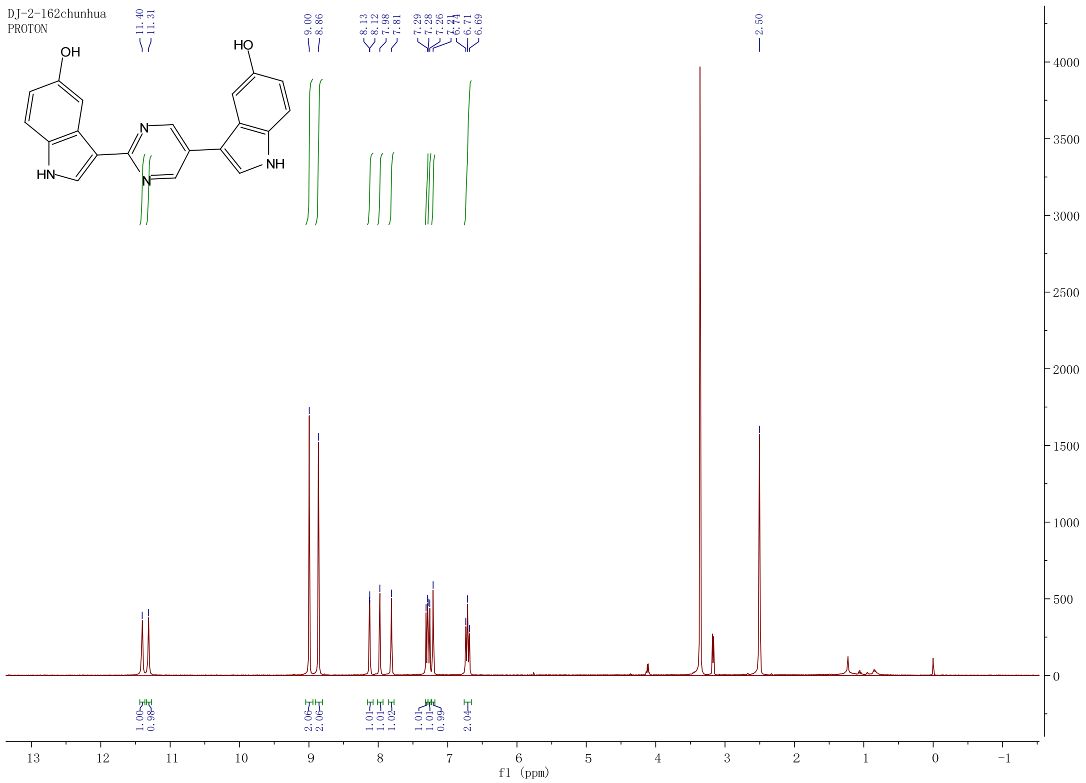

Figure S9. ^{1}H NMR spectrum of 5e

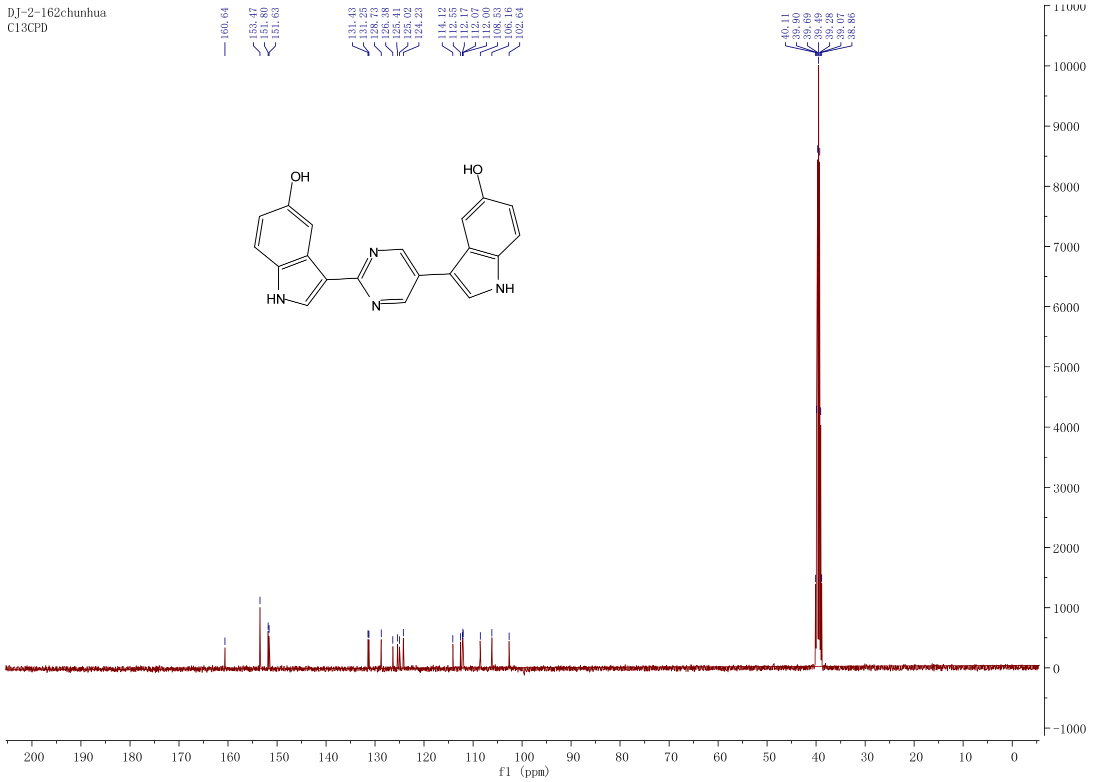

Figure S10. ^{13}C NMR spectrum of 5e

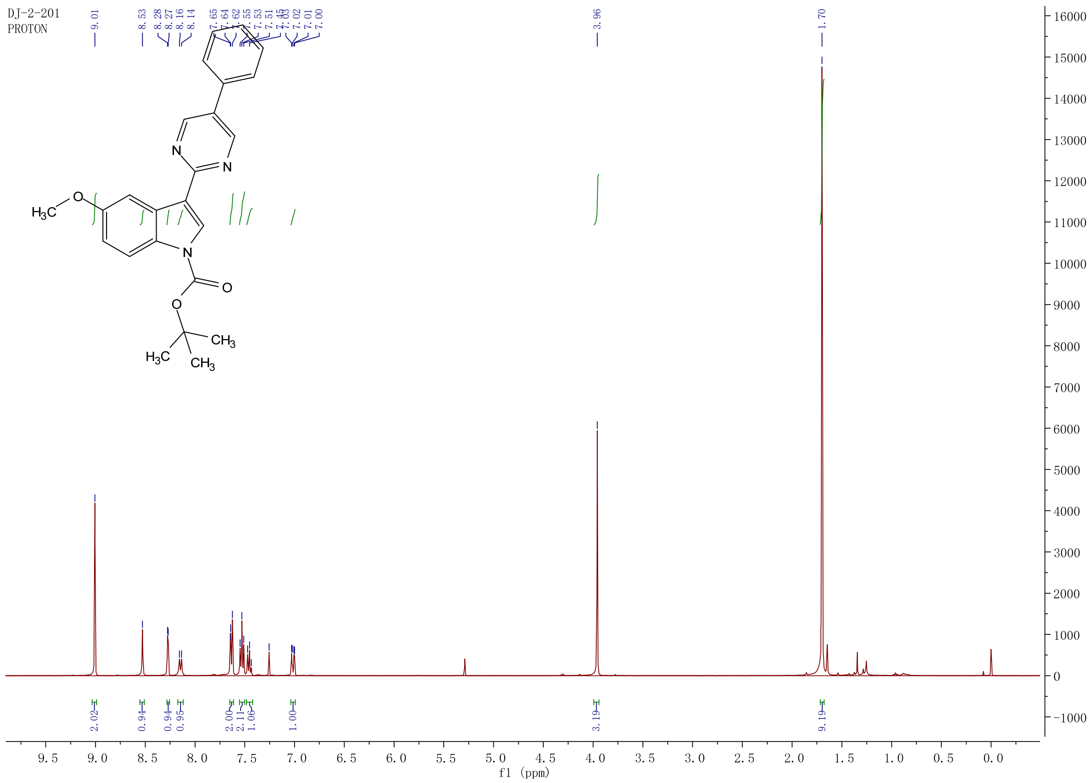

Figure S11. ^{1}H NMR spectrum of 6a

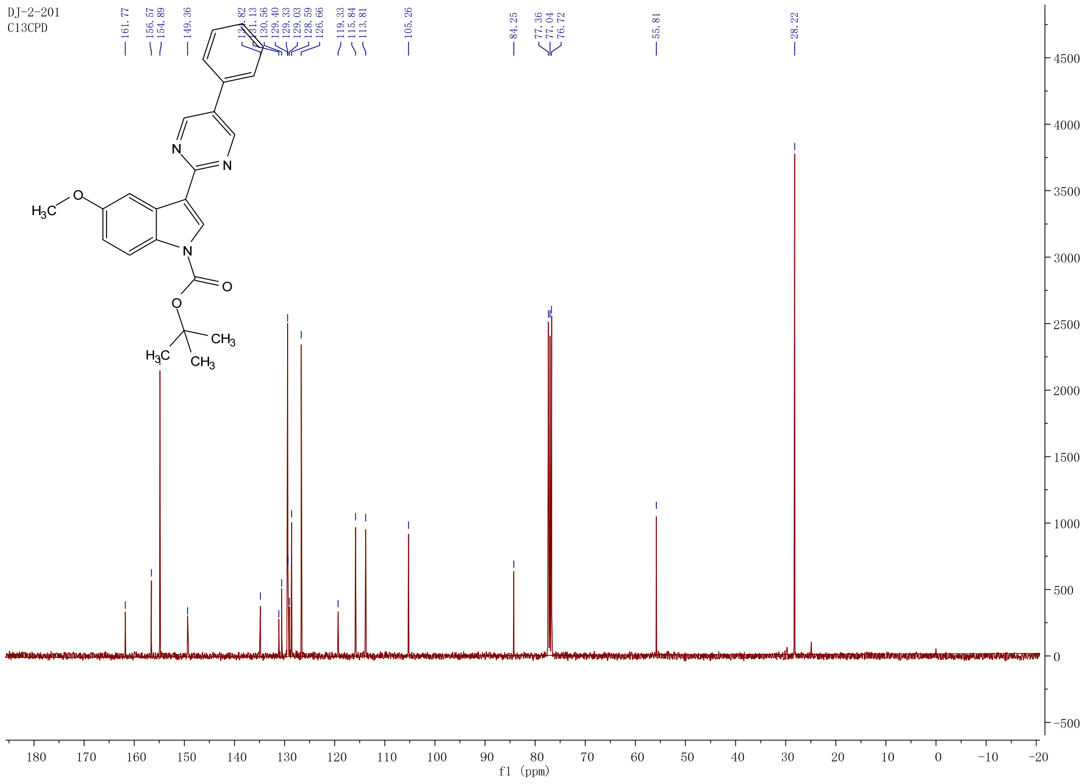

Figure S12. ^{13}C NMR spectrum of 6a

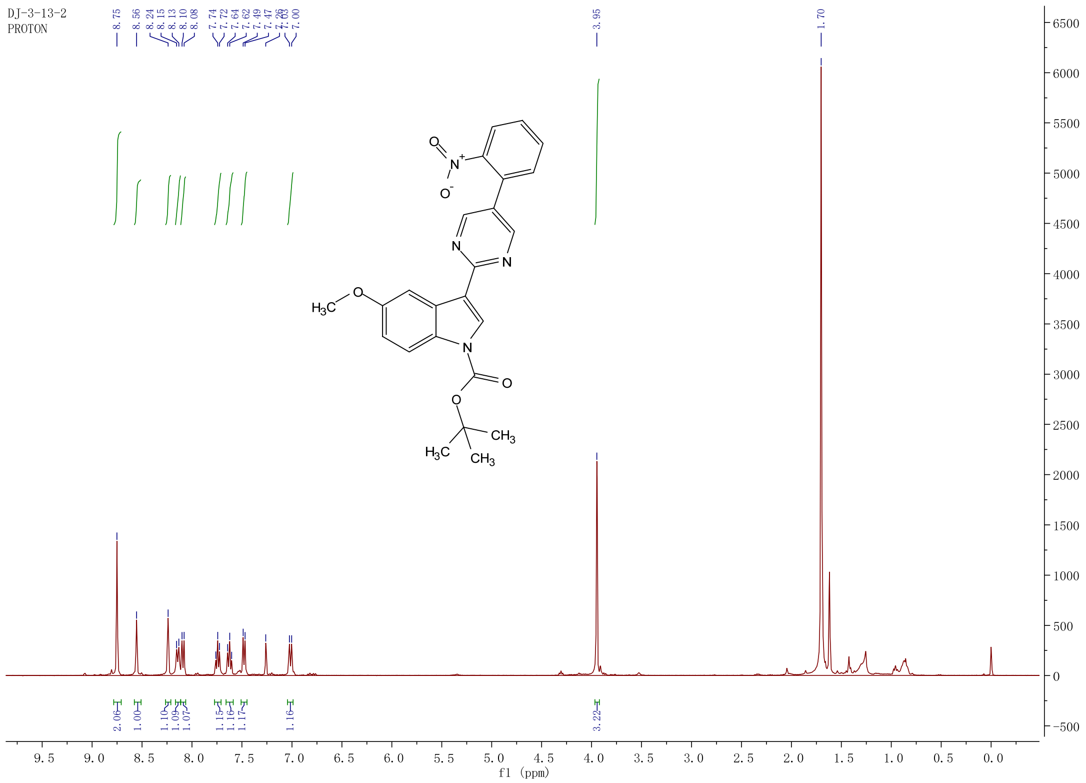

Figure S13. ^{1}H NMR spectrum of 6b

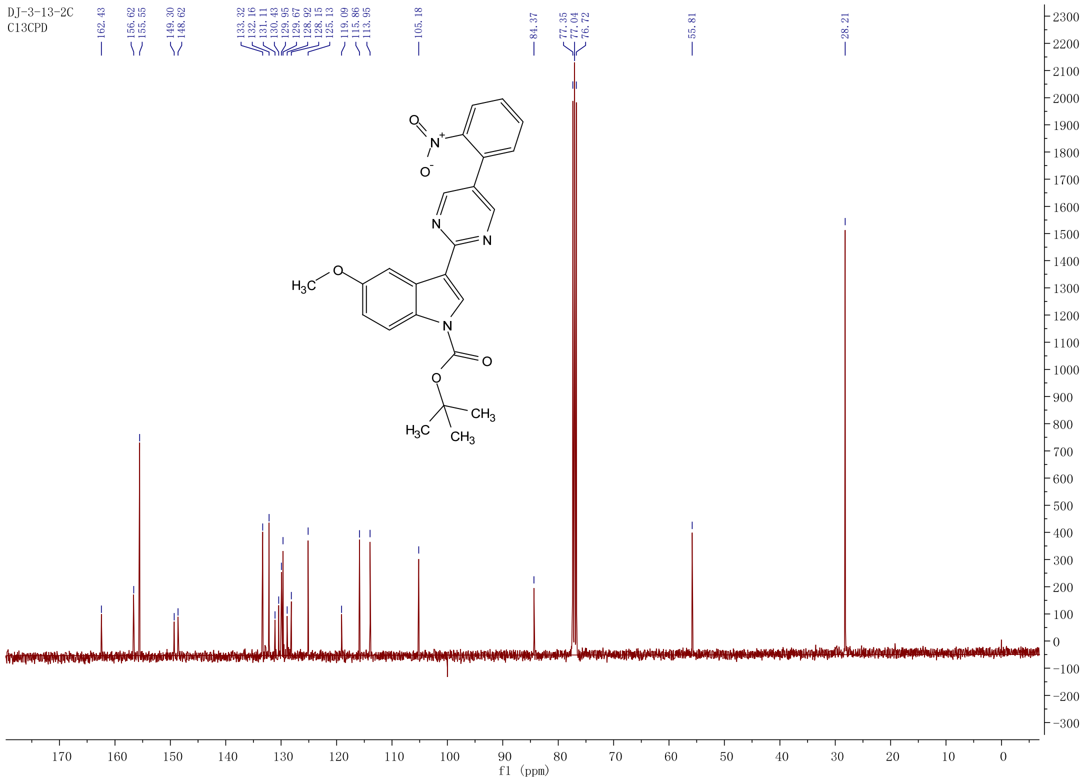

Figure S14. ^{13}C NMR spectrum of 6b

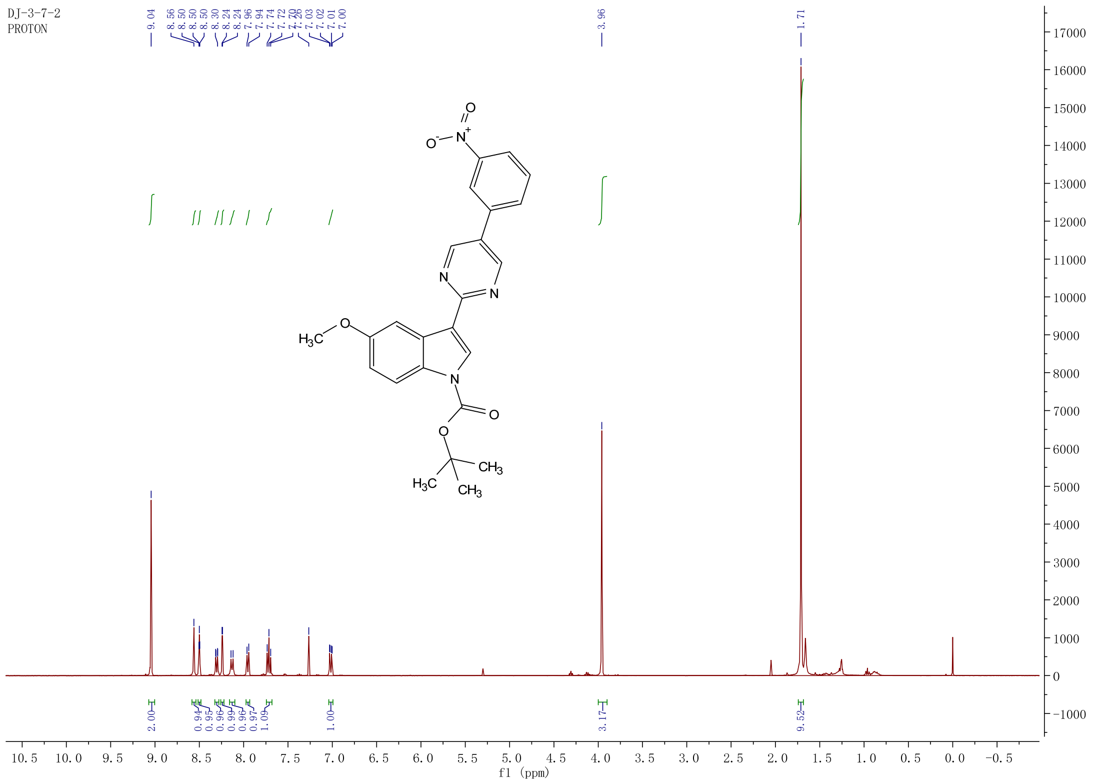

Figure S15. ^{1}H NMR spectrum of 6c

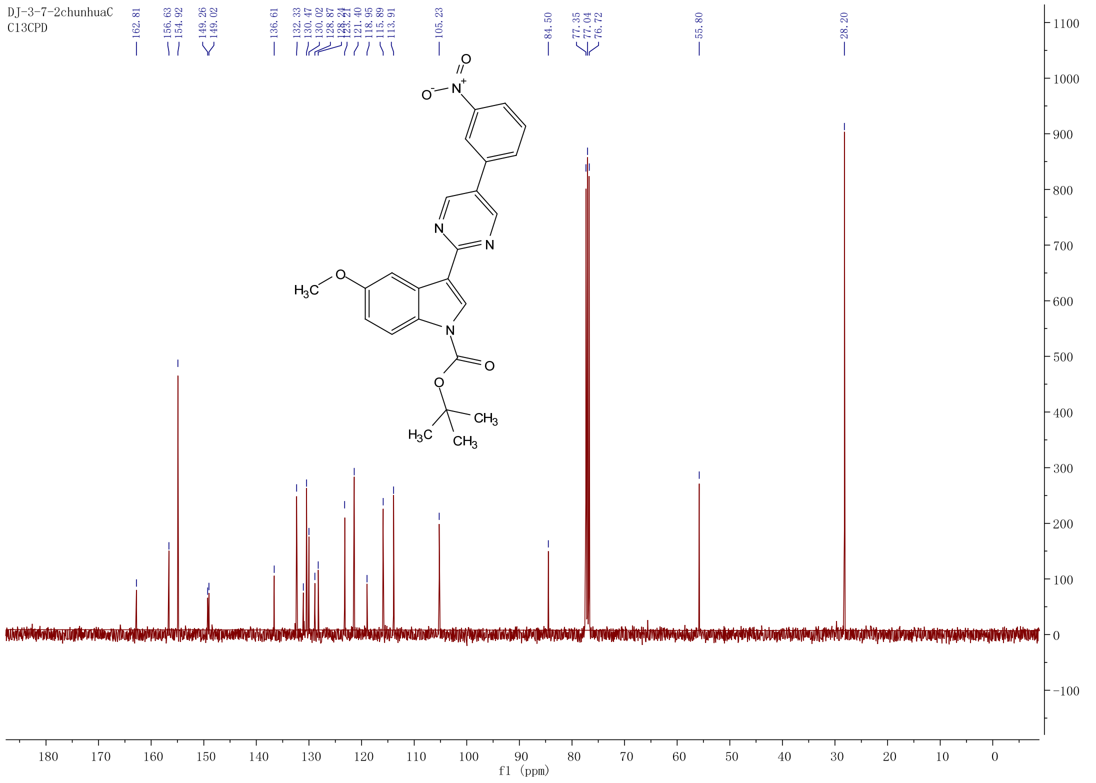

Figure S16. ^{13}C NMR spectrum of 6c

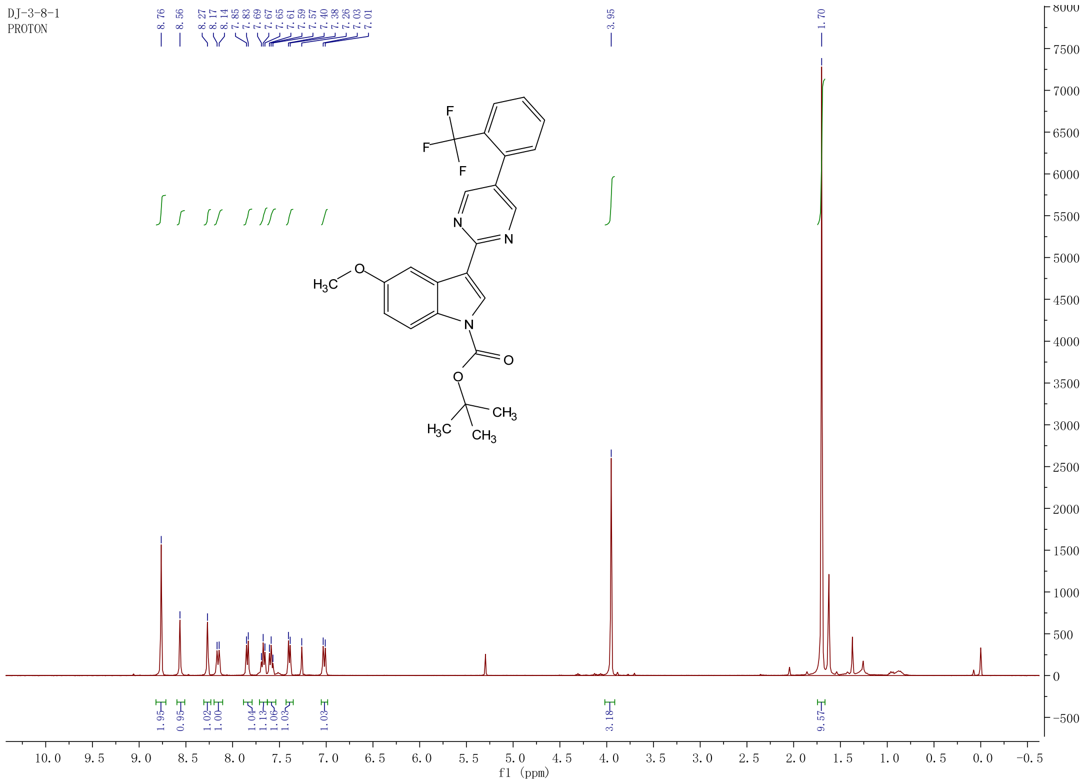

Figure S17. ^{1}H NMR spectrum of 6d

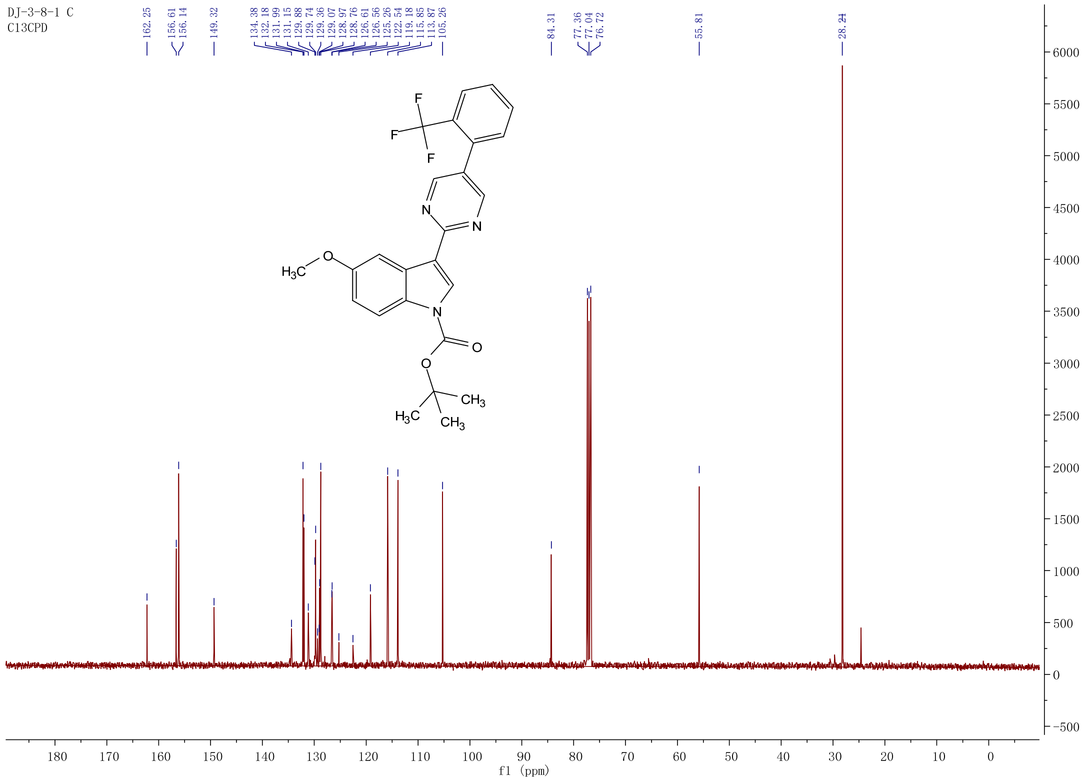

Figure S18. ^{13}C NMR spectrum of 6d

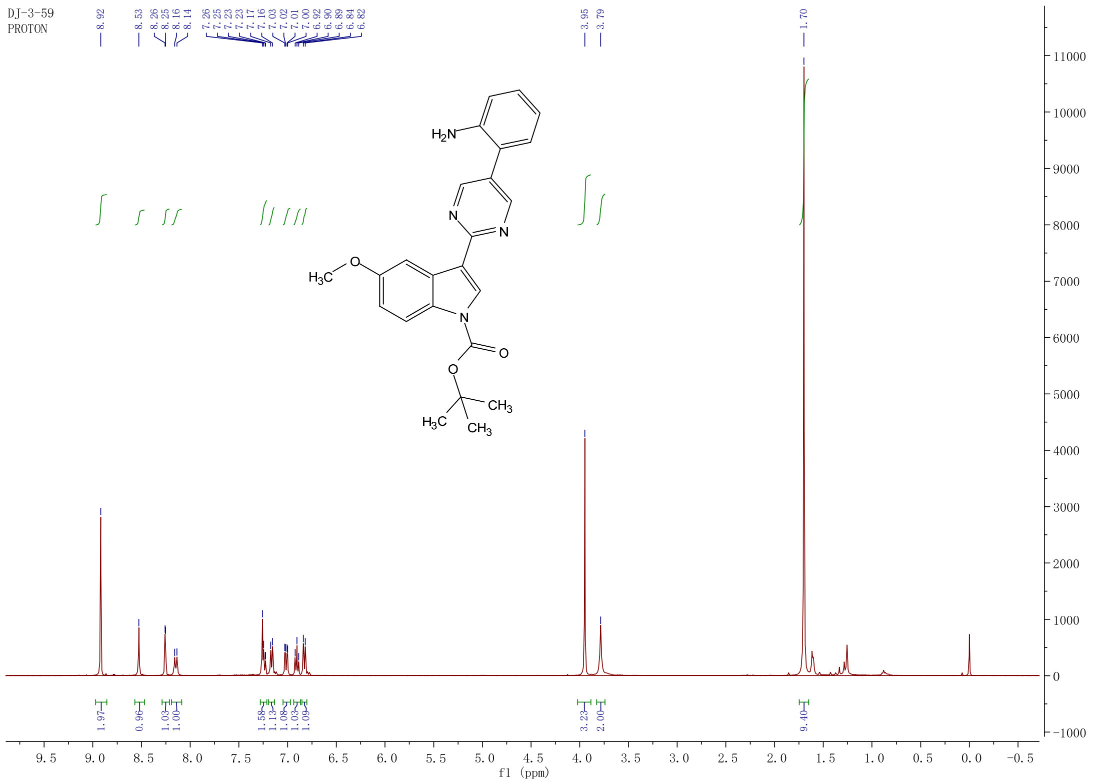

Figure S19. ^{1}H NMR spectrum of 6e

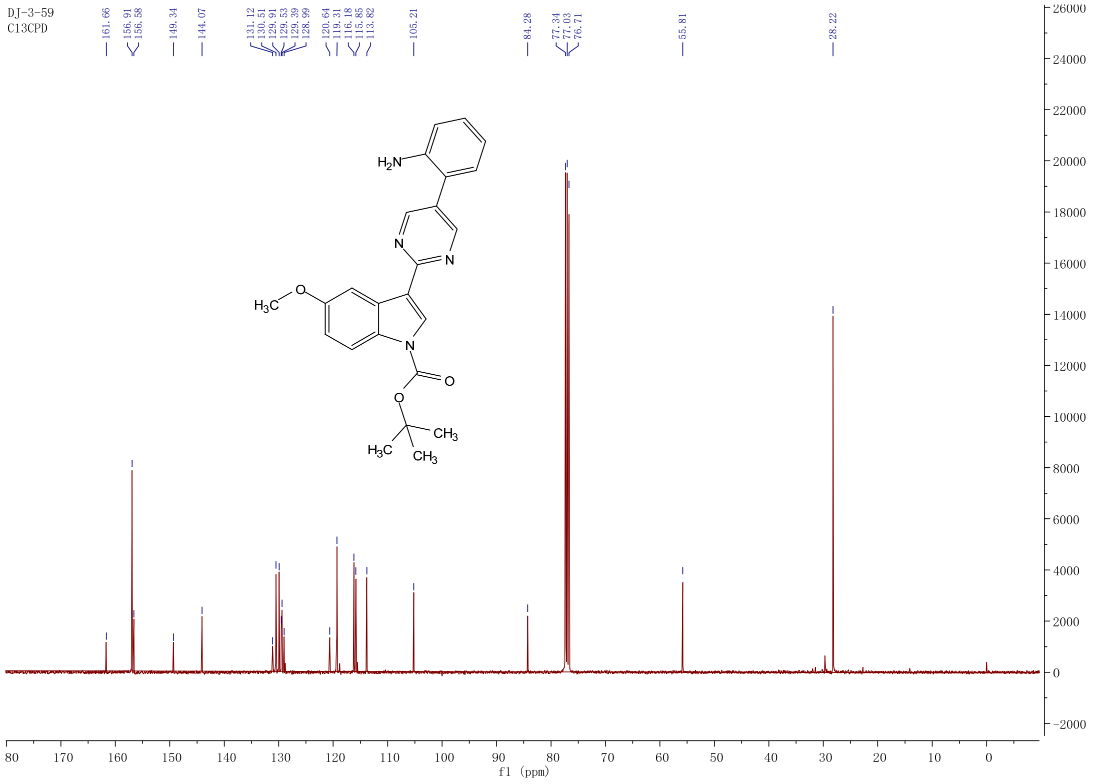

Figure S20. ^{13}C NMR spectrum of 6e

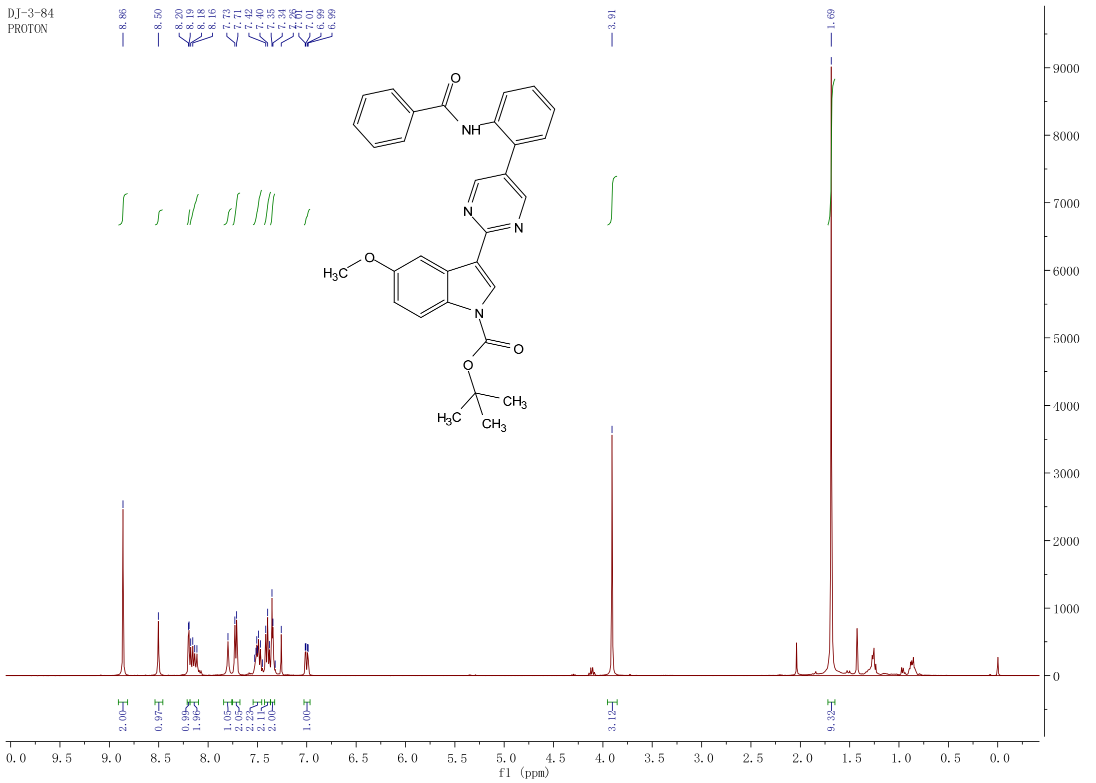

Figure S21. ^{1}H NMR spectrum of 7a

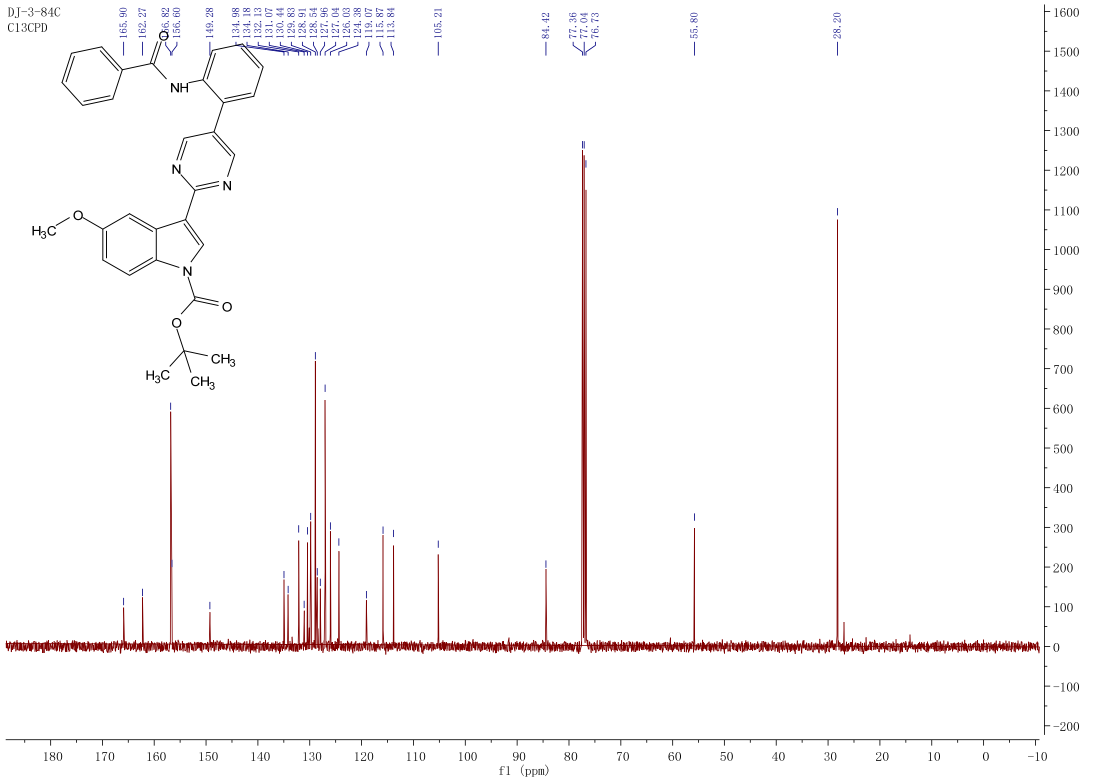

Figure S22. ^{13}C NMR spectrum of 7a

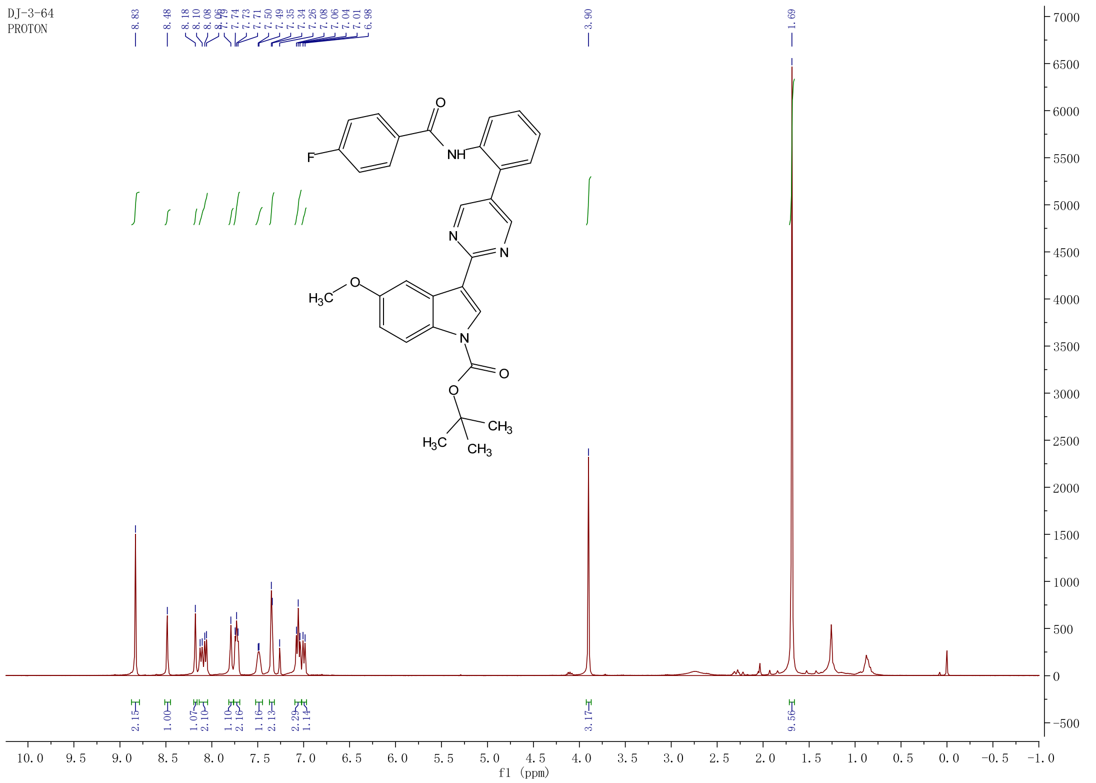

Figure S23. ^{1}H NMR spectrum of 7b

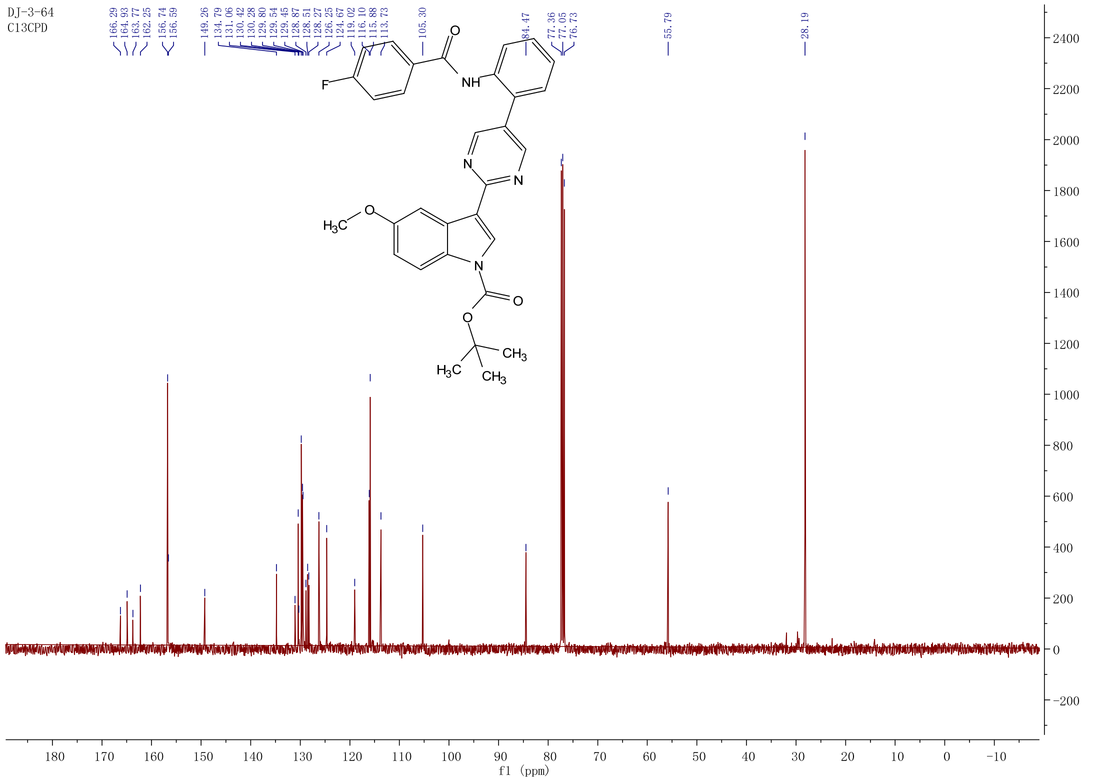

Figure S24. ^{13}C NMR spectrum of 7b

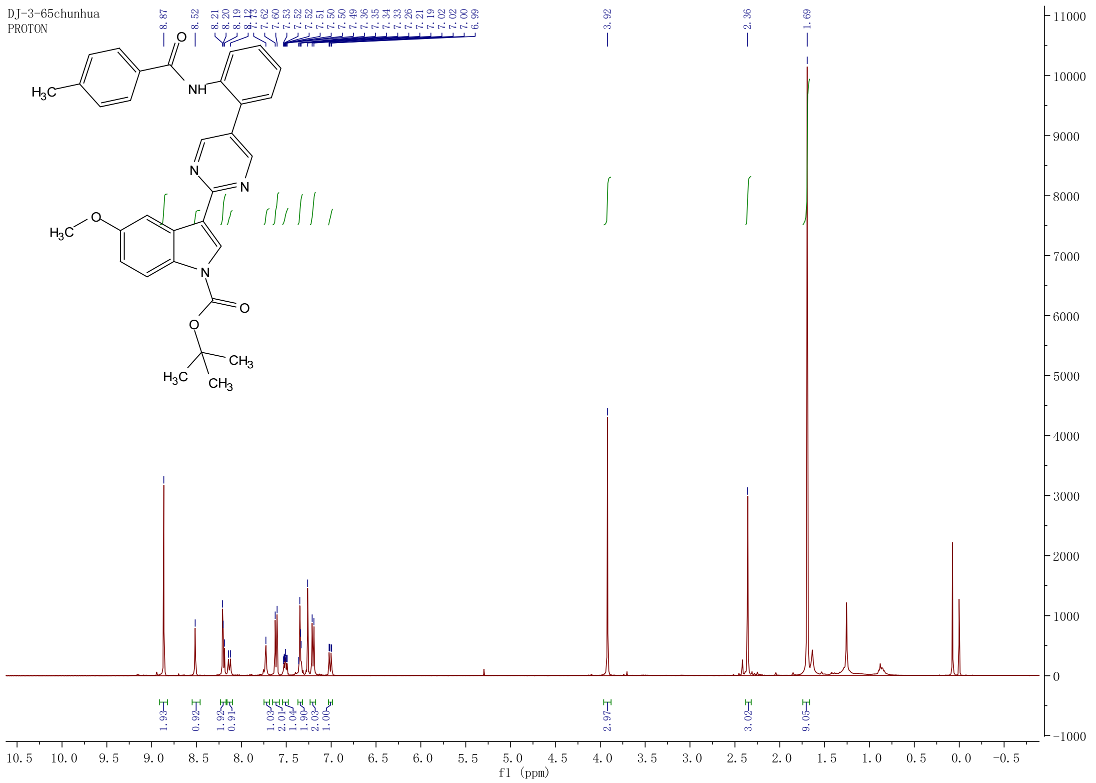

Figure S25. ^{1}H NMR spectrum of 7c

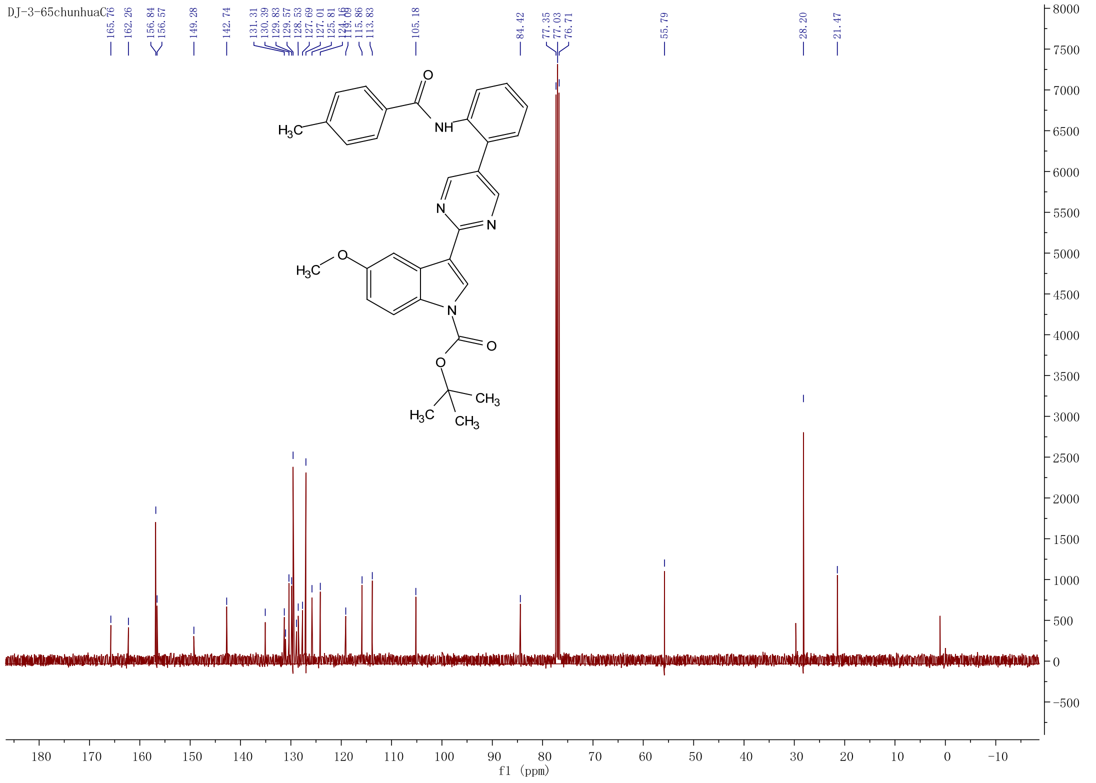

Figure S26. ^{13}C NMR spectrum of 7c

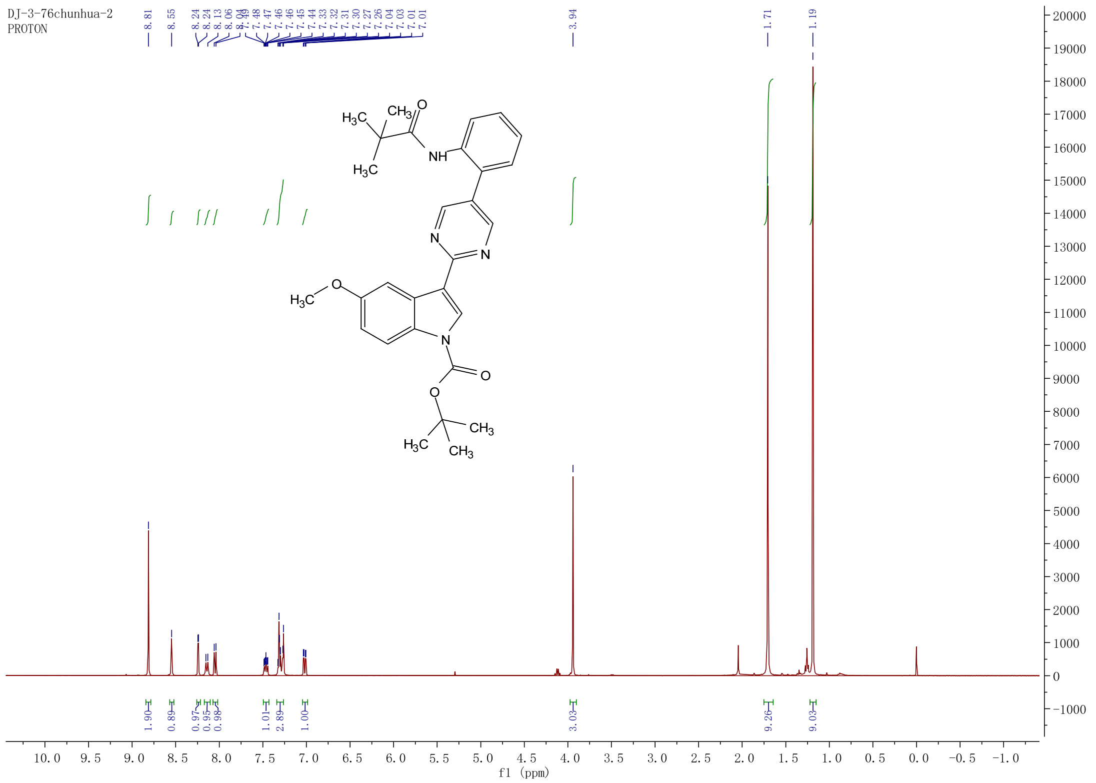

Figure S27. ^{1}H NMR spectrum of 7d

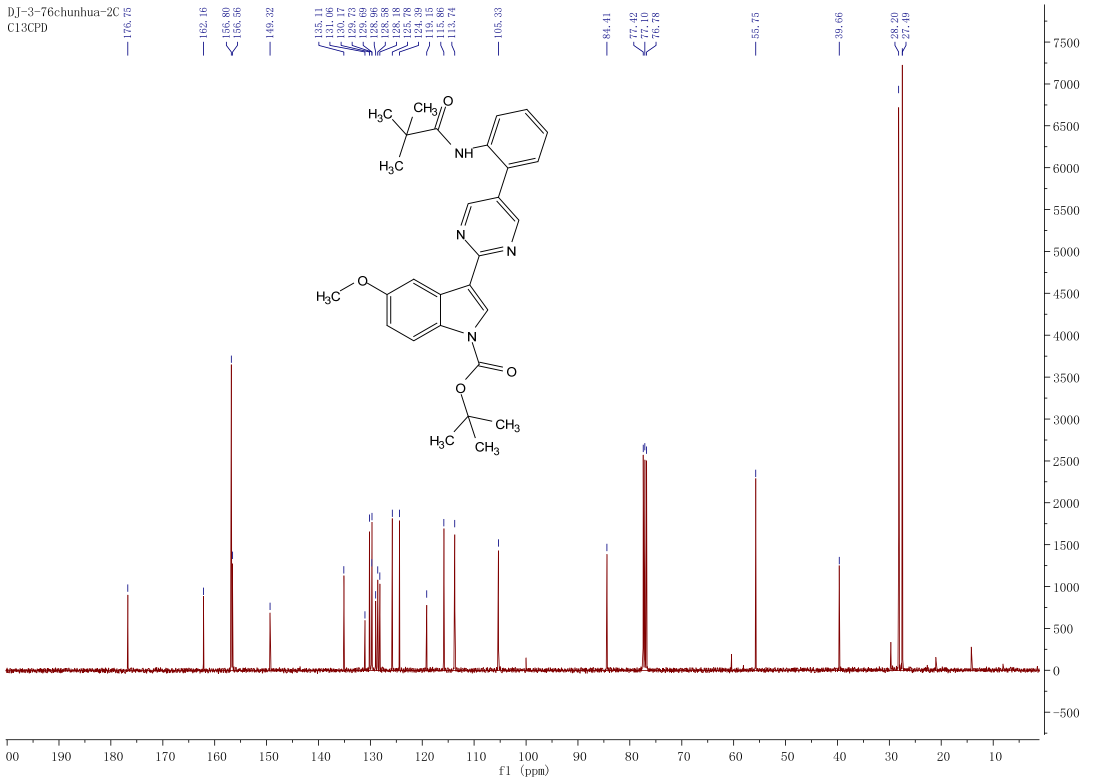

Figure S28. ^{13}C NMR spectrum of 7d

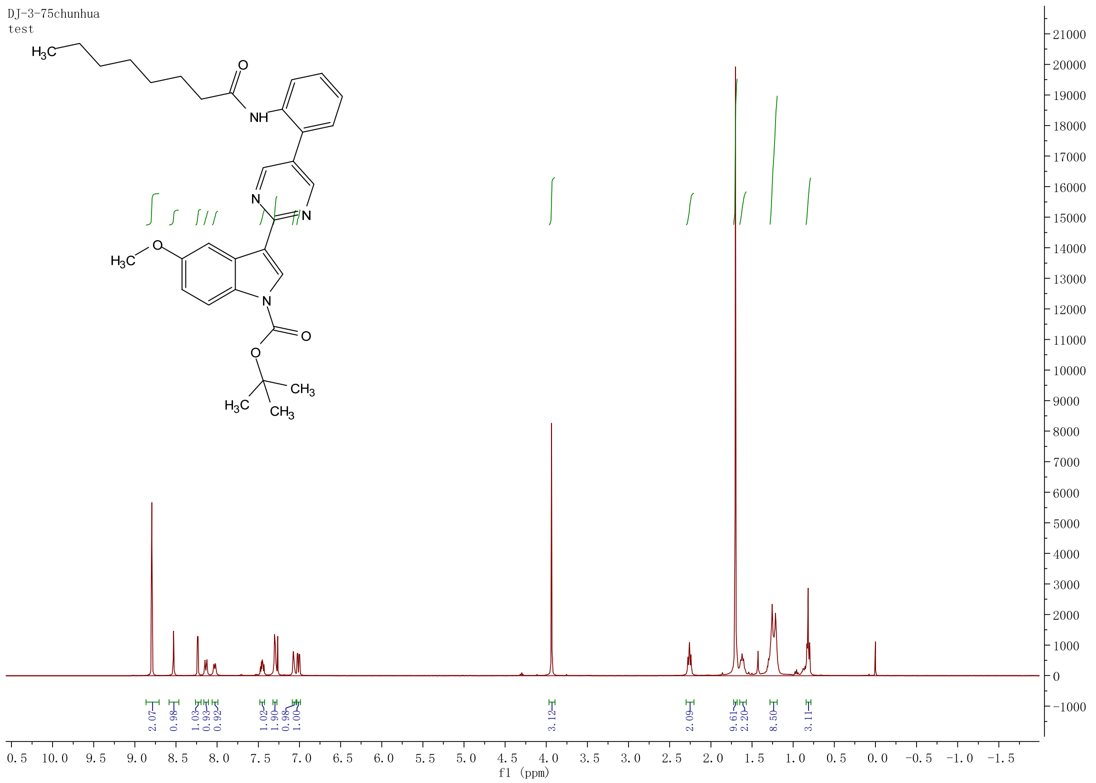

Figure S29. ^{1}H NMR spectrum of 7e

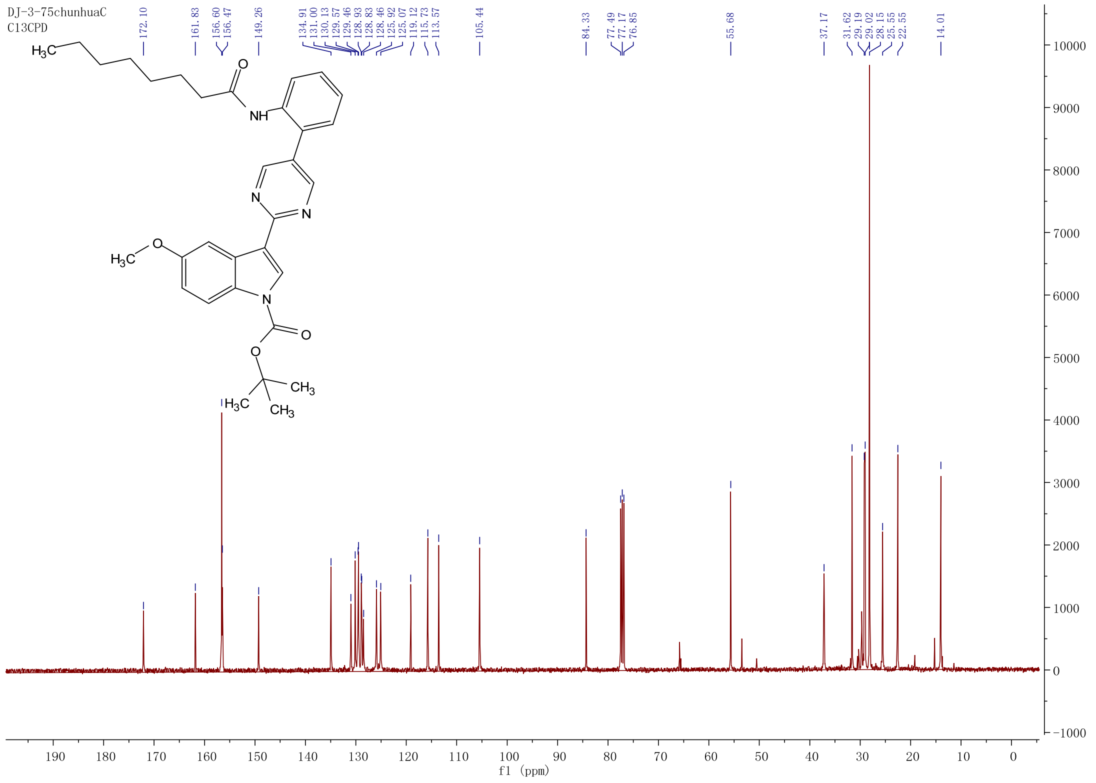

Figure S30. ^{13}C NMR spectrum of 7e

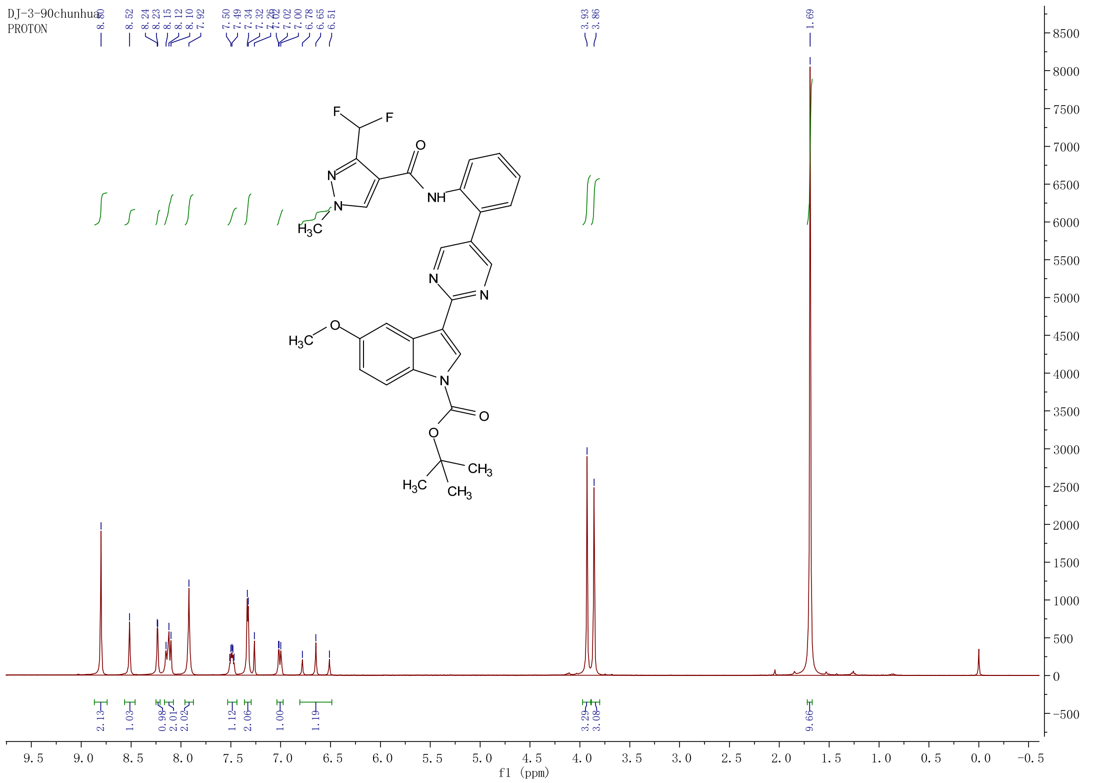

Figure S31. ^{1}H NMR spectrum of 7f

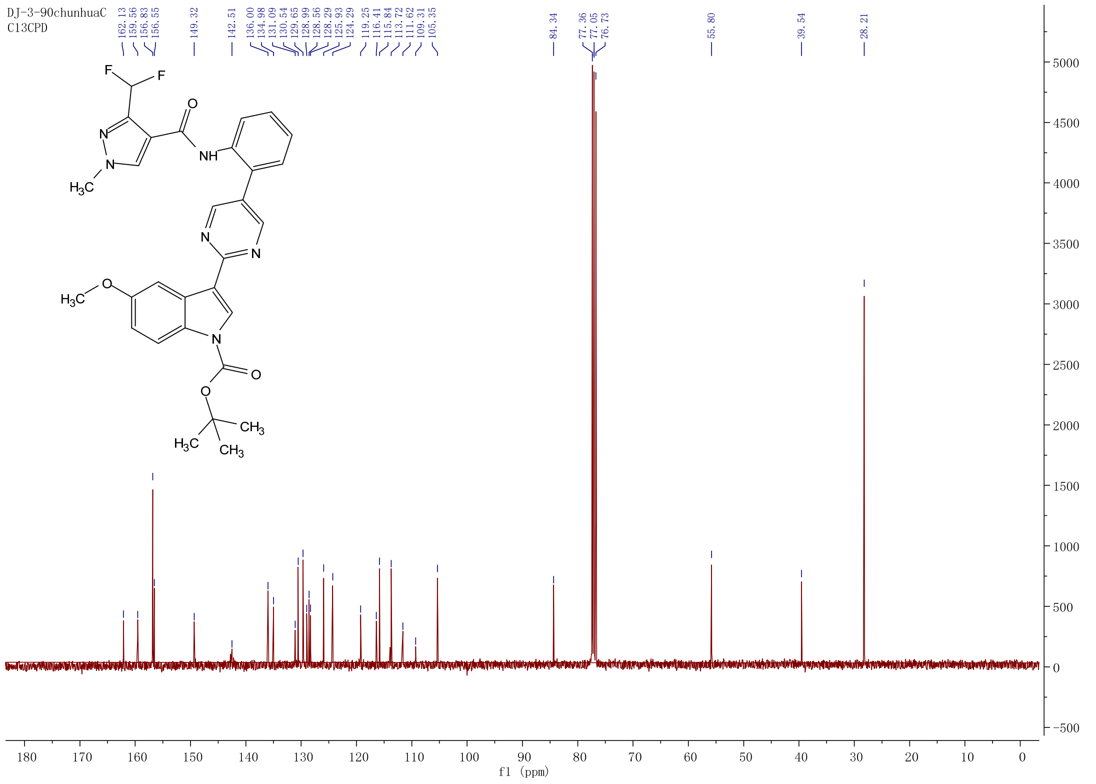

Figure S32. ^{13}C NMR spectrum of 7f

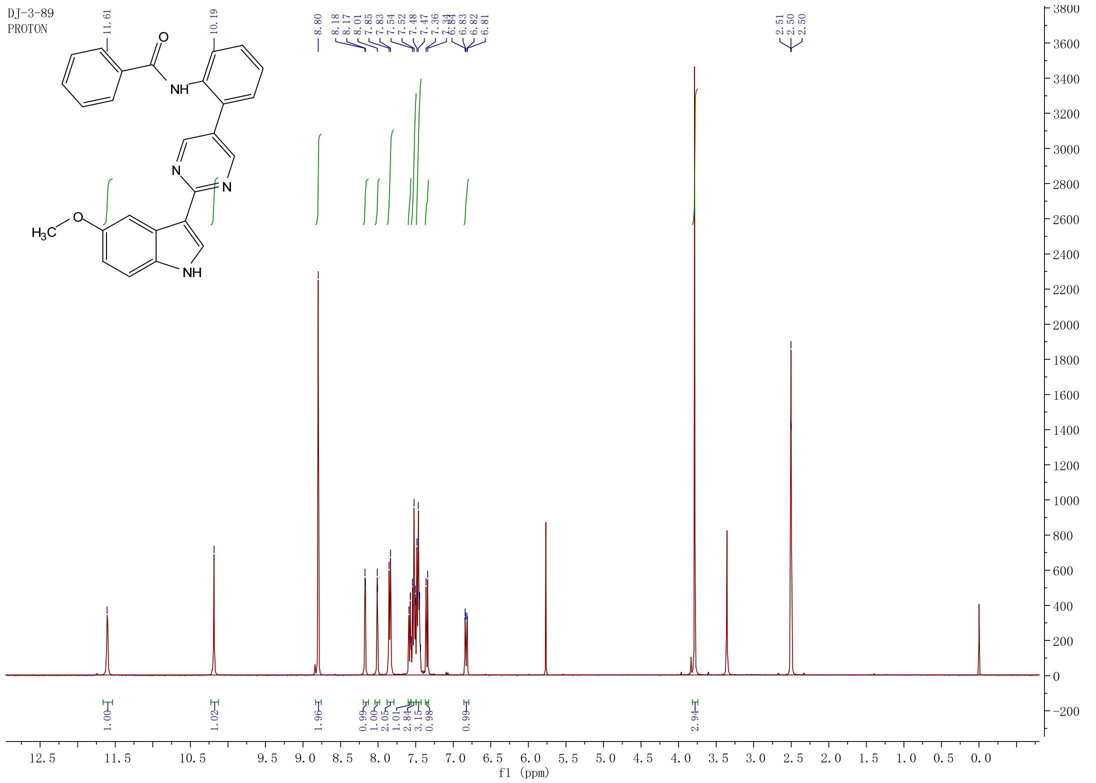

Figure S33. ^{1}H NMR spectrum of 8a

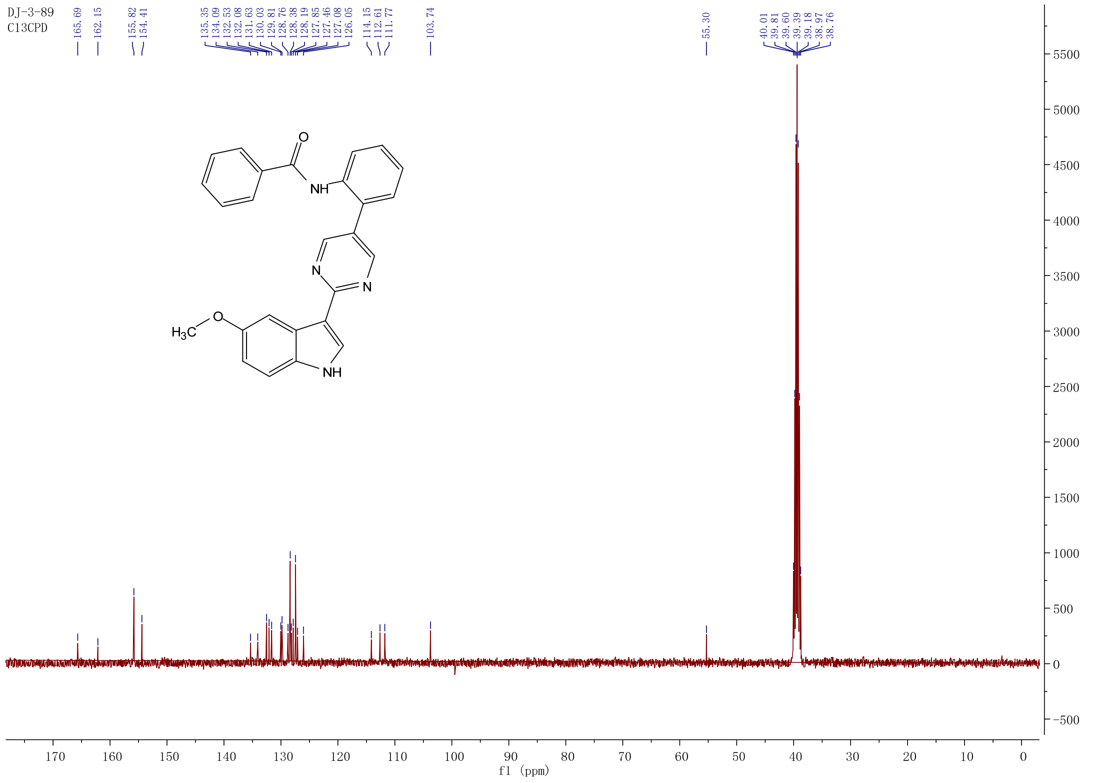

Figure S34. ^{13}C NMR spectrum of 8a

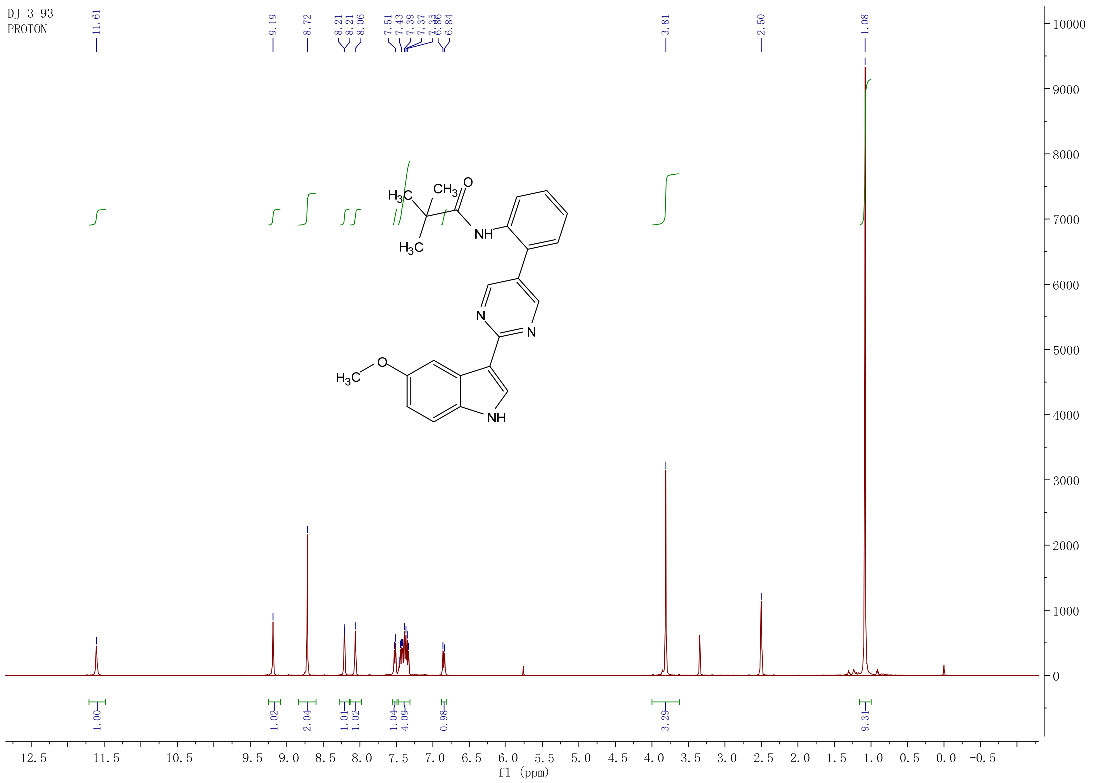

Figure S35. ^{1}H NMR spectrum of 8b

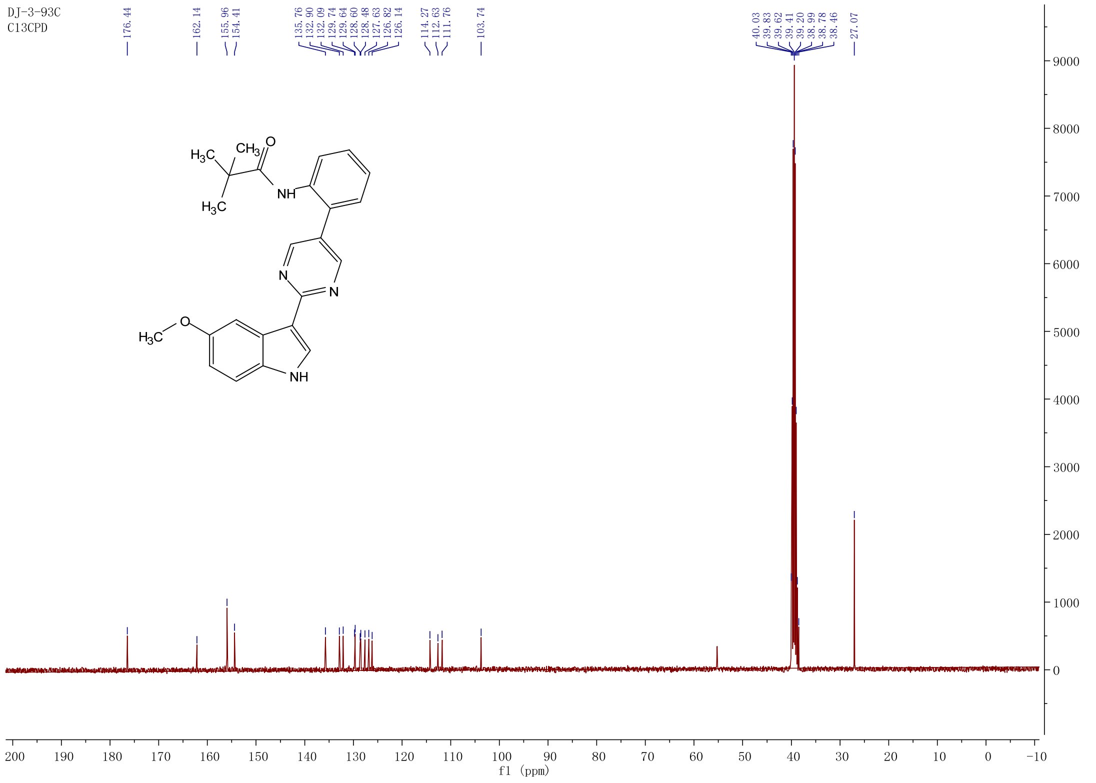

Figure S36. ^{13}C NMR spectrum of 8b

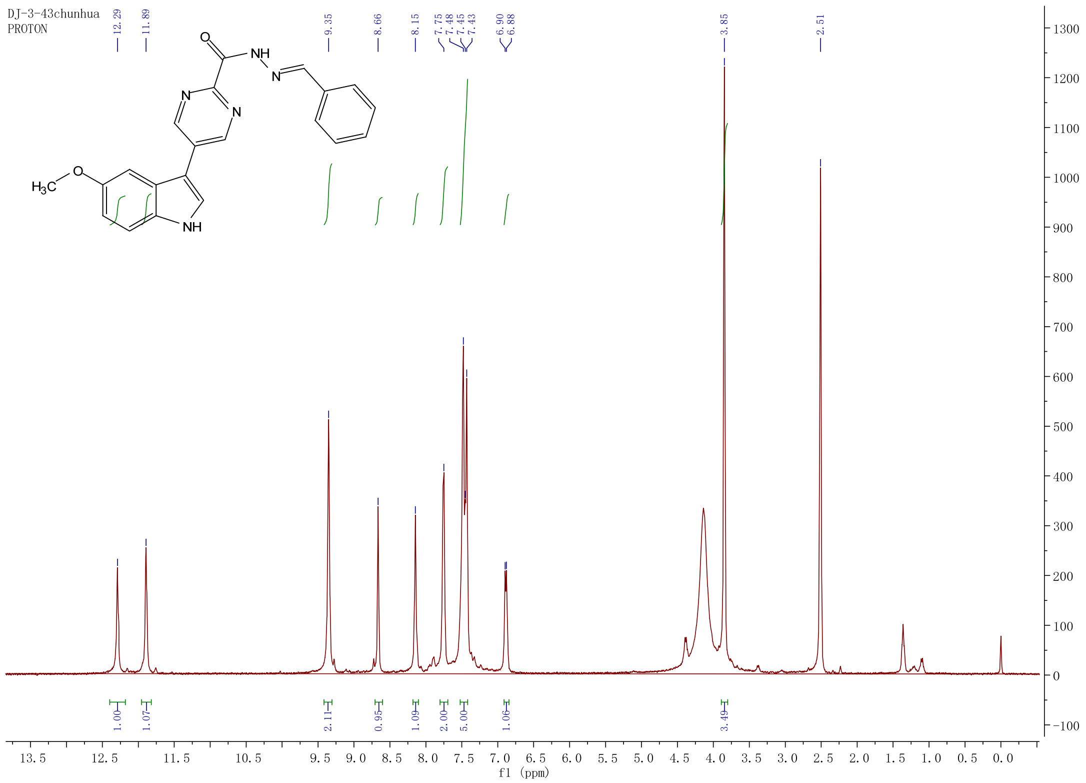

Figure S37. ^{1}H NMR spectrum of 9a

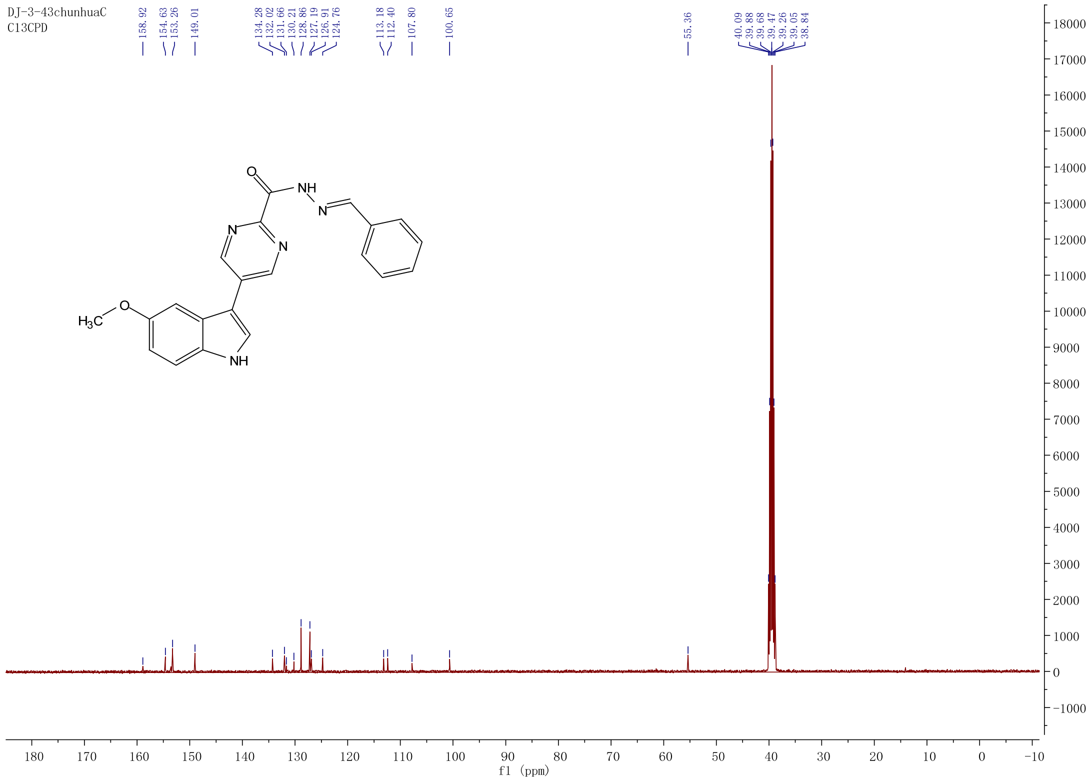

Figure S38. ^{13}C NMR spectrum of 9a

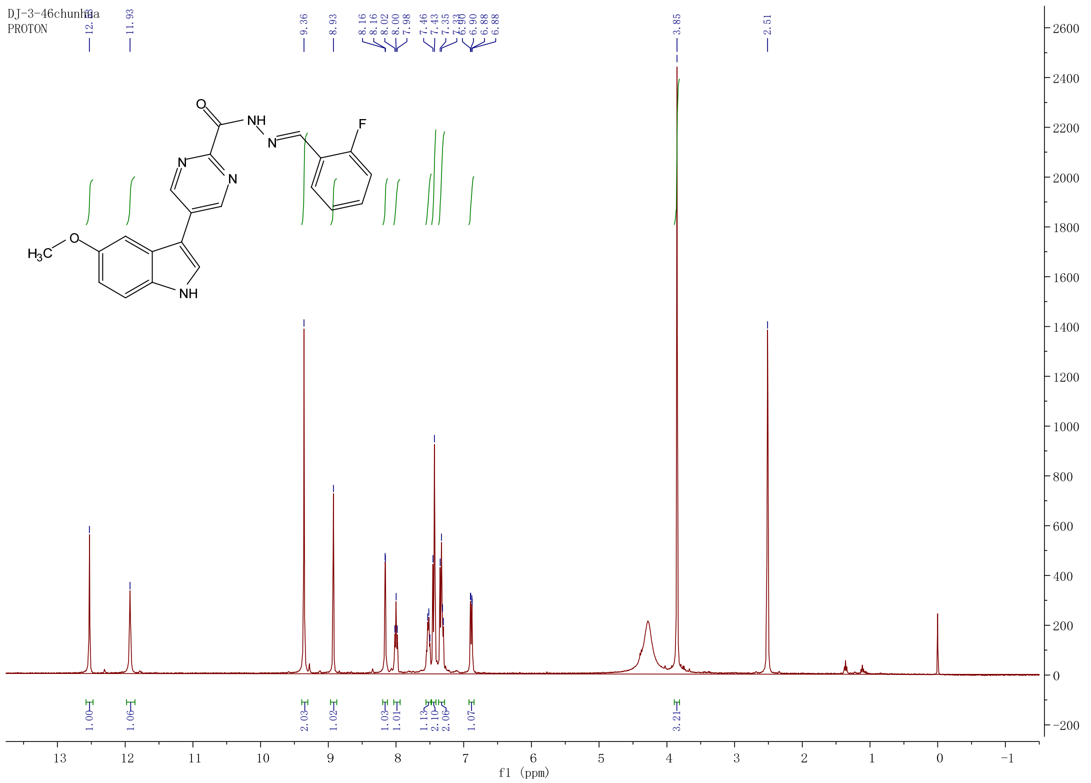

Figure S39. ^{1}H NMR spectrum of 9b

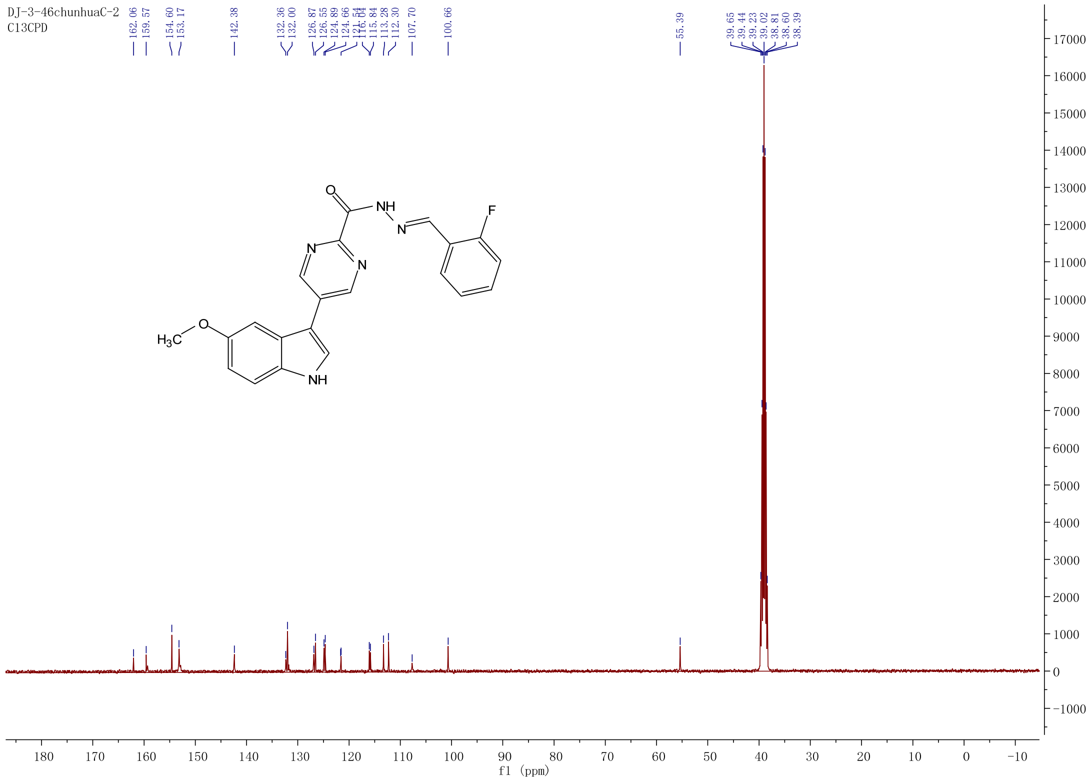

Figure S40. ^{13}C NMR spectrum of 9b

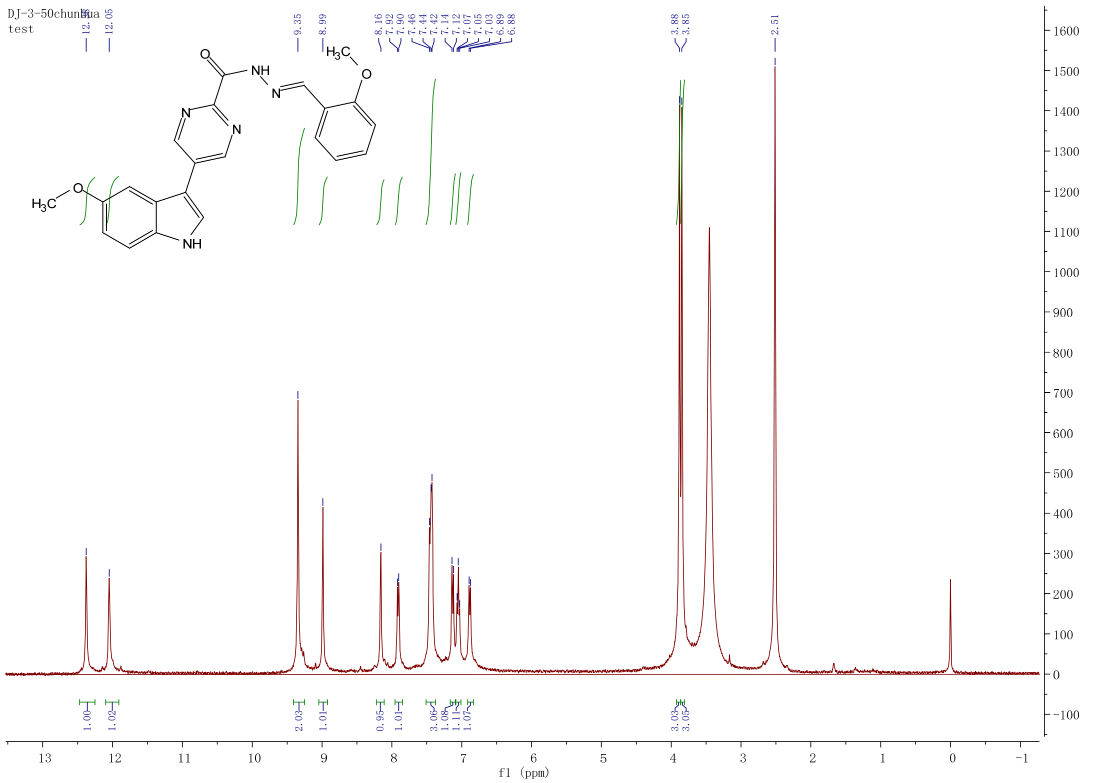

Figure S41. ^{1}H NMR spectrum of 9c

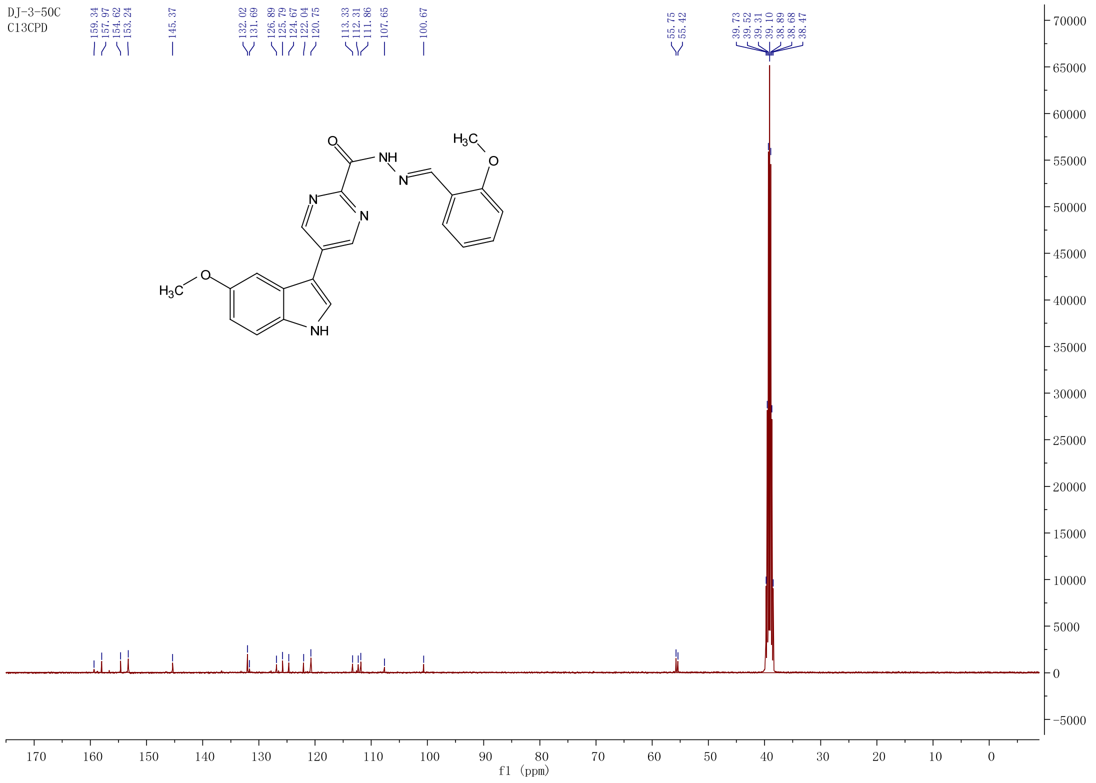

Figure S42. ^{13}C NMR spectrum of 9c

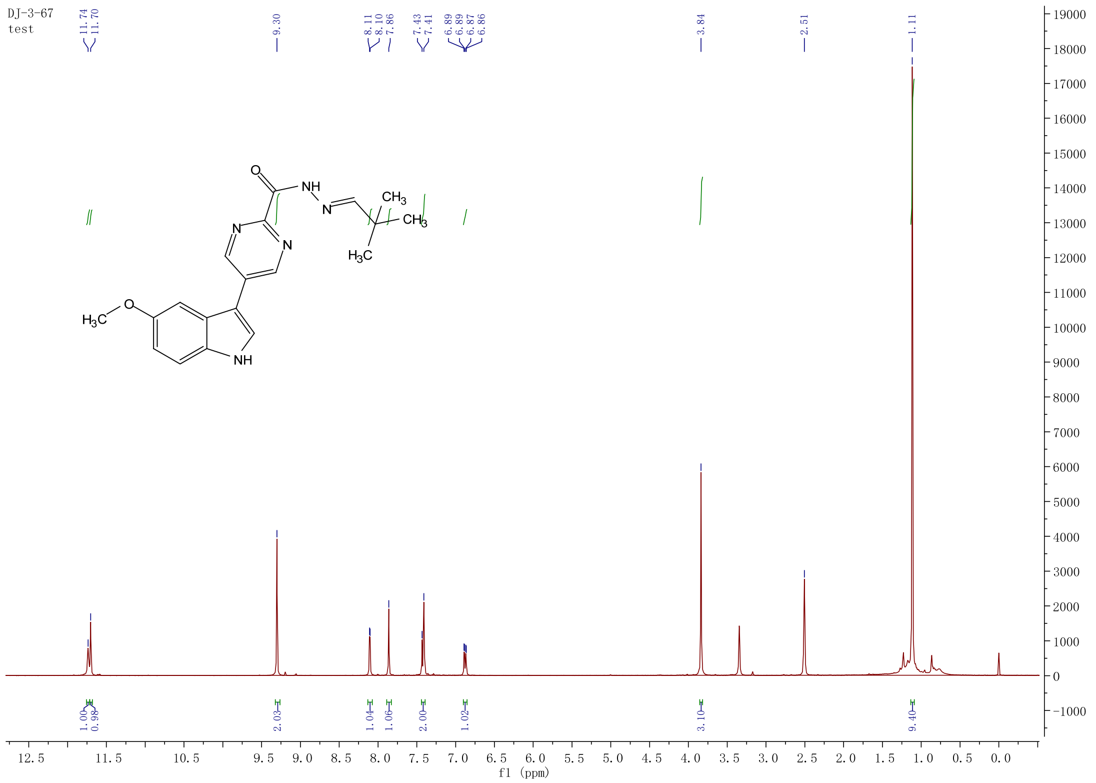

Figure S43. ^{1}H NMR spectrum of 9d

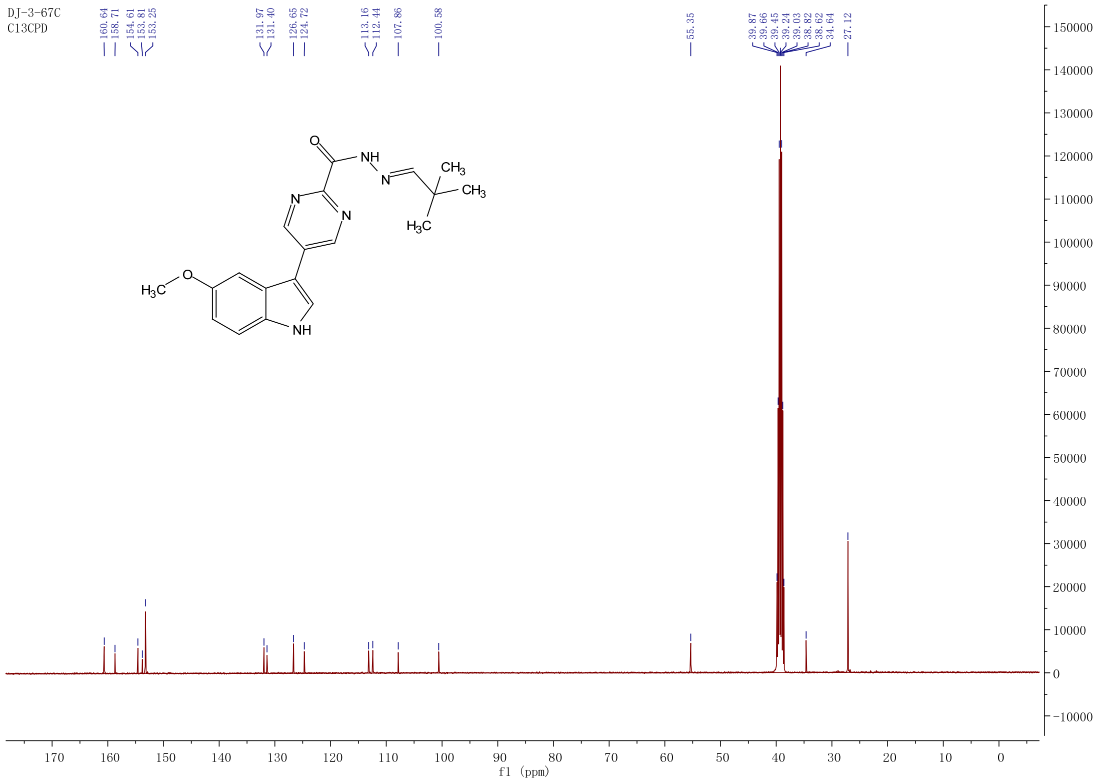

Figure S44. ^{13}C NMR spectrum of 9d

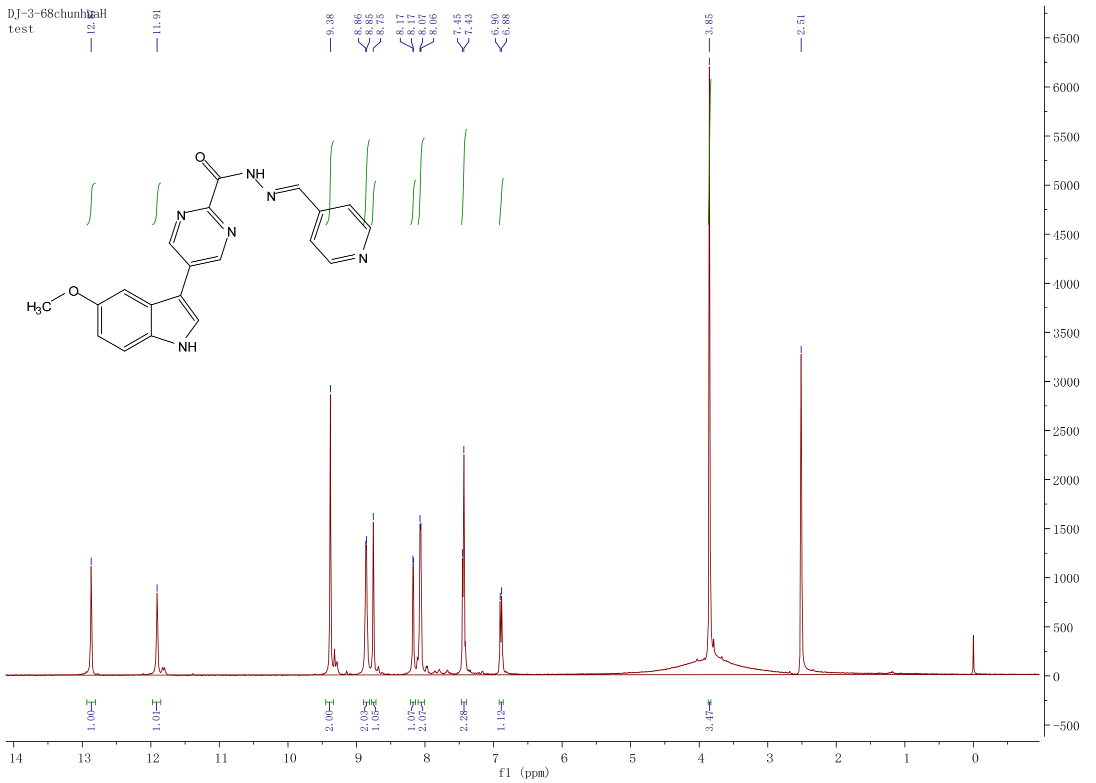

Figure S45. ^{1}H NMR spectrum of 9e

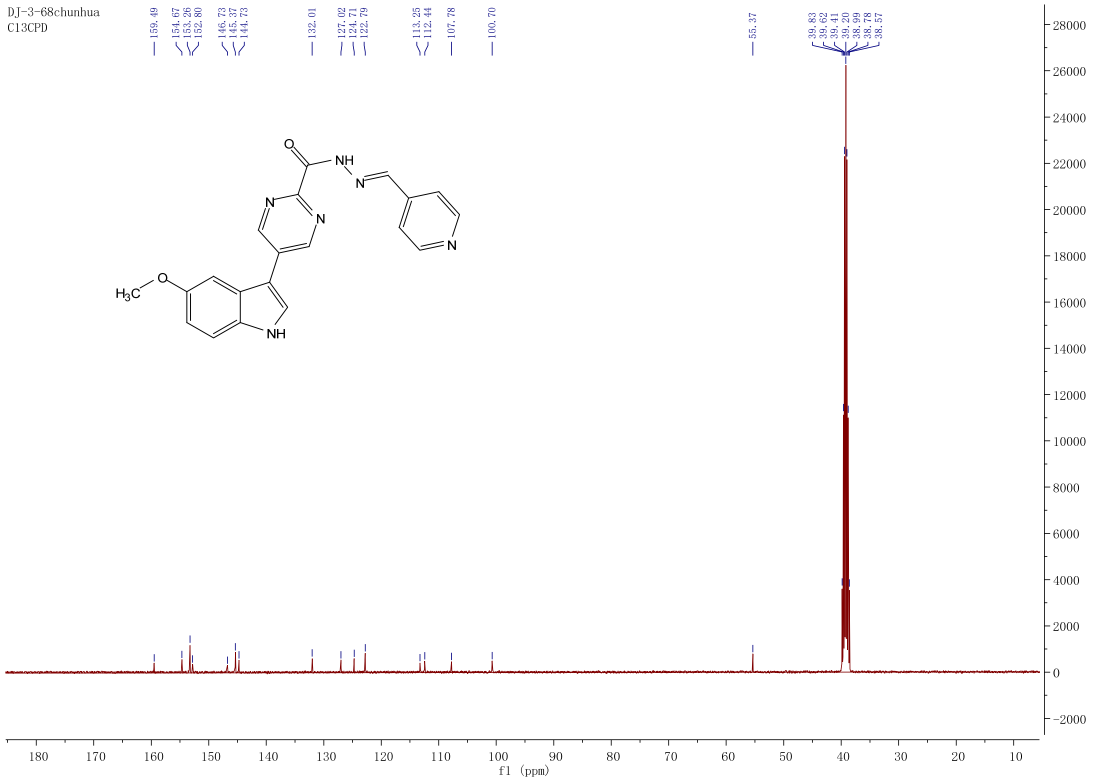

Figure S46. ^{13}C NMR spectrum of 9e

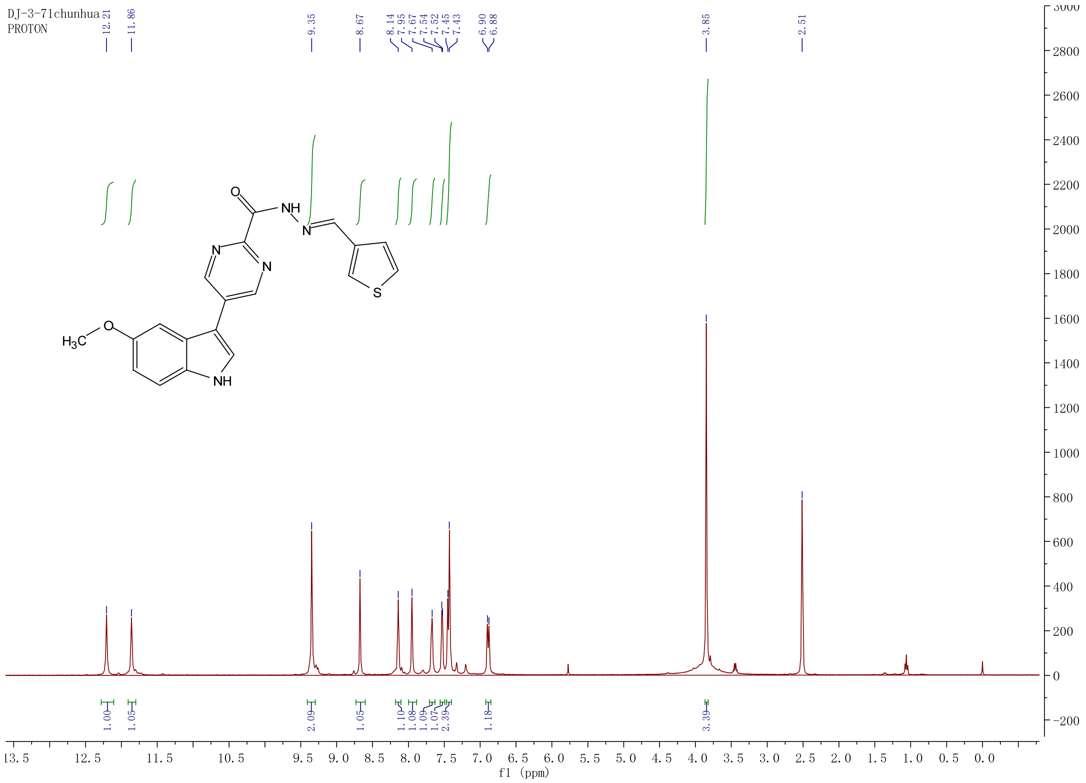

Figure S47. ^{1}H NMR spectrum of 9f

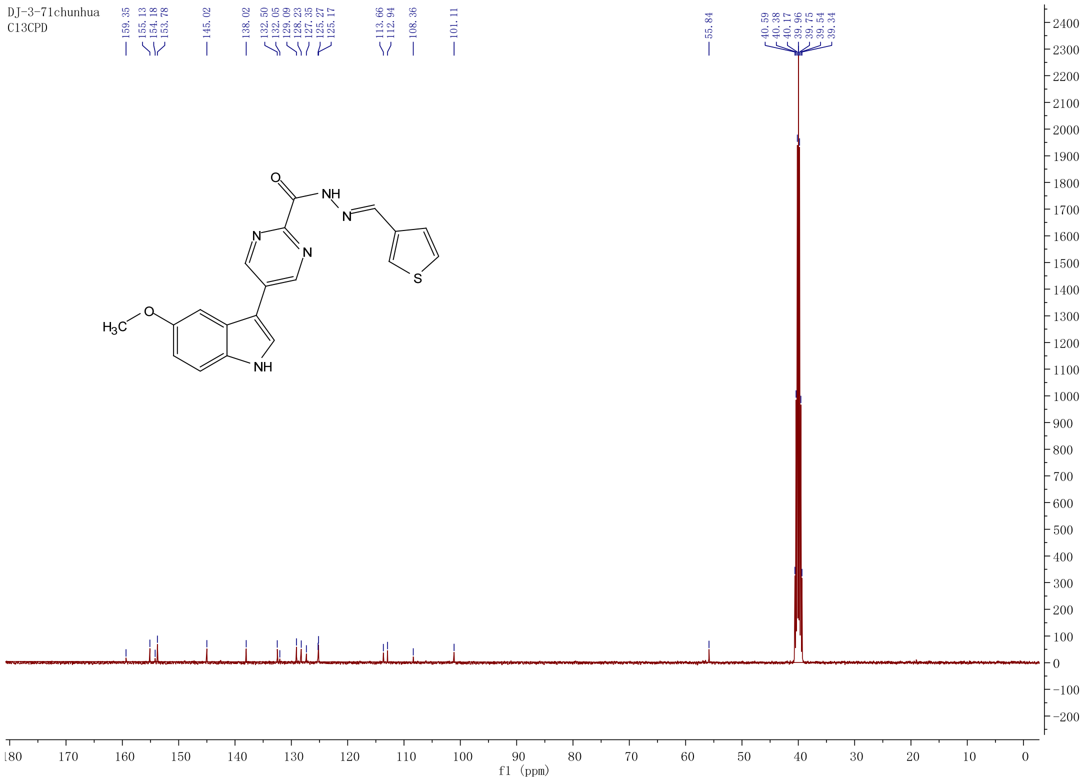

Figure S48. ^{13}C NMR spectrum of 9f

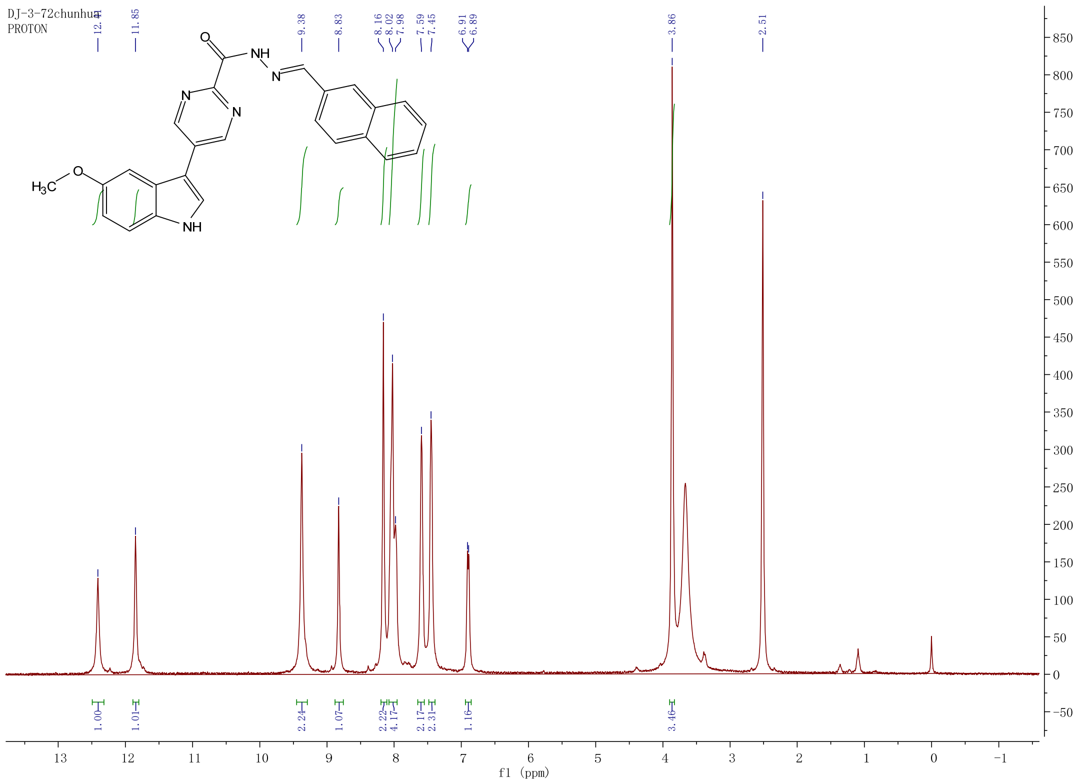

Figure S49. ^{1}H NMR spectrum of 9g

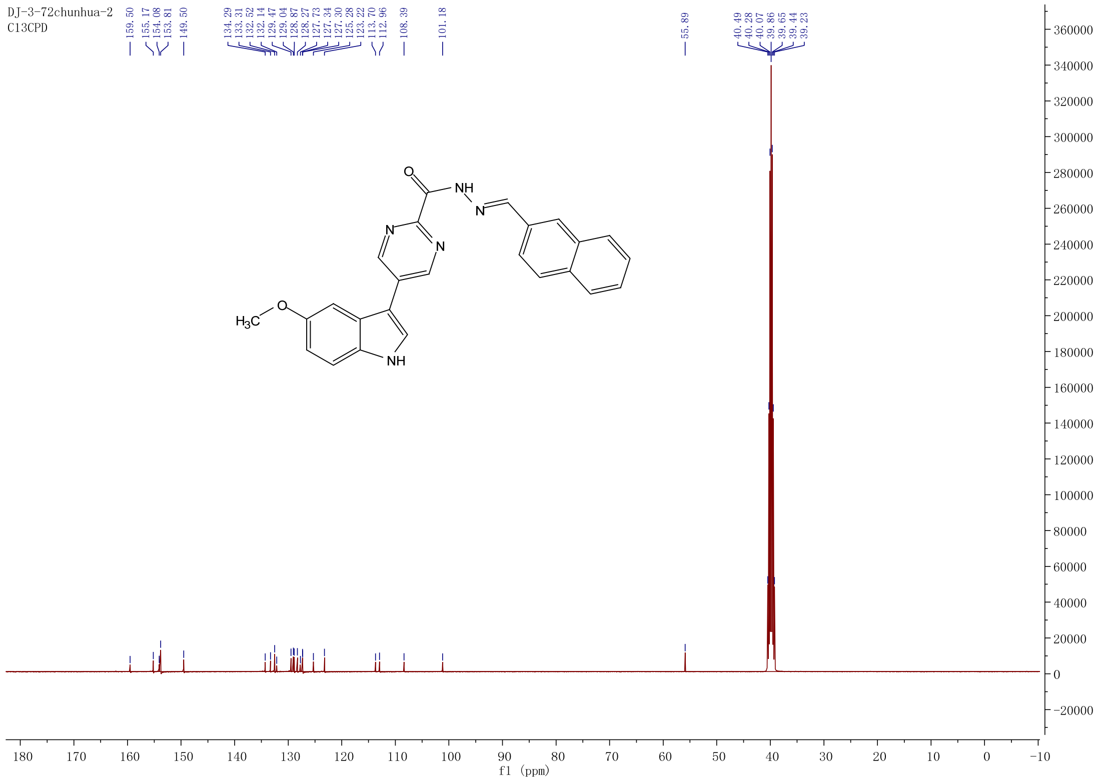

Figure S50. ^{13}C NMR spectrum of 9g
